# Ex-situ generation and synthetic utilization of bare trifluoromethyl anion in flow via rapid biphasic mixing

**DOI:** 10.1038/s41467-022-35611-9

**Published:** 2023-03-03

**Authors:** Hyune-Jea Lee, Jeong-Un Joo, Se-Jun Yim, Dong-Pyo Kim, Heejin Kim

**Affiliations:** 1 Department of Chemistry, College of Science, Korea University, 145 Anam-ro, Seongbuk-gu, Seoul, 02841 Republic of Korea; 2 Department of Chemical Engineering, Pohang University of Science and Technology, 77 Cheongam-ro, Nam-gu, Pohang-si, Gyeongsangbuk-do 37673 Republic of Korea; 3 Present Address: Samsung Advanced Institute of Technology, 130 Samsung-ro, Yeongtong-gu, Suwon-si, Gyeonggi-do 16678 Republic of Korea; 4 Present Address: Department of Chemical Engineering, Pohang University of Science and Technology, 77 Cheongam-ro, Nam-gu, Pohang-si, Gyeongsangbuk-do 37673 Republic of Korea

**Keywords:** Flow chemistry, Synthetic chemistry methodology, Chemical engineering

## Abstract

Fluoroform (CF_3_H) is the simplest reagent for nucleophilic trifluoromethylation intermediated by trifluoromethyl anion (CF_3_^–^). However, it has been well-known that CF_3_^–^ should be generated in presence of a stabilizer or reaction partner (in-situ method) due to its short lifetime, which results in the fundamental limitation on its synthetic utilization. We herein report a bare CF_3_^–^ can be ex-situ generated and directly used for the synthesis of diverse trifluoromethylated compounds in a devised flow dissolver for rapid biphasic mixing of gaseous CF_3_H and liquid reagents that was designed and structurally optimized by computational fluid dynamics (CFD). In flow, various substrates including multi-functional compounds were chemoselectively reacted with CF_3_^–^, extending to the multi-gram-scale synthesis of valuable compounds by 1-hour operation of the integrated flow system.

## Abstract (web summary)

The trifluoromethyl anion rapidly decomposes into difluorocarbene (:CF_2_) and fluorine ion, limiting its applicability in synthesis. Here, the authors report a strategy to generate and use the short-lived CF_3_- intermediate from stable CF_3_H gas via fast biphasic mixing in precisely customized flow dissolvers.

## Introduction

**Fig. 1:**
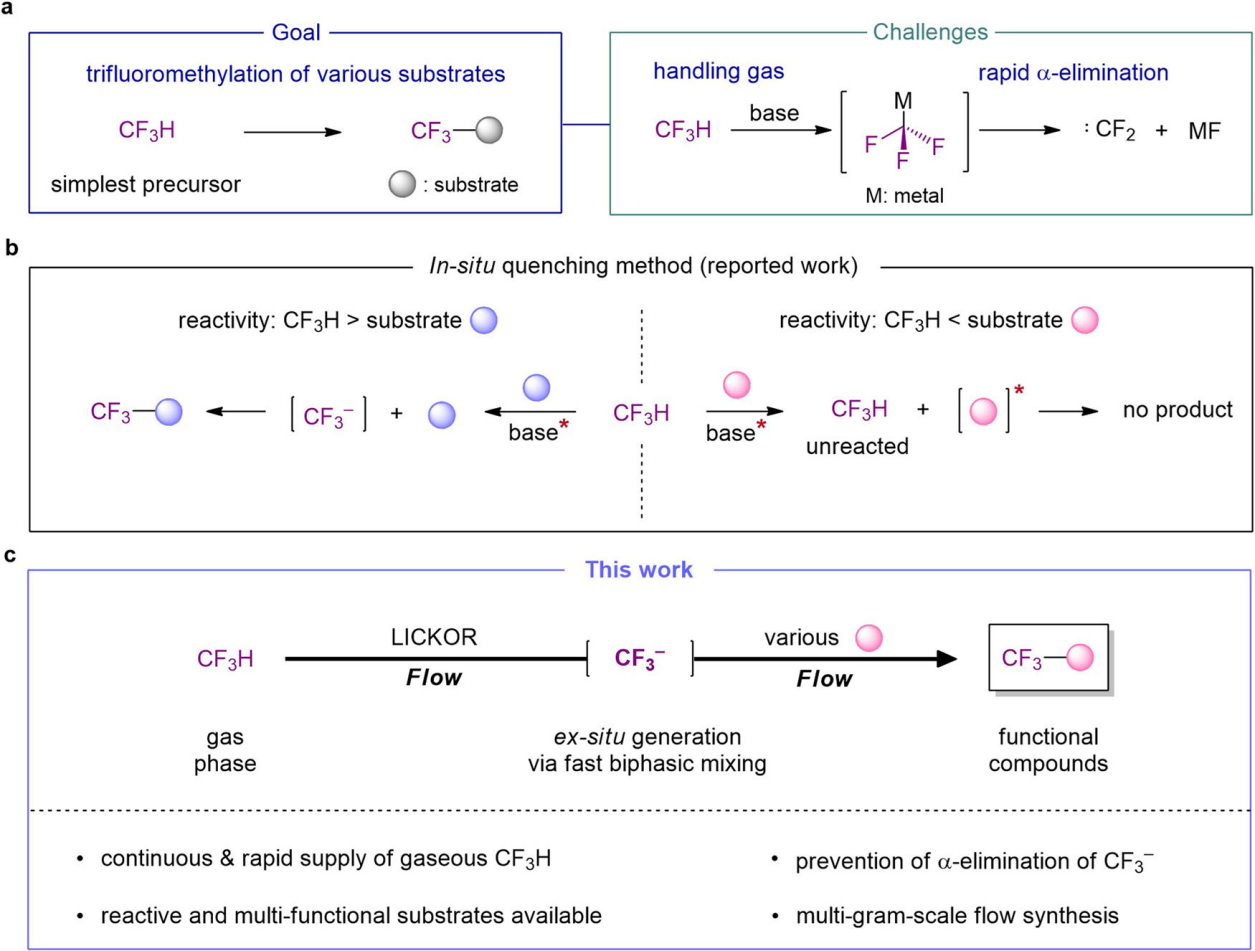
Methods for nucleophilic trifluoromethylation using CF_3_H. **a** A challenge of direct trifluoromethylation using CF_3_^−^ from CF_3_H. **b** An in situ quenching method with less-reactive substrates (reported work) and limitations the of in situ quenching method. **c** An ex situ generation of bare CF_3_^−^ from CF_3_H using LICKOR type of superbase via fast gas-liquid mixing in flow (this work).

The trifluoromethyl (CF_3_) group has been recognized as an important functional group in medicinal chemistry because it can improve the therapeutic efficacy, permeability, metabolic stability of drug molecules, and the binding affinity against proteins^1,2^. Among the extensive synthetic strategies for introducing the CF_3_ group, various synthetic methodologies for nucleophilic trifluoromethylation have been developed^3–6^ but there is no better way to directly use fluoroform (CF_3_H) as the simplest precursor of the CF_3_ group in the viewpoint of atom- and step-economy^7^. Also, the utilization of CF_3_H has attracted from the standpoint of green-sustainable synthesis because CF_3_H is designated as a greenhouse gas (a lifetime of 270 years)^8^. Although pioneer works to directly exploit CF_3_H for the nucleophilic trifluoromethylation were achieved by Prakash’s group and several research groups^9–12^, the synthetic utility of CF_3_H still remains limited because of difficult handling the gaseous CF_3_H and its low reactivity. Above all, the reaction intermediate of the nucleophilic trifluoromethylation, CF_3_ anion (CF_3_^−^) rapidly decomposes into difluorocarbene (:CF_2_) and fluorine ion, as well-documented (Fig. 1a). To avoid these issues on directly using CF_3_H gas with vulnerable decomposition of CF_3_^−^ intermediate even at cryogenic temperatures, the researchers have chosen indirect methods by using the stabilizing additives of CF_3_ anion^13–19^ such as Ruppert-Prakash reagent^20^, or by generating the hemiaminolate adduct [Me_2_NCH(O)CF_3_]K^9^ or coordinate metal cation^21,22^ in DMF or glyme solvent. However, the stabilization method cannot guarantee the use of unstable CF_3_^−^ intermediate, because (1) the stabilized CF_3_- narrowed down their reaction scope due to the less reactivity; (2) its lifetime is still too short to secure the availability. Although CF_3_ anion is also found to be not a transient species but possess a lifetime by using additive, 18-crown-6^23^, this reaction performance cannot be easily utilized in synthetic method due to too low reaction yield and the productivity. To redeem the problems, another indirect method to generate the unstable intermediate in the co-existence of reaction partner (i.e., in situ quenching method or in situ method)^24,25^ is applied as an only alternative to utilize short-lived CF_3_^−^ from CF_3_H prior to the undesired decomposition (Fig. 1b)^12^. Nevertheless, this in situ quenching method approaches could not be completely evitable even in the use of additional reagents for stabilization of CF_3_^−^^9,21,26^ and/or the utilization of flow reaction setup for easy handling the gas with high pressure to prevent gas volatilization^27–31^. This method, however, has fatal problems of not only narrowing down the scope of reaction and the selectivity when the reaction partner is irreconcilable with reaction condition for generating the desired intermediate but also completely depriving the opportunity to explore the nature of a given intermediate for fully understanding a synthetic pathway because the intermediate cannot be solely existed (Fig. 1b)^32^. The large-scale synthesis including gas-liquid reaction is not easily achieved through in situ quenching method as well, because a large quantity of gas should be dissolved in the same solvent with other reactants. Also, most reported works of in situ quenching method required a long reaction time of as few minutes or as many hours as possible even at above room temperature. To overcome the critical limitation and preserve an original strong reactivity of the unstable intermediate, CF_3_^−^ should be formed in the absence of any reaction partner or stabilizing reagent, prior to its following reaction (ex situ quenching method or ex situ method). However, this approach has not been applicable in the generation of CF_3_^−^ from CF_3_H because it is challenging to achieve both fast biphasic mixing of gaseous CF_3_H with liquid deprotonating reagent within the short lifetime of CF_3_^−^ and precise time control for external trapping reaction with generated CF_3_^−^ even using flow-type reactors that is well-known to afford rapid mixing efficacy unachievable in flask^33–35^. We envisaged that this longstanding fundamental problem can be solved by a precise screening of reagents and reaction conditions using a well-designed flow dissolver based on the computer simulation.

Here, we report a strategy to generate and utilize the short-lived CF_3_^−^ intermediate from stable CF_3_H gas under ex situ method using LICKOR type superbase via fast biphasic mixing in precisely customized flow dissolvers (Fig. 1c). The method allows us not only to reveal accurate information on the lifetime and stability of CF_3_^−^ but also to conduct the chemoselective reaction with various substrates by addressing a fundamental obstacle with the in situ method.

## Results and discussion

### Gas-liquid flow devices for rapid biphasic mixing

**Fig. 2:**
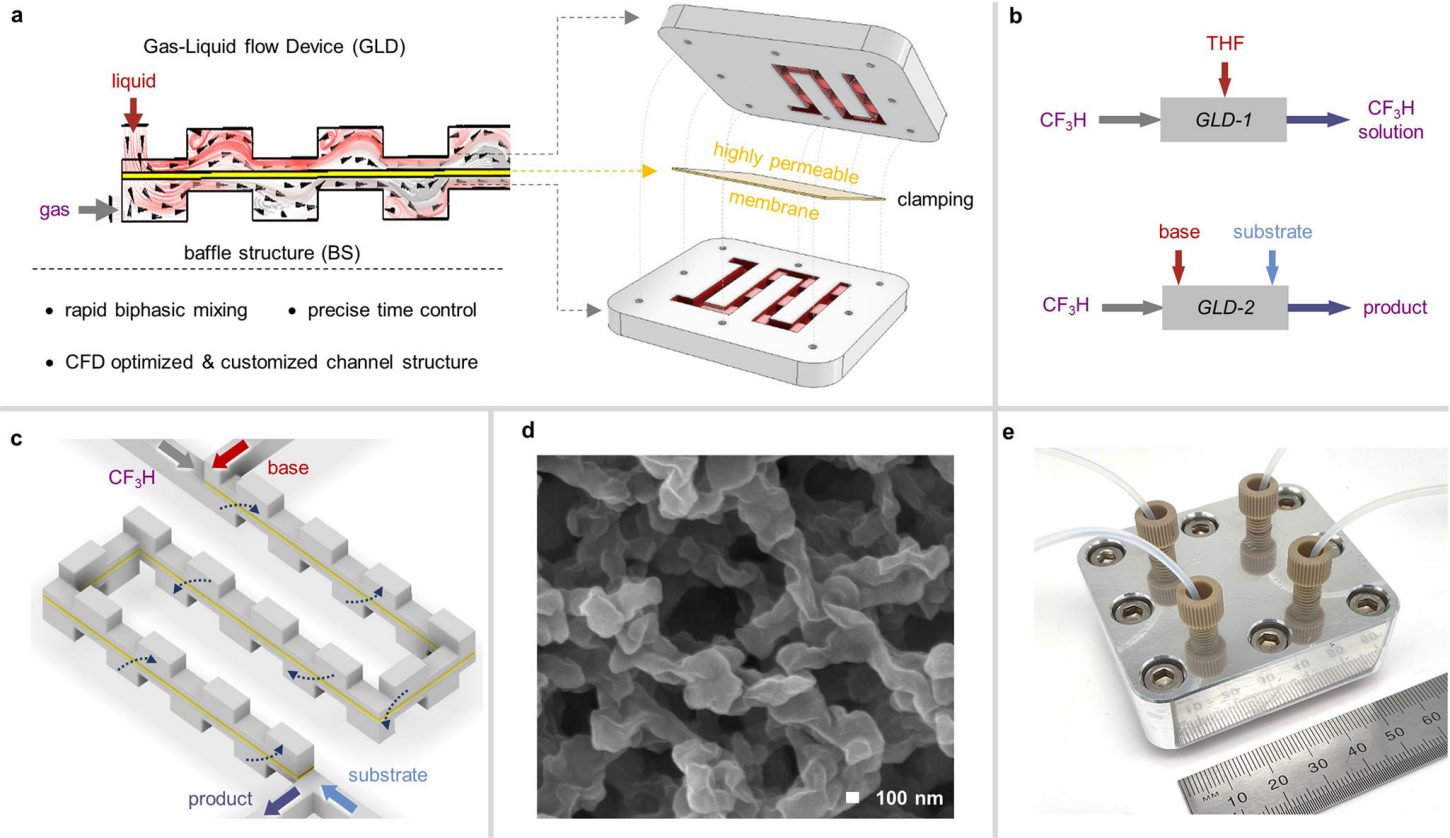
Design, CFD-based optimization, and fabrication of gas-liquid flow device (GLD) including highly permeable membrane and staggered baffle channel. **a** A schematic concept and fabrication of gas-liquid flow device (GLD) via highly permeable membrane and staggered baffle channels. **b** A schematic concept of GLD-1 and GLD-2. **c** A detailed scheme in a fast gas-liquid reaction space in the GLD-2 device with three inlets. **d** An optical SEM image of the 800 nm of pore size of nano-porous membrane (scale bar, 100 nm). **e** An optical image of GLD-2 assembly with in- and outlet tubes.

We initially considered using a simple tubing flow reactor or a tube-in-tube microfluidic reactor well used for gas-liquid flow reactions earlier^36–38^. However, both are not appropriate for rapid gas-liquid biphasic mixing and generation/reaction time control of CF_3_^−^ due to low mixing efficiency, which makes it difficult to precisely control the residence time for CF_3_^−^. Therefore, it was necessary to devise a new flow dissolver that satisfies the purpose to handle the bare CF_3_^−^ at our will. Based on the fact that efficient biphasic mixing for rapid dissolution can be achieved by a large contact area between gas and liquid with a smaller size gas bubble^39^, we conceptually designed various flow channel structured dissolvers composed of highly permeable nano-porous membrane sandwiched between upper and lower channels with staggered baffle structure. The baffle structured channels induced chaotic advection involving rapid distortion of the fluid interface^35^ when the gas-liquid mixture flows over the upper and the lower channels, while the nano-porous membrane in the middle acted as a static mixer and bubble breaker by preventing coalescence of gas segments which must severely reduce the contact area (Fig. 2a)^40,41^. On the basis of computational fluid dynamics (CFD)^42^, these structures were evaluated by monitoring the interfacial contact areas as iso-surface between gas and liquid in the vortex mixing space with excluding dead volume area (see description of definition of contact area between gas and liquid in the Supplementary Information for details). First of all, upon investigating the geometrical effect of baffle structure (see description of BS-1 to BS-7 in the Supplementary Information), the iso-surface was generally increased as the distance of the structure becomes smaller with the increased number of baffles but decreased for the structures smaller than 2.0 mm due to the increased effect of dead volume^43^. In addition, the height of the baffle also largely affected the iso-surface, indicating that the asymmetrically staggered structure in different heights (1000 and 300 μm of BS-4) gave the superior iso-surface to the others (Supplementary Tables 2 and 4). Consecutively, the effect of membrane porosity and thickness on the BS-4 structure was further tuned (see description of PM-1 to PM-6 in the Supplementary Information) to maximize the interfacial contact area between gas and liquid, over the absence of membrane (Supplementary Tables 3 and 5). In general, the thicker membrane, the smaller pore size, and the more decreased overall porosity lowered the iso-surface, presumably due to the decreased permeability of the gas and liquid. Eventually, the numerical simulation results showed that the asymmetrically staggered baffle structure BS-4 (1000 and 300 μm height, 2.0 mm distance), including 50 μm thickness of the membrane (PM-1) with appropriate pore characteristics (77% porosity and 820 nm pore size), is the most suitable to provide the higher iso-surface (0.266 and 0.286 cm^2^) at flow rates of 14.6 ml/min for gas, 3.0 and 7.8 ml/min for two different liquids, respectively. With the simulation results in hand, we carefully considered the choice of material for the fabrication of the flow dissolver. Our experience with diverse materials based microreactors pointed us to stainless steel for the staggered baffle channel patterned plate and perfluorinated polyether (PFPE) for the nano-porous membrane. These materials offer sufficient physical toughness at high flow rates of pressure and low temperature (−95 °C) as well as chemical inertness at a strong base such as organolithiums (Supplementary Fig. 2)^44^. Next, we fabricated two types of gas-liquid flow devices (GLDs) in which the PFPE nano-porous membrane is sandwiched and clamped by two stainless steel plates patterned with the baffle structures. The internal volume for the gas-liquid mixing space can be changed by controlling the length of the staggered baffle channels.

The GLD type-1 (GLD-1) was designed for fast gas-liquid dissolution under the mild condition and stable feeding of the resulting CF_3_H solution to our total flow set-up (Fig. 2b upside and Supplementary Fig. 3). One important point for the generation of CF_3_^−^ using the continuous flow system is the solubility of gaseous CF_3_H into the liquid phase involving reactants. In case CF_3_H is not or partially dissolved prior to its reaction start, it is difficult to interpret the experimental outcome regarding the generation, reaction, or decomposition of CF_3_^−^ in flow. Thus, we first decided to explore reaction conditions by feeding a solvent for the rapid and complete dissolution at −20 °C through the GLD-1, before the direct use of gaseous CF_3_H without the additional solvent. The achievement of rapid gas dissolution is also crucial to minimize the total time of the flow process as well as to simplify the flow set-up for green and sustainable chemistry in terms of safety enhancement and cost-reduction^45–50^. The GLD-1 has a precisely optimized BS-4 structure with a 5 cm long channel and two inlets for liquid THF and gaseous CF_3_H and one outlet for the resulting CF_3_H solution which is supplied for the reaction. We tested the GLD-1 and could successfully know that CF_3_H was dissolved in THF solution over 98% even within 1.3 s at flow rates of 3.0 ml/min for THF and 14.6 ml/min for CF_3_H with a stable feeding of CF_3_H solution (pressure drop: 1.6 kPa). This result showed that GLD-1 has a much superior dissolution rate to the same volume of the general T-junction capillary tubing system. Also, the tendency of the quantitative experimental test in different baffle structures is correctly matched with the CFD simulation result (Supplementary Table 6 and Supplementary Fig. 5).

On the other hand, the GLD type-2 (GLD-2) was fabricated for the fast gas-liquid reaction for direct use of gaseous CF_3_H and liquid reactant with precise time control (Fig. 2b downside). After exploration and optimization of proper reaction conditions for the generation of CF_3_^−^ by our complete set-up, the GLD-2 can be applied to the rapid biphasic reaction with the achievement of a more simplified reaction system. Furthermore, in the viewpoint of an atom- and step-economy, the direct reaction between gaseous CF_3_H and liquid base for metalation is considerably economical. Figure 2c describes the conceptual design for the channel structure, which has the 5 cm length of BS-4 structure providing maximized contact area at flow rates of 7.8 ml/min for a mixture of THF/Et_2_O and 14.6 ml/min for CF_3_H, respectively (pressure drop: 9.1 kPa and Supplementary Table 4). An optical SEM image of nano-porous membrane and an optical image of GLD-2 assembled with inlet and outlet tubes are also shown in Fig. 2d, e, respectively.

### Exploring reaction condition using GLD-1

**Fig. 3:**
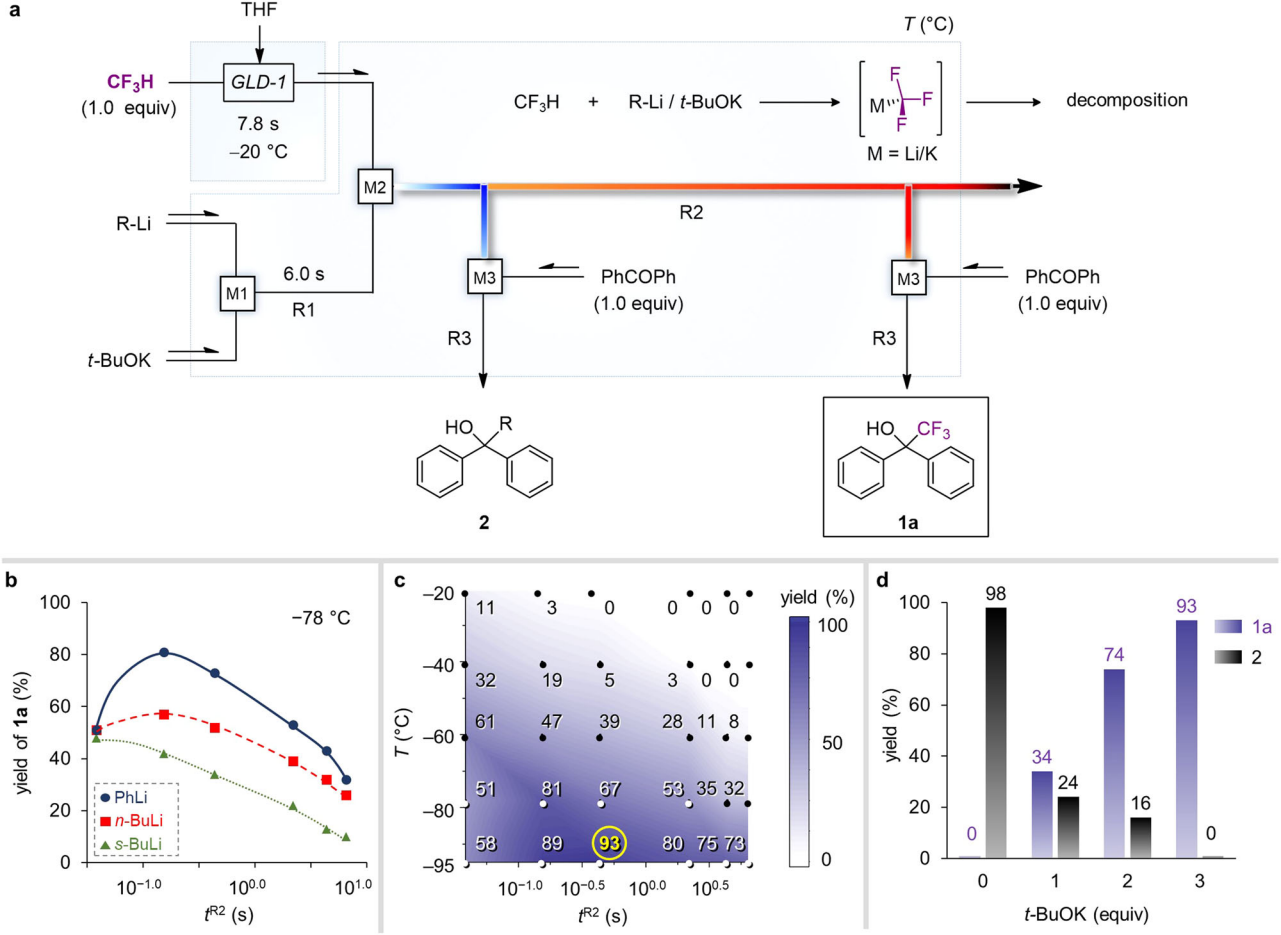
Optimization of reaction condition for ex situ generation of CF_3_^−^ from CF_3_H in flow. **a** A schematic diagram of the flow system. **b** An effect of the type of organolithium on the yield of product **1a**. **c** Effects of temperature and residence time of R2 on the yield of product **1a**. **d** An effect of the amount of *t*-BuOK on the yield of product **1a**.

We began our experimental investigation into exploring the type of bases for rapid metalation of CF_3_H followed by trapping with a stoichiometric amount of benzophenone using GLD-1 and flow reactors (Supplementary Table 7). We found that potassium *tert*-butoxide (*t*-BuOK) and potassium bis(trimethylsilyl)amide (KHMDS) were not efficient to obtain the desired product, 2,2,2-trifluoro-1,1-diphenylmethanol (**1a**) due to their insufficient reactivity within short residence time at a cryogenic temperature, although they were suitable in the in situ method as previously reported^12^. Various organolithium reagents such as lithium diisopropylamide, lithium tetramethylpiperidine, phenyllithium (PhLi), and alkyllithiums (MeLi, *n*-BuLi, *s*-BuLi, and *t*-BuLi) also did not give the product **1a** at all. The latter gave over 71% yield of byproduct **2** via the reaction of *t*-BuLi with benzophenone. These results indicate that organolithium reagents are also inefficient in the metalation of CF_3_H. Subsequently, we attempted to achieve much faster C–H metalation of CF_3_H via a mixture of organolithium and potassium alkoxide called Schlosser’s base or superbase^51^. The reaction was conducted in the flow reaction system consisting of three T-shaped mixers (M1, M2, and M3) and tube reactors (R1, R2, and R3) and GLD-1 (Fig. 3a). Organolithium and *t*-BuOK were mixed in M1 and R1 for 6.0 s of residence time and sequentially reacted with CF_3_H solution in M2 and R2. Three types of organolithiums (PhLi, *n*-BuLi, and *s*-BuLi) with 3 equivalents of *t*-BuOK were used and residence time in R2 (*t*^R2^) was changed from 0.04 to 6.5 s by changing the diameter and length of the R2 at −78 °C (Fig. 3b and Supplementary Table 8). Surprisingly, these combinations allowed obtaining the desired product **1a**. The less-reactive organolithium gave the better yield of the product **1a** in the order of PhLi > *n*-BuLi > *s*-BuLi, probably because of the competitive side reaction of superbase with THF solvent^52^ or nucleophilic attack of organolithium to CF_3_H^53^. We obtained product **1a** in 81% yield using PhLi with *t*-BuOK when *t*^R2^ was 0.15 s at −78 °C, but a small amount of byproduct **2** was detected (4%), which indicates that CF_3_^−^ was not completely generated in R2. For better yield without the formation of byproduct **2**, a variety of reaction conditions were explored (*T* = −20 °C to −95 °C, *t*^R2^ = 0.04–6.5 s) to obtain a contour plot (Fig. 3c), which gave exact information on the stability and the lifetime of CF_3_^−^ in various temperatures. The contour plot divulged that the yield of **1a** was not sufficient in the range of short residence time below 0.15 s for *t*^R2^ at −78 and −95 °C due to incomplete metalation of CF_3_H and CF_3_^−^ has an short lifetime which is largely sensitive to the temperature. We eventually found the proper time and cryogenic zone for the existence of bare CF_3_^−^ in the flow reactor at 0.4 s for *t*^R2^ under −95 °C, which gave an opportunity to solely utilize CF_3_^−^ in absence of the stabilizer and the reaction partner. Under the optimized condition, product **1a** was obtained in 93% yield without the detectable byproduct. Then, the effect of the amount of *t*-BuOK on the yield of product **1a** was investigated (Fig. 3d). The product **1a** was gradually increased from 0 to 93% yield and the byproduct **2** was oppositely decreased from 98 to 0% with increasing the amount of *t*-BuOK, probably because the excess amount of *t*-BuOK provide a solubilizing adduct of superbase with stabilization, which has a positive effect on the reactivity^54^.

### Direct use of gaseous CF_3_H for ex situ reaction using GLD-2

Based on the information obtained using the flow system involving GLD-1, we next utilized the integrated device, GLD-2 that enabled a direct mixing of CF_3_H and superbase, and following reactions. In the GLD-2, product **1a** was obtained in 66% yield with 28% of the byproduct **2**. To accomplish the rapid metalation, the equivalent of the PhLi was slightly increased to 1.3 keeping the ratio of PhLi and *t*-BuOK (1:3), which raised the yield of **1a** to 84% without any byproduct **2**. This result indicates that the generation of CF_3_^−^ through direct biphasic reaction and subsequent bimolecular trapping reaction with benzophenone was successfully accomplished in the GLD-2 under cryogenic temperature.

**Fig. 4:**
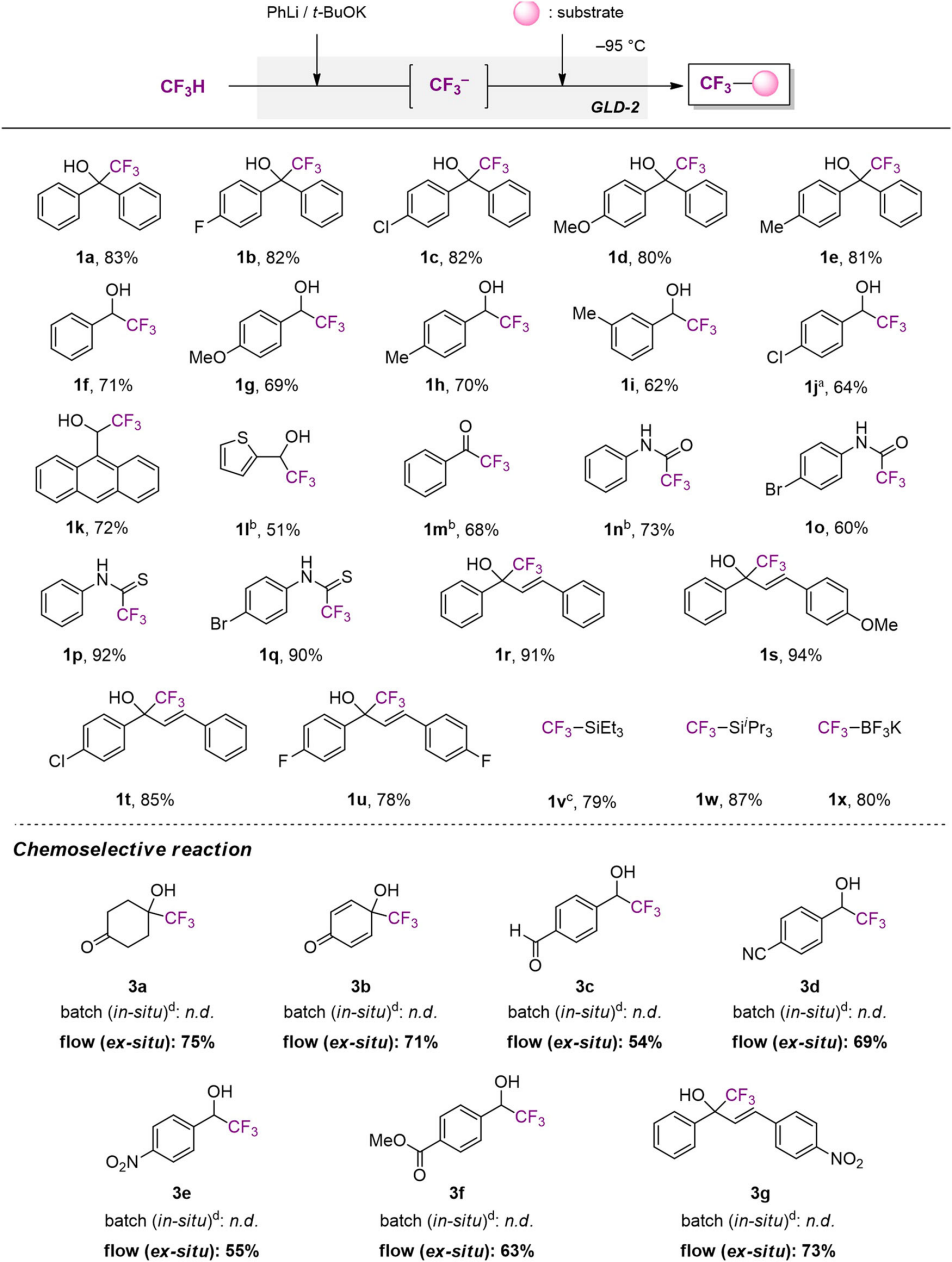
The reaction of bare CF_3_^−^ with various electrophilic substrates in GLD-2. Isolated yield. ^a^The resulting CF_3_^−^ solution was reacted with an electrophile at –50 °C. ^b^The CF_3_^−^ solution was reacted with an electrophile at −78 °C. ^c^TESCl (2 equiv) was used. ^d^The yield was determined by ^1^H NMR spectroscopy using an internal standard.

The reactions were conducted using various electrophilic substrates such as ketones, aldehydes, an ester, isocyanates, isothiocyanates, and chalcones in the GLD-2 (Fig. 4). We obtained the desired products (**1a**–**1j**) through the reactions with benzophenones and benzaldehydes bearing electron-donating (-OMe and -Me) or withdrawing group (-F, and -Cl) in good yields of 62–83%. The reactions with 9-anthracenecarboxaldehyde and 2-thiophenecarboxaldehyde gave the desired products **1k** and **1l** in 72% and 51% yield, respectively. The reaction of methyl benzoate represented the remarkable advantage of our system by affording the corresponding product **1m** in 68% yield without a significant loss caused by the sequential addition to the acyl group or unwanted esterification by *t*-BuOK^55^. For this reason, the yield was more than twice as high as the reported in situ quenching method^12^. Aryl compounds bearing isocyanate and isothiocyanate group effectively reacted as well with flow-generated CF_3_^−^ to give the corresponding products (**1n**–**1q**) in 60–92% good yields. We also accomplished the reactions with chalcones to give the desired products (**1r**–**1u**) in 78–94% yields. We conducted the silylation for the synthesis of Ruppert-Prakash-type reagents as well. Pleasingly, ex situ generated CF_3_^−^ was successfully reacted with triethylsilyl chloride and triisopropylsilyl chloride and gave product **1v** in 79% and **1w** in 87% yield, respectively. Potassium (trifluoromethyl)trifluoroborate (CF_3_BF_3_K) **1x** was also prepared by borylation with trimethoxyborane, B(OMe)_3_ followed by sequential reaction with potassium bifluoride in 80% yield.

We next sought to strengthen the powerful advantage of our synthetic methodology by the accomplishment of chemoselective reactions with substrates bearing two electron-withdrawing groups. The selective nucleophilic addition with electrophiles bearing two same functional groups (acyl or formyl group) such as 1,4-cyclohexanedione, benzoquinone, and terephthalaldehyde gave the corresponding products (**3a**–**3c**) in 54–75% yields without over-reacted byproduct. We also conducted the reaction with benzaldehyde bearing an additional functional group (cyano, nitro, or methoxycarbonyl group) and chalcone bearing a nitro group, which provided the desired products **3d**–**3g** over 55% yield. It is highly noteworthy that we cannot obtain the desired products at all by the in situ quenching method in the flask. The results clearly show that our methodology is highly valuable for accomplishing protecting-group-free synthesis of multi-functionalized molecules.

### Continuous multi-gram-scale synthesis using one-flow system

**Fig. 5:**
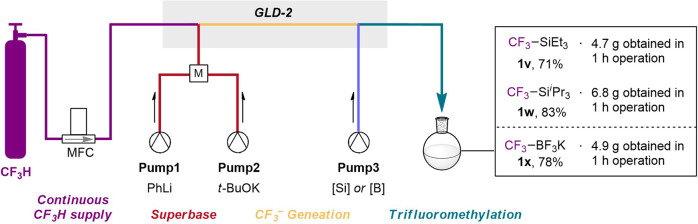
The multi-gram-scale synthesis. One-flow multi-gram synthesis using an integrated flow system involving GLD-2.

Lastly, we carried out the multi-gram-scale synthesis of three important chemical reagents which are used for nucleophilic trifluoromethylation using the GLD-2 device (Fig. 5). An integrated flow system to achieve one-flow CF_3_H supply, the formation of superbase, CF_3_^−^ generation, and trifluoromethylation of silanes or borane continuously operated for 1 h, to result in the successful preparation of target compounds, trifluoromethyl silanes (**1v** and **1w**), and CF_3_BF_3_K (**1x**) in good isolated yields and remarkable productivity (71–83%, 4.7–6.8 g/h). Although the GLD-2 is quite small enough to fit on one hand, a high total flow rate (13.8 ml/min) enables large-scale production, unlike the ordinary microfluidic system.

## Methods

### Reaction with various electrophiles in flow

A solution of PhLi (0.13 M in Et_2_O, 6.0 ml/min) and a solution of *t*-BuOK (1.3 M in THF, 1.8 ml/min) were introduced to M (inner tube *ϕ* = 250 μm) by syringe pumps and passed through tube reactor (*ϕ* = 1000 μm, *L* = 100 cm). The resulting solution and CF_3_H (flow rate: 14.6 ml/min) were introduced to two inlets of the GLD-2 device and CF_3_H was introduced by MFC. The resulting solution was passed through a nano-porous membrane sandwiched by a staggered baffle structure and was mixed with a solution of various electrophiles (0.3 M in THF, 3.0 ml/min). The resulting solution was passed through tube reactor (*ϕ* = 1000 μm, *L* = 50 cm). After a steady-state was reached (90 s), the product solution was collected for 30 s while being quenched with saturated NH_4_Cl aqueous solution (2 ml). For the isolation of the product, the resulting solution was extracted with Et_2_O (3 × 10 ml), then the organic phase was extracted with brine (10 ml), dried over Na_2_SO_4_ and concentrated. The crude product was purified by column chromatography to give the desired product.

## Supplementary information

Supplemenatry Information

Peer Review File

## Data Availability

All other data that support the findings of this study, which include experimental procedures and compound characterization, are available within the paper and its Supplementary Information.

## References

[1] Muller K, Faeh C, Diederich F (2007). Fluorine in pharmaceuticals: looking beyond intuition. Science.

[2] Meanwell NA (2018). Fluorine and fluorinated motifs in the design and application of bioisosteres for drug design. J. Med. Chem..

[3] Liu X, Xu C, Wang M, Liu Q (2015). Trifluoromethyltrimethylsilane: nucleophilic trifluoromethylation and beyond. Chem. Rev..

[4] Okusu S, Hirano K, Yasuda Y, Tokunaga E, Shibata N (2016). Flow trifluoromethylation of carbonyl compounds by Ruppert–Prakash reagent and its application for pharmaceuticals, efavirenz and HSD-016. RSC Adv..

[5] Geri JB, Szymczak NK (2017). Recyclable trifluoromethylation reagents from fluoroform. J. Am. Chem. Soc..

[6] Mitobe K (2018). Preparation and reactions of CF3‐containing phthalides. Eur. J. Org. Chem..

[7] Trost BM (1991). The atom economy—a search for synthetic efficiency. Science.

[8] Han W, Li Y, Tang H, Liu H (2012). Treatment of the potent greenhouse gas, CHF3—an overview. J. Fluor. Chem..

[9] Shono T, Ishifune M, Okada T, Kashimura S (1991). Electroorganic chemistry. 130. A novel trifluoromethylation of aldehydes and ketones promoted by an electrogenerated base. J. Org. Chem..

[10] Folléas B, Marek I, Normant J-F, Jalmes LS (1998). Fluoroform: an efficient precursor for the trifluoromethylation of aldehydes. Tetrahedron Lett..

[11] Large S, Roques N, Langlois BR (2000). Nucleophilic trifluoromethylation of carbonyl compounds and disulfides with trifluoromethane and silicon-containing bases. J. Org. Chem..

[12] Prakash GS, Jog PV, Batamack PT, Olah GA (2012). Taming of fluoroform: direct nucleophilic trifluoromethylation of Si, B, S, and C centers. Science.

[13] Shono T, Ishifune M, Okada T, Kashimura S (1991). Electroorganic chemistry. 130. A novel trifluoromethylation of aldehydes and ketones promoted by an electrogenerated base. J. Org. Chem..

[14] Kawai H, Yuan Z, Tokunaga E, Shibata N (2013). A sterically demanding organo-superbase avoids decomposition of a naked trifluoromethyl carbanion directly generated from fluoroform. Org. Biomol. Chem..

[15] Okusu S, Hirano K, Tokunaga E, Shibata N (2015). Organocatalyzed trifluoromethylation of ketones and sulfonyl fluorides by fluoroform under a superbase system. ChemistryOpen.

[16] Punna N (2018). Stereodivergent trifluoromethylation of N-sulfinylimines by fluoroform with either organic-superbase or organometallic-base. Chem. Commun..

[17] Lishchynskyi A (2015). The trifluoromethyl anion. Angew. Chem..

[18] Miloserdov FM (2017). The trifluoromethyl anion: evidence for [K (crypt‐222)]+. Helv. Chim. Acta.

[19] Harlow RL (2018). On the structure of [K (crypt‐222)]+. Helv. Chim. Acta.

[20] Prakash GS, Krishnamurti R, Olah GA (1989). Synthetic methods and reactions. 141. Fluoride-induced trifluoromethylation of carbonyl compounds with trifluoromethyltrimethylsilane (TMS-CF3). A trifluoromethide equivalent. J. Am. Chem. Soc..

[21] Saito T, Wang J, Tokunaga E, Tsuzuki S, Shibata N (2018). Direct nucleophilic trifluoromethylation of carbonyl compounds by potent greenhouse gas, fluoroform: Improving the reactivity of anionoid trifluoromethyl species in glymes. Sci. Rep..

[22] Fujihira Y (2021). Synthesis of trifluoromethyl ketones by nucleophilic trifluoromethylation of esters under a fluoroform/KHMDS/triglyme system. Beilstein J. Org. Chem..

[23] Prakash GS (2014). Long‐lived trifluoromethanide anion: a key intermediate in nucleophilic trifluoromethylations. Angew. Chem. Int. Ed..

[24] Pace V (2017). Efficient access to all‐carbon quaternary and tertiary α‐functionalized homoallyl‐type aldehydes from ketones. Angew. Chem. Int. Ed..

[25] Parisi G (2017). Exploiting a “Beast” in carbenoid chemistry: development of a straightforward direct nucleophilic fluoromethylation strategy. J. Am. Chem. Soc..

[26] Zanardi A, Novikov MA, Martin E, Benet-Buchholz J, Grushin VV (2011). Direct cupration of fluoroform. J. Am. Chem. Soc..

[27] Fu WC, MacQueen PM, Jamison TF (2021). Continuous flow strategies for using fluorinated greenhouse gases in fluoroalkylations. Chem. Soc. Rev..

[28] Köckinger M (2018). Utilization of fluoroform for difluoromethylation in continuous flow: a concise synthesis of α-difluoromethyl-amino acids. Green. Chem..

[29] Musio B, Gala E, Ley SV (2018). Real-time spectroscopic analysis enabling quantitative and safe consumption of fluoroform during nucleophilic trifluoromethylation in flow. ACS Sustain. Chem. Eng..

[30] Hirano K, Gondo S, Punna N, Tokunaga E, Shibata N (2019). Gas/liquid‐phase micro‐flow trifluoromethylation using fluoroform: trifluoromethylation of aldehydes, ketones, chalcones, and N‐sulfinylimines. ChemistryOpen.

[31] Ono M (2021). Pentafluoroethylation of carbonyl compounds using HFC-125 in a flow microreactor system. J. Org. Chem..

[32] Colella M (2020). Fluoro‐substituted methyllithium chemistry: external quenching method using flow microreactors. Angew. Chem. Int. Ed..

[33] Kim H, Nagaki A, Yoshida J-I (2011). A flow-microreactor approach to protecting-group-free synthesis using organolithium compounds. Nat. Commun..

[34] Yoshida J-I, Takahashi Y, Nagaki A (2013). Flash chemistry: flow chemistry that cannot be done in batch. Chem. Commun..

[35] Kim H (2016). Submillisecond organic synthesis: outpacing fries rearrangement through microfluidic rapid mixing. Science.

[36] Yen BK, Günther A, Schmidt MA, Jensen KF, Bawendi MG (2005). A microfabricated gas–liquid segmented flow reactor for high‐temperature synthesis: the case of CdSe quantum dots. Angew. Chem. Int. Ed..

[37] Han S, Kashfipour MA, Ramezani M, Abolhasani M (2020). Accelerating gas–liquid chemical reactions in flow. Chem. Commun..

[38] Yang L, Jensen KF (2013). Mass transport and reactions in the tube-in-tube reactor. Org. Process Res. Dev..

[39] Wang Z (2020). Effects of bubble size on the gas–liquid mass transfer of bubble swarms with Sauter mean diameters of 0.38–4.88 mm in a co‐current upflow bubble column. J. Chem. Technol. Biotechnol..

[40] Femmer T, Eggersdorfer ML, Kuehne AJ, Wessling M (2015). Efficient gas–liquid contact using microfluidic membrane devices with staggered herringbone mixers. Lab Chip.

[41] Ahmed AKA (2018). Generation of nanobubbles by ceramic membrane filters: the dependence of bubble size and zeta potential on surface coating, pore size and injected gas pressure. Chemosphere.

[42] Ataki A, Bart HJ (2006). Experimental and CFD simulation study for the wetting of a structured packing element with liquids. Chem. Eng. Technol..

[43] Zhang J, Tejada-Martínez AE, Zhang Q (2014). Developments in computational fluid dynamics-based modeling for disinfection technologies over the last two decades: a review. Environ. Model. Softw..

[44] Kim J-O (2014). A monolithic and flexible fluoropolymer film microreactor for organic synthesis applications. Lab Chip.

[45] Ley SV, Fitzpatrick DE, Myers RM, Battilocchio C, Ingham RJ (2015). Machine‐assisted organic synthesis. Angew. Chem. Int. Ed..

[46] Tsubogo T, Oyamada H, Kobayashi S (2015). Multistep continuous-flow synthesis of (R)-and (S)-rolipram using heterogeneous catalysts. Nature.

[47] Cambie D, Bottecchia C, Straathof NJ, Hessel V, Noel T (2016). Applications of continuous-flow photochemistry in organic synthesis, material science, and water treatment. Chem. Rev..

[48] Plutschack MB, Pieber BU, Gilmore K, Seeberger PH (2017). The hitchhiker’s guide to flow chemistry. Chem. Rev..

[49] Coley CW (2019). A robotic platform for flow synthesis of organic compounds informed by AI planning. Science.

[50] Dallinger D, Gutmann B, Kappe CO (2020). The concept of chemical generators: on-site on-demand production of hazardous reagents in continuous flow. Acc. Chem. Res..

[51] Schlosser M, Jung HC, Takagishi S (1990). Selective mono-or dimetalation of arenes by means of superbasic reagents. Tetrahedron.

[52] Clayden J, Yasin SA (2002). Pathways for decomposition of THF by organolithiums: the role of HMPA. N. J. Chem..

[53] Maruyama K, Saito D, Mikami K (2018). (Sila) Difluoromethylation of Fluorenyllithium with CF3H and CF3TMS. SynOpen.

[54] Klett J (2021). Structural motifs of alkali metal superbases in non‐coordinating solvents. Chem. Eur. J..

[55] Yang HS, Macha L, Ha H-J, Yang JW (2021). Functionalisation of esters via 1, 3-chelation using NaO t Bu: mechanistic investigations and synthetic applications. Org. Chem. Front..

