# Supplementary material for: Ex-situ generation and synthetic utilization of bare trifluoromethyl anion in flow via rapid biphasic mixing
Source: Nat Commun. 2023 Mar 3;14:1231. doi: 10.1038/s41467-022-35611-9 (PMC9984407; doi:10.1038/s41467-022-35611-9)
Supplement: Supplementary file 1 — Supplemenatry Information [file 41467_2022_35611_MOESM1_ESM.pdf]

Supplementary Information

Ex-situ generation and synthetic utilization of bare trifluoromethyl anion in flow via rapid biphasic mixing

Hyune-Jea Lee[1,2,†], Jeong-Un Joo[3,†], Se-Jun Yim[3], Dong-Pyo Kim[3*], Heejin Kim[1*]

[1]Department of Chemistry, College of Science, Korea University, 145 Anam-ro, Seongbuk-gu, Seoul 02841, Republic of Korea.

[2]Samsung Advanced Institute of Technology, 130 Samsung-ro, Yeongtong-gu, Suwon-si, Gyeonggi-do 16678, Republic of Korea.

[3]Department of Chemical Engineering, Pohang University of Science and Technology, 77 Cheongam-ro, Nam-gu, Pohang-si, Gyeongsangbuk-do 37673, Republic of Korea.

[†]These authors contributed equally to this work.

*Corresponding author. Email: dpkim@postech.ac.kr (D.-P.K.); heejinkim@korea.ac.kr (H.K.)

Table of Contents

I. Supplementary Method

1. General information
2. Computational fluidic dynamics simulation of gas-liquid contact area in channel structures
3. Fabrication of gas-liquid flow device (GLD)
4. Quantitative analysis of CF_3H solution in THF through GLD-1
5. Generation of bare CF_3 anion from CF_3H
6. Reaction with various electrophilic substrates
7. Continuous flow mass production through GLD-2
8. ^{1}H, ^{13}C, ^{19}F, and ^{11}B NMR Spectra

II. Supplementary References

I. Supplementary Method

1. General information

Gas chromatograph (GC) analysis was performed on a SHIMADZU GC-2030 equipped with a flame ionization detector using a fused silica capillary column (column, CBPI; 0.25 mm×25 m; heating at a rate of 10 °C/min from initial 50 °C conditions). Nuclear magnetic resonance (NMR) spectra were recorded with Bruker Avancd III (^{1}H 500 MHz, ^{13}C 125 MHz, ^{19}F 470 MHz, ^{11}B 160 MHz). ^{1}H and ^{13}C chemical shifts are recorded in ppm downfield of tetramethylsilane and referenced to residual solvent peak ($CHCl_3$=7.26) unless otherwise noted. Multiplicities are reported using the following abbreviation: s = singlet, d = doublet, t = triplet, q = quartet, quint = quintet, sext = sextet, sept = septet, m = multiplet, br = broad resonance. Electron ionization (EI) mass spectra were recorded on a JEOL JMS 700 spectrometer in the Korea Basic Science Institute (Daegu). Scanning electron microscope (SEM) micrographs of the samples were taken using a SEM (JEOL JSM-7800F) at an accelerating voltage of 5.0 keV. Supercritical drying for SEM analysis was performed on a Tousimis™ Samdri® PVT-3D. Quantitative analysis of CF_3H in THF by an online gas chromatograph was performed on a Varian GC-450 equipped with a flame ionization detector using a BR-5M column; 0.25 mm×30 m; heating at a rate of 30 °C/min from initial 45 °C conditions). Unless otherwise noted all commercial materials were used without further purification. Tetrahydrofuran (THF, anhydrous, ≥99%, inhibitor-free) and diethyl ether (Et_2O, ≥99.9%, inhibitor-free) were purchased from Sigma-Aldrich and used without further purification. All organolithium reagents and potassium bis(trimethylsilyl)amide solution were purchased from Sigma-Aldrich and Kanto Chemical Co., Inc.. Potassium tert-butoxide solution (1.0 M in THF) was purchased from TCI. All electrophiles were purchased from Sigma-Aldrich, TCI, or Alfa Aesar and used without further purification.

Stainless steel (SUS316) tube reactors with inner diameters of 0.25 mm and 1 mm were purchased from GL Science and were cut into appropriate lengths. Stainless steel (SUS304) T-shaped micromixers with inner diameters of 250 and 500 μm were manufactured by YMC Co. and Valco Instruments Co. Inc. The micromixers and tube reactors were connected with stainless steel fittings (GL Science, 1/16" OUW) to construct the flow reaction system. The flow reaction system was dipped in a water bath or cooling bath to control the temperature. The reagents were continuously injected into the flow reaction system using syringe pumps (KD Scientific, Legato 200, and Legato 100), equipped with gas-tight syringes (50 mL, inner diameter: 27.6 mm) purchased from SGE Analytical Science. Constant pressure dual head pump (TELEDYNE TECHNOLOGIES, CP Class) and dual pump (FLOM, KP-22) were used for mass-production experiments. The gas flow was controlled by a mass flow controller from ISVT (ISVT500-51CSV) after calibrating for CF_3H. After a steady-state was reached (90 s), the product solution was collected for 30 s unless otherwise noted.

2. Computational fluidic dynamics simulation of gas-liquid contact area in channel structures

Mathematical formulation and numerical details. The fluid flow inside a microchannel can be described by the Navier-stokes equation together with multiphase fluids, the volume of fluid (VOF) equation, and a mass-conservation equation. Assuming steady-state, the governing equation for fluid flow can be simplified as follows:

$$\rho \mathbf{v} \cdot \nabla \mathbf{v} = -\nabla p + \mu \nabla^2 \mathbf{v} + \rho \mathbf{g} \qquad \text{(1): Navier-Stokes equation}$$

$$\nabla \cdot \mathbf{v} = 0 \qquad \text{(2): Mass conversion equation}$$

Where ρ [kg·m^{-3}] is the fluid density, $\mathbf{v}$ [m·s^{-1}] is the fluid linear velocity, p [Pa] is the pressure, μ [kg·m^{-1}·s^{-1}] is the fluid dynamic viscosity and $\mathbf{g}$ is the gravitational acceleration (9.8 m·s^{-2}). The governing equations were solved with appropriate boundary conditions (no-slip boundary condition on channel walls, the mass flow rate for inlets, and outflow conditions for outlets, zero normal gradients for all flow variables except pressure). The equations were discretized using a finite-volume method and commercial numerical software FLUENT 2020 R1 (ANSYS, INC.) was used for the numerical simulation. The geometry of channel structure was modeled in the commercially available AutoDesk Inventor program and meshing of the model was done in ANSYS fluent meshing module. The structured meshes were generated with hexahedral and tetrahedral elements with a typical size of 10–100 μm. We selected the Shear-Stress Transport (SST) k-ω standard turbulence model which is developed by merging developed by merging both k-ε turbulence and k-ω turbulence model[1]. The k-ε turbulence model is widely utilized for calculating turbulence kinetic energy but cannot accurately explain the fluid near the wall and the reverse pressure gradient flow. On the other hand, the k-ε model can interpret the fluid near the wall. In our work, small dimension of flow channel with nano-porous membrane were used and they gave boundary layer flow delay due to a reverse pressure gradient because turbulence flow is not balanced. In addition, the VOF model was used to investigate the interface between gas and liquid phases. The physical properties (density and viscosity) of tetrahydrofuran (928 kg/m^3, 0.000564 kg/m/s at –20 °C and 980.9 kg/m^3, 0.00185 kg/m/s at –95 °C), diethyl ether (809.2 kg/m^3, 0.000845 kg/m/s at –95 °C) and CF_3H (3.372 kg/m^3, 1.27 ×10^{-5} kg/m/s at –20 °C and 4.4857 kg/m^3, 9.68 ×10^{-6} kg/m/s at –95 °C) were used for fluid properties.

Membrane model setup for Computational Fluid Dynamics. The fluid flow inside a porous media can be described by the Ergun equation[1,2], which gives the total pressure drop through a porous media from viscous and inertial resistance to flow. The Ergun equation is given as

$$dP/dl = R_v \mu \mathbf{v} + \frac{1}{2} R_i \rho \mathbf{v}^2 \qquad (3)$$

Where P is pressure, l is thickness of membrane, R_v is viscous resistance, R_i is inertial resistance, μ is the fluid dynamic viscosity, $\mathbf{v}$ is the fluid linear velocity and ρ is the fluid density. The R_v and R_i is given as

$$R_v = 150(1-e)^2/D_p^2 e^3 \tag{4}$$

$$R_i = 3.5(1-e)/D_p e^3 \tag{5}$$

$$D_p = 3d_h(1-e)/2e \tag{6}$$

Where e is the porosity of the medium, d_h is the mean diameter of pore size. In the Ergun equation, the first term represents the viscous loss (proportional to velocity) and the second term shows the inertial loss (proportional to velocity squared).

Definition of contact area between gas and liquid. To quantify the contact area of gas and liquid fluids (here carrier solvent THF or THF/Et_2O, gaseous reagent CF_3H) depending on the channel geometry, the sum of the area corresponding to iso-surface at 0.5 of volume fraction excluding the dead volume was calculated because the contact area cannot be directly obtained from FLUENT. The dead volume was determined according to the region with the flow velocities less than 5% of the maximum velocity and highlighted to dark blue color in visualized CFD data as shown in Table S1 to Table S5[3].

Determination of gas flow rate. The flow rate of CF_3H can be determined by Van der Waals equation to inject the accurate amount of gas. The Van der Waals equation is given as

$$[P + a(n/V)^2](V/n - b) = RT \tag{7}$$

Where P is pressure, constant a is Van der Waals constant to provide correction for the intermolecular forces and give 5.378 bar·L^2·mol^{-2} for CF_3H, n is moles of gas, V is volume of gas, constant b is Van der Waals constant to adjust for the volume occupied by the gas particles and give 0.06403 L·mol^{-1}, R is gas constant as 0.08314 L·bar·K^{-1}·mol^{-1} and T is temperature. Because the injection system is located at atmospheric condition, T was calculated as 298 K and pressure was controlled as 4.78 MPa by pressure regulator.

Contact area of gas-liquid fluids in various channel geometries. In order to rapidly mixing the CF_3H gas with THF solvent and to control the short-lived CF_3 anion, it is desirable to devise the flow device for gas dissolution with superior contact area between gas and liquid for reducing the dissolution time. A simulation was carried out to elucidate the contact area of biphasic fluid in the various nano-porous

membrane characteristics and the various channel geometries for the dissolution space design of the flow channel. Because contact area is naturally increased by increasing the channel length and width, the length and the width of flow channel were set to 2 cm and 1000 μm, respectively. The representative nano-porous membrane and channel geometries were varied as shown in Table S2 to S5 after screening of the contact area from the iso-surface at 0.5 of volume fraction; In case of channel geometries of baffle structure (BS): BS-1 is a nano-porous membrane sandwiched by 1000 μm height of rectangular cross-sectional channels; BS-2 to BS-5 are nano-porous structures sandwiched by 1000 μm and 300 μm height of rectangular cross-sectional channels with a different distance of staggered baffle structures; BS-6 has 1000 μm and 100 μm height of rectangular cross-sectional channel with 2 mm of staggered baffle structure; BS-7 has 1000 μm and 500 μm height of rectangular cross-sectional channel with 2 mm of staggered baffle structure. In case of nano-porous membrane (PM): PM-1 to PM-6 are varied from 77–60% of overall porosity (820–310 nm of pore size) and 50–150 μm of thickness.

Initially, we optimized the flow condition by varying the flow rate and injected direction of the gas at the fixed concentration of CF_3H solution (0.2 M in THF for GLD-1; 0.08 M in THF/Et_2O for GLD-2) as shown in Table S1. As a result, we chose the flow rate conditions to give high iso-surface area; for GLD-1, 3.0 mL/min for THF & 14.6 mL/min for CF_3H at –20 °C; for GLD-2, 7.8 mL/min for THF/Et_2O & 14.6 mL/min for CF_3H at –95 °C, respectively. Next, we tested the contact area of various baffle geometries (BS-1 to BS-7) by embedding with the typical membrane (PM-1) under optimized flow conditions. The resulting iso-surface at 0.5 of volume fraction at different geometry and flow condition are summarized in Table S2 and S4. According to the contact area, we optimized channel geometry for rapid gas-liquid dissolution and time-dependent intermediate control. As a result, we chose BS-4 (2.0 mm of baffle distance with 1000 μm and 300 μm height of rectangular cross-sectional channel). Next, we tested the membrane PM-1 to PM-6 in baffle structure BS-4 as shown in Table S3 and S5. As a result, we chose PM-1 (77% of overall porosity, 820 nm of the pore size and 50 μm of thickness). Finally, BS-4 geometry with PM-1 membrane was the most suitable to devise a GLD-1 for rapid gas-liquid dissolution and a GLD-2 for fast and direct gas-liquid reaction because it shows the highest contact area among channel geometries.

Supplementary Table 1. Flow-dependent iso-surface of simple membrane-embedded dual-channel without baffle structure (BS-1) at various flow rates of gas-liquid.

GLD-1	Flow rate (mL/min)<br>(liquid-gas)					
	4-19.5		**3-14.6**		3-14.6	
BS-1	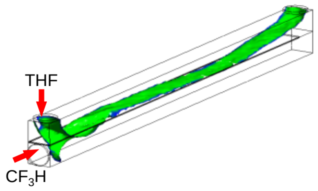		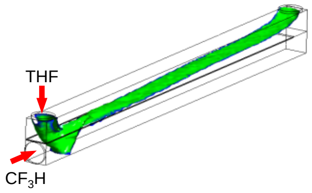		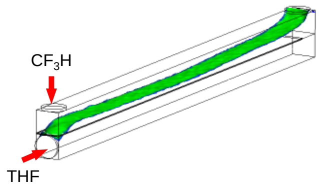	
	Iso-surface (cm^2)	0.206	**Iso-surface (cm^2)**	**0.222**	Iso-surface (cm^2)	0.176
	2-9.6		1-4.7			
	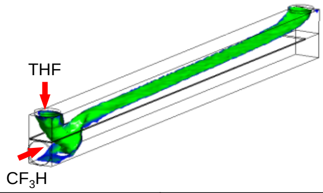		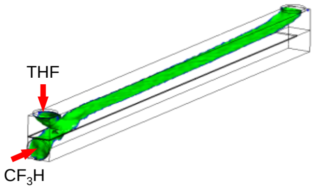			
	Iso-surface (cm^2)	0.217	Iso-surface (cm^2)	0.213		
GLD-2	Flow rate (mL/min)<br>(liquid-gas)					
	9-17.2		**7.8-14.6**		7.8-14.6	
BS-1	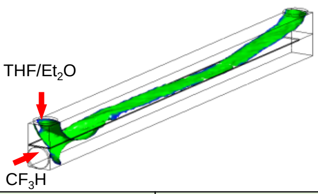		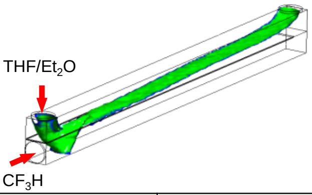		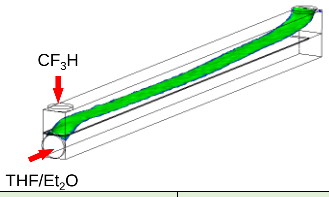	
	Iso-surface (cm^2)	0.233	**Iso-surface (cm^2)**	**0.238**	Iso-surface (cm^2)	0.180
	5.2-9.6		2.6-4.7			
	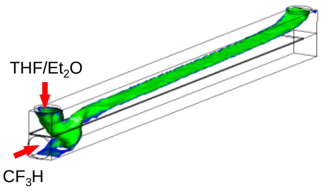		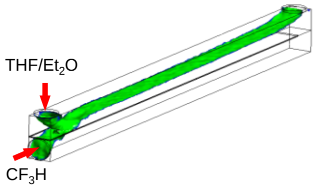			
	Iso-surface (cm^2)	0.227	Iso-surface (cm^2)	0.225		

Volume fraction of CF_3H

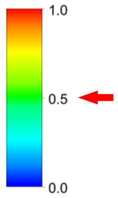

Supplementary Table 2. Dependence of iso-surface on the distance and height of baffle structures for GLD-1 (3.0 mL/min of THF; 14.6 mL/min of CF_3H, dark blue color: dead volume).

	BS-1	BS-2	BS-3	**BS-4**
	Baffle Structure	Baffle Structure	Baffle Structure	**Baffle Structure**
Distance (mm)	-	5.0	2.5	**2.0**
Height (μm)	1000 / 1000	1000 / 300	1000 / 300	**1000 / 300**
	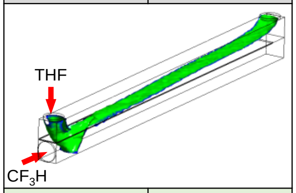	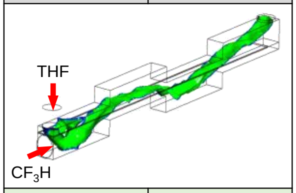	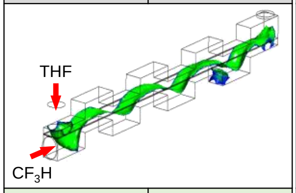	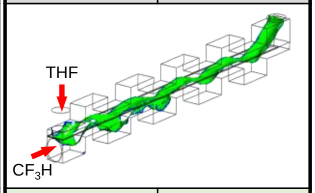
Iso-surface (cm^2)	0.222	0.233	0.245	**0.266**

	BS-5	BS-6	BS-7
	Baffle Structure	Baffle Structure	Baffle Structure
Distance (mm)	1.0	2.0	2.0
Height (μm)	1000 / 300	1000 / 100	1000 / 500
	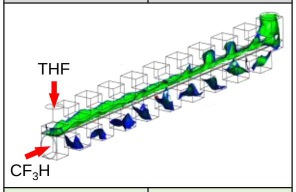	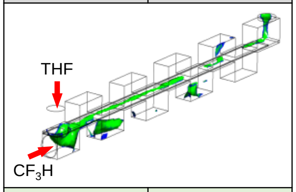	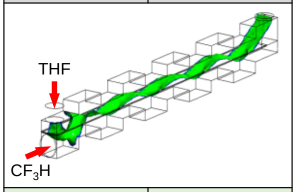
Iso-surface (cm^2)	0.254	0.134	0.206

Volume fraction of CF_3H

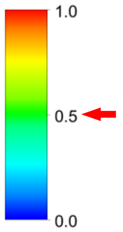

Supplementary Table 3. Dependence of iso-surface on the membrane porosity and thickness for GLD-1 (3.0 mL/min of THF; 14.6 mL/min of CF_3H, dark blue color: dead volume)

	**PM-1**	PM-2	PM-3	PM-4
Porosity (%)	**77**	70	64	60
Pore size (nm)	**820**	750	560	310
Thickness (μm)	**50**	50	50	50
	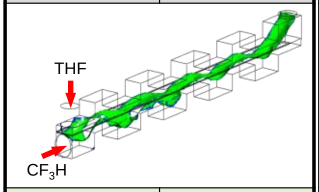	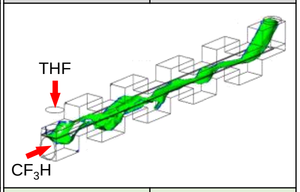	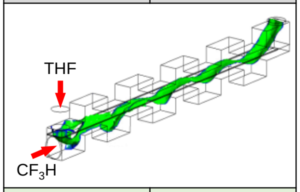	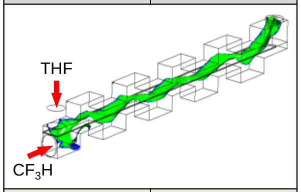
Iso-surface (cm^2)	**0.266**	0.263	0.233	0.219

	PM-5	PM-6	Without membrane
Porosity (%)	77	77	-
Pore size (nm)	820	820	-
Thickness (μm)	100	150	-
	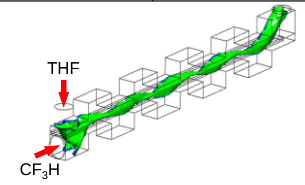	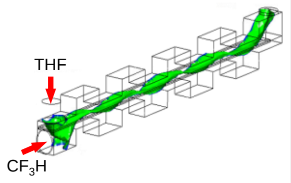	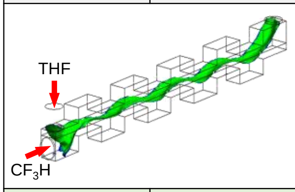
Iso-surface (cm^2)	0.222	0.218	0.209

Volume fraction of CF_3H

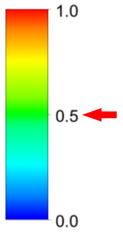

Supplementary Table 4. Dependence of iso-surface on the distance height of baffle structures for GLD-2 (7.8 mL/min of THF/Et_2O; 14.6 mL/min of CF_3H, dark blue color: dead volume)

	BS-1	BS-2	BS-3	**BS-4**	BS-5	BS-6	BS-7
	Baffle Structure	Baffle Structure	Baffle Structure	**Baffle Structure**	Baffle Structure	Baffle Structure	Baffle Structure
Distance (mm)	-	5.0	2.5	**2.0**	1.0	2.0	2.0
Height (µm)	1000 / 1000	1000 / 300	1000 / 300	**1000 / 300**	1000 / 300	1000 / 100	1000 / 500
	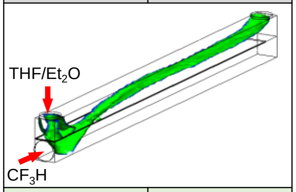	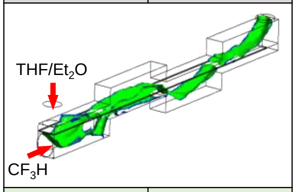	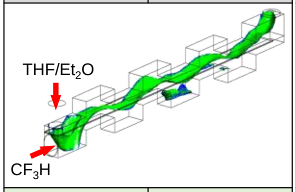	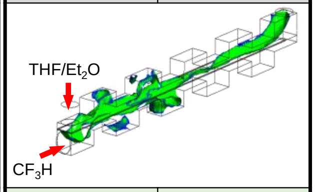	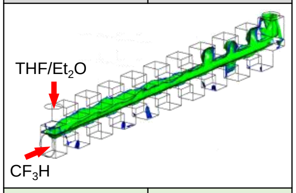	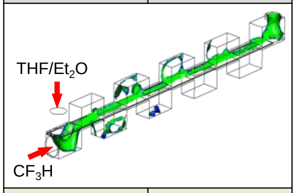	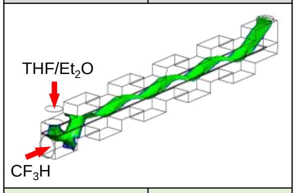
Iso-surface (cm^2)	0.238	0.242	0.245	**0.286**	0.283	0.244	0.203

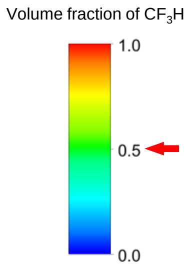

Supplementary Table 5. Dependence of iso-surface on the membrane porosity and thickness for GLD-2 (7.8 mL/min of THF/Et_2O; 14.6 mL/min of CF_3H, dark blue color: dead volume)

	**PM-1**	PM-2	PM-3	PM-4	PM-5	PM-6	Without membrane
Porosity (%)	**77**	70	64	60	77	77	-
Pore size (nm)	**820**	750	560	310	820	820	-
Thickness (µm)	**50**	50	50	50	100	150	-
	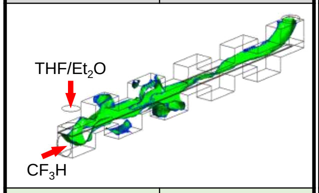	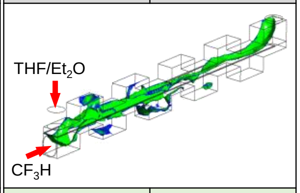	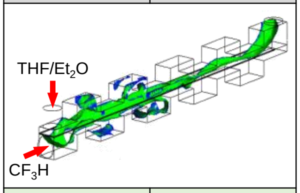	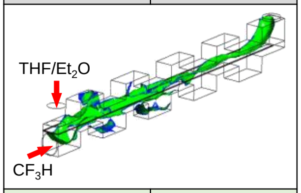	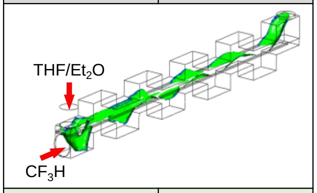	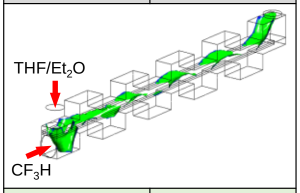	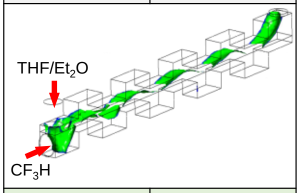
Iso-surface (cm^2)	**0.286**	0.270	0.267	0.265	0.177	0.161	0.168

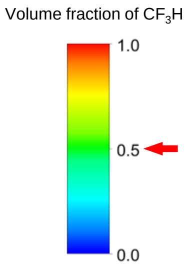

3. Fabrication of gas-liquid flow device (GLD)

Fabrication procedure. The gas-liquid flow device (GLD) with nano-porous membrane sandwiched between two staggered baffle structure was devised by adopting the BS-4 geometry as simulated. In order to fabricate the nano-porous membrane and baffle structure, perfluorinated polyether (PFPE) based nano-porous membrane and two baffle channels patterned stainless steel plates should be prepared and assembled by a modified method of the previous reports[4].

Fabrication of nano-porous membrane. Firstly, photocurable PFPE dimethacrylate was diluted with isopropanol (IPA). The weight ratio of PFPE dimethacrylate and IPA was 50:50 for 820 nm of pore size with 77% of overall porosity. The pore size and overall porosity were controlled by varying the weight ratio of PFPE dimethacrylate and IPA (Supplementary Figure 2)[5]. Then, photoinitiator Darocur 1173 was added (4% w/w of the PFPE mixture) to the mixture and stirred 5 min before casting. Next, the PFPE precursor-IPA mixture was dispensed into PDMS (polydimethylsiloxane; Sylgard 184 monomer from Dow corning with 9% w/w of curing agent) coated glass molds (height: 50 μm). The dispensed PFPE-IPA mixture was photo-cured by irradiation of UV light for 2 min with 20 mW/cm^2. Upon completion, the flat nano-porous PFPE thin membrane was demolded and placed in ethanol to sufficiently remove remained IPA in nano-pores. Next, the membrane was dried by supercritical CO_2. The nano-pore structure was analyzed by SEM, pore size and its distribution were measured based on SEM using ImageJ software, and the overall porosity was calculated by the equation as below:

$$Overall\ porosity, \varepsilon = 1 - \frac{Mp/\rho p}{w \times l \times t} \tag{8}$$

Where Mp is polymer mass, ρp is polymer density as 1.6 g/ml (at 25 °C), w is width, l is length, and t is thickness (Supplementary Figure 2a, 2b and 2c).

THF flux test of nano-porous membrane. The as-prepared nano-porous membrane was sandwiched between filter cups. 100 mL of THF was poured into the upper filter cup rapidly. Under the gravity force, THF flowed through the nano-porous membrane and collected by measuring cylinder. The time to collect 50 mL of THF was recorded and THF flux through the nano-porous membrane was calculated with following equation[6].

$$THF\ flux, J = \frac{\Delta V}{A \times \Delta t} \tag{9}$$

Where, ΔV refers the volume of THF collected in the measuring cylinder, A means the effective membrane area (12.57 cm^2), and Δt means the time to collect 50 mL of THF. (Supplementary Figure 1f)

CF_3H permeation test of nano-porous membrane.

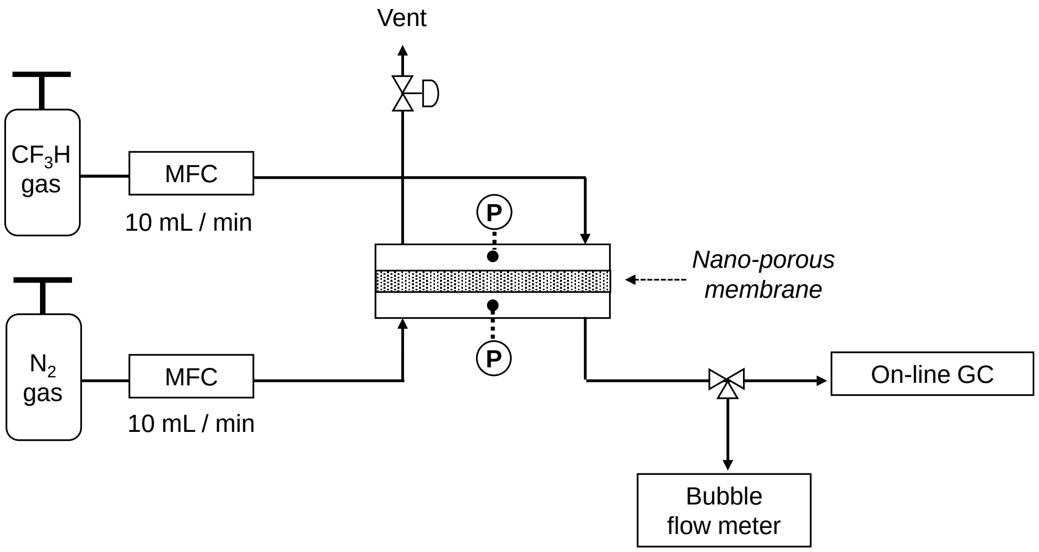

Supplementary Figure 1. The scheme of CF_3H permeation test of nano-porous membrane. The permeation test of CF_3H through nano-porous membrane was conducted with Wicke-Kallenbach apparatus[7].

CF_3H gas was introduced into the cell with fixed flow rate as 10 mL/min and nitrogen gas was introduced into the permeate side of the membrane at fixed flow rate as 10 mL/min. After equilibrium was reached, concentration of permeate CF_3H in gas mixture (CF_3H and nitrogen) was measured via on-line gas chromatography and flow rate of gas mixture was measured via bubble flow meter. Also, real-time pressure of each side of cell was measured by pressure sensor. The CF_3H gas permeance was calculated with following equation[8].

$$CF_3H\ permeance, P = \frac{N}{A \times \Delta p} \quad (10)$$

Where, N refers the molar flow rate of the gas mixture, A means the effective membrane area (3.14 cm^2), and Δp means the transmembrane pressure. (Supplementary Figure 1g)

Durability test in cryogenic temperature. The nano-porous membrane was immersed in the cooling bath of –95 °C for 2, 4, 6, and 8 hours. After that, the membrane was dried by supercritical CO_2. The changing rate of overall porosity was calculated and nano-porous structure was analyzed by SEM (Supplementary Figure 1d and 1e).

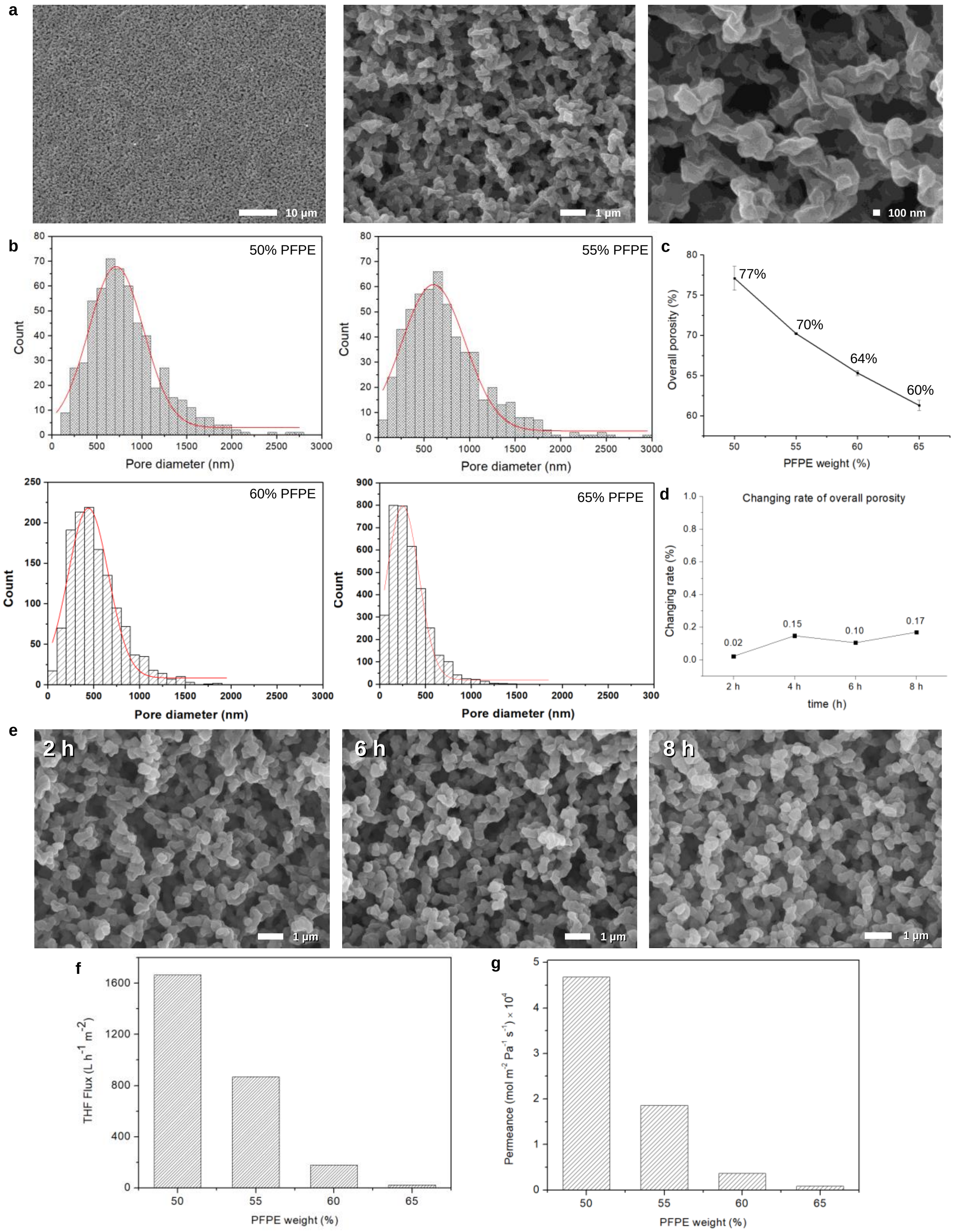

Supplementary Figure 2. Characterization data of PFPE nano-pore membrane. **a,** SEM images of 820 nm pore size of PFPE membrane (50 wt% of PFPE). **b,** A Plot of pore size distribution. **c,** A plot of overall porosity against wt% of PFPE. **d,** A plot of changing rate of overall porosity against time at –95 °C. **e,** SEM images of the membrane against time at –95 °C. **f**, A plot of THF flux against wt% of PFPE. **g**, A plot of CF_3H permeation against wt% of PFPE.

Fabrication of baffle channel structure. The stainless plates were patterned by computer numerical control (CNC) milling and the stage was controlled to partially ablate the 1 cm thick stainless steel plate by Vertical Machining Center (DNM 750, Doosan Machine Tools). The milling was preceded repeatedly with 300 μm and 1000 μm height to fabricate staggered baffle spaces with 2.0 mm of interval distance.

The prepared nano-porous thin PFPE membrane was sandwiched between baffle structure patterned stainless steel plates with precise alignment and clamped to connect inlet and outlet tubes.

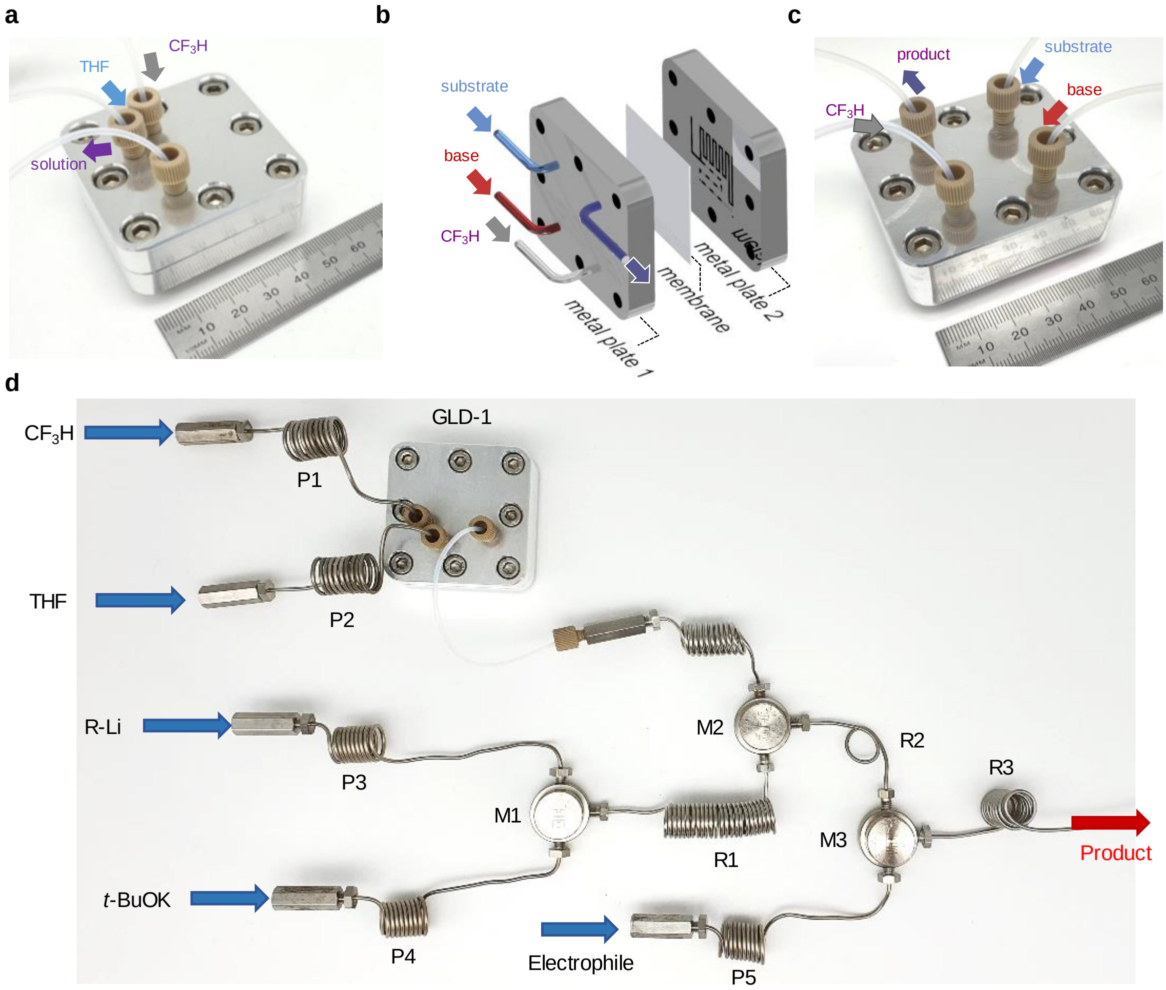

Supplementary Figure 3. Fabrication of GLD. **a,** An optical image of GLD-1 assembly with in- and outlet tubes. **b,** Illustration of scheme for GLD-2 via highly permeable membrane and staggered baffle channels. **c,** An optical image of GLD-2 assembly with in- and outlet tubes. **d,** A picture of the flow system combined with GLD-1.

4. Quantitative analysis of CF_3H solution in THF through GLD-1

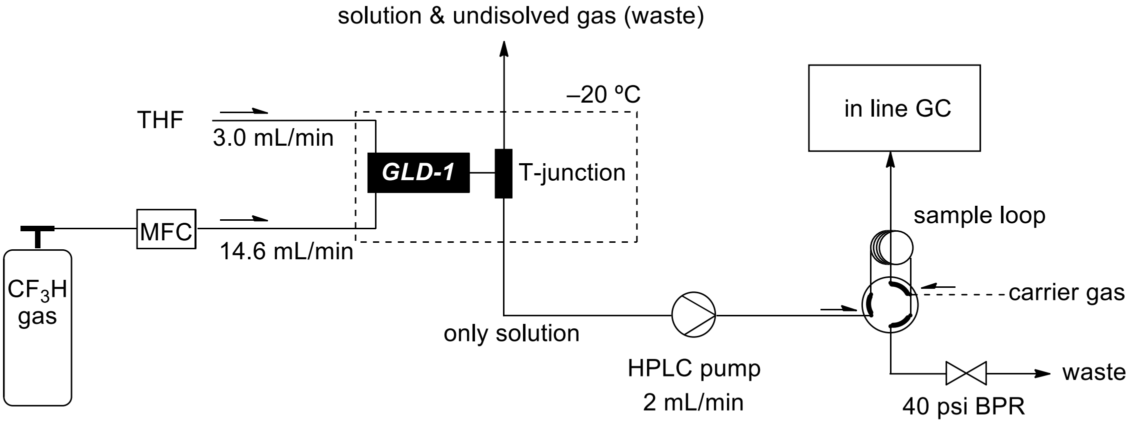

Supplementary Figure 4. The dissolution test of CF_3H in THF through GLD-1. The dissolution test was conducted varying inner volume by changing the channel length of GLD-1..

The dissolution test was conducted varying inner volume (650 μL to 65 μL) by changing the channel length (50 cm to 5 cm) of GLD-1 and general capillary tube. THF (flow rate: 3.0 mL/min) and CF_3H (flow rate: 14.6 mL/min) were introduced to two inlets of the GLD-1 device by the syringe pump and MFC, respectively. The resulting solution and the excess CF_3H gas was separated by a custom made gas-liquid separator (T-junction; diameter ϕ = 1.575 mm)[9]. The resulting separated solution supplied by HPLC pump (flow rate: 2 mL/min) and passed through sample loop via 6-port valve. Back pressure regulator (BPR; 40 psi) was installed to restrain the vaporizing of CF_3H gas from dissolved solution. A small amount of resulting solution (10 μL) was collected to sample loop and analyzed by GC.

Supplementary Table 6. Quantitative analysis of CF_3H in THF under –20 °C

Entry	Inner volume (μL)	Residence time (s)	Device	Baffle type[a]	Dissolution (%)
1	650	13.0	Capillary	-	95
2	390	7.8	Capillary	-	93
3	260	5.2	Capillary	-	91
4	130	2.6	Capillary	-	89
5	65	1.3	Capillary	-	87
6	650	13.0	GLD-1	BS-4	99
7	390	7.8	GLD-1	BS-4	99
8	260	5.2	GLD-1	BS-4	99
9	130	2.6	GLD-1	BS-4	98
**10**	**65**	1.3	**GLD-1**	**BS-4**	**98**
11	65	1.3	GLD-1	BS-5	93
12	65	1.3	GLD-1	BS-3	96
13	65	1.3	GLD-1	BS-2	95
14	65	1.3	GLD-1	BS-1	92
15[b]	65	1.3	GLD-1	BS-4	91

[a] No baffle structure for BS-1. 5.0 mm of baffle distance for BS-2. 2.5 mm of baffle distance for BS-3. 2.0 mm of baffle distance for BS-4. 1.0 mm of baffle distance of BS-5. [b] An absence of the membrane.

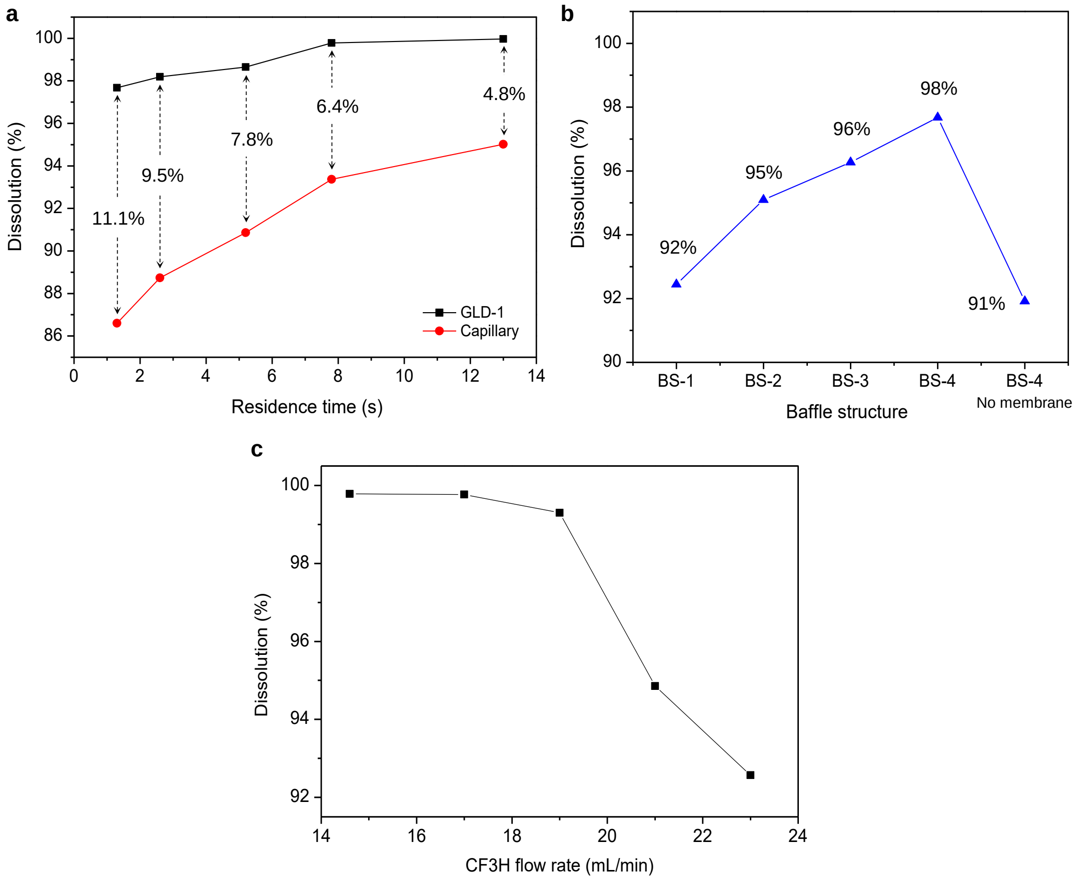

Supplementary Figure 5. Quantitative analysis of CF_3H in THF. **a,** A plot of dissolution of CF_3H against residence time in GLD-1 and capillary tube. **b,** A plot of dissolution of CF_3H against baffle structure in GLD-1. **c**, A plot of dissolution of CF_3H against CF_3H flow rate at fixed THF flow rate as 3 mL/min.

5. Generation of bare CF_3 anion from CF_3H

Generation of CF_3^- from CF_3H using bases and subsequent reaction with benzophenone in flow

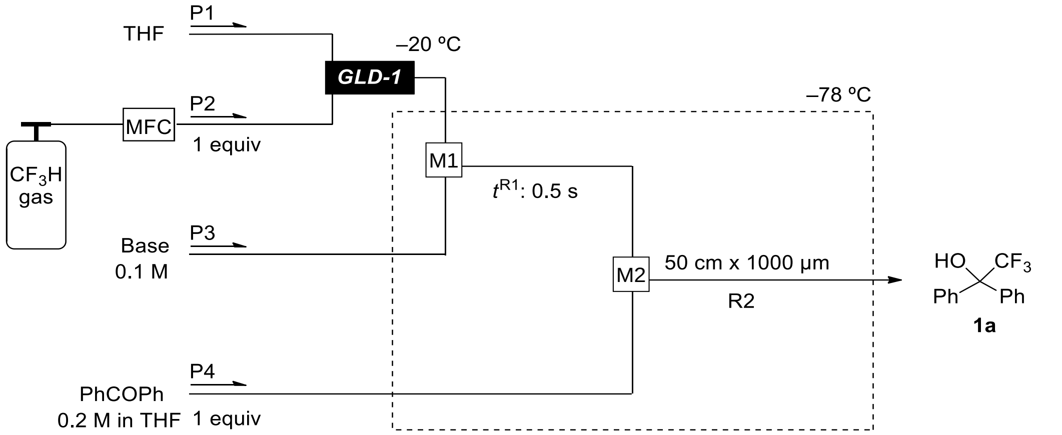

Supplementary Figure 5. Generation of CF_3^- from CF_3H using bases and subsequent reaction with benzophenone in flow. For the effective heat transfer to a cooling bath (–78 °C), four pre-cooling stainless steel tubing (P1, P2, P3, and P4: inner diameter ϕ: 1 mm, length L: 50 cm).

A microfluidic device consisting of three T-shaped micromixers (M1 and M2), two microreactors (R1 and R2), one GLD-1 was used. THF (flow rate: 3.0 mL/min) and CF_3H (flow rate: 14.6 mL/min) were introduced to two inlets of the GLD-1 device by the syringe pump and MFC, respectively. The resulting CF_3H solution in THF was mixed in M1 (inner diameter ϕ = 250 μm) with a solution of base (flow rate: 6.0 mL/min). The resulting solution was passed through R1 (ϕ = 1000 μm, L = 10 cm) and reacted with a solution of benzophenone (0.2 M in THF, flow rate: 3.0 mL/min) in M2 (ϕ = 500 μm) and R2 (ϕ = 1000 μm, L = 50 cm). After a steady-state was reached (90 s), the product solution was collected for 30 s while being quenched with saturated NH_4Cl aqueous solution (2 mL).

Supplementary Table 7. Direct deprotonation of CF_3H using the base in flow

Entry	Base	Yield of **1a** (%)[a]	Yield of **2** (%)[a]
1	*t*-BuOK	*n.d.*	*n.d.*
2	KHMDS	*n.d.*	*n.d.*
3	LDA	*n.d.*	*n.d.*
4	LiTMP	*n.d*	*n.d*
5	MeLi	*n.d.*	95
6	PhLi	*n.d.*	98
7	*n*-BuLi	*n.d.*	77
8	*s*-BuLi	*n.d.*	71
9	*t*-BuLi	*n.d.*	71

[a] GC yield.

Generation of CF_3^- from CF_3H using superbase and subsequent reaction with benzophenone in flow

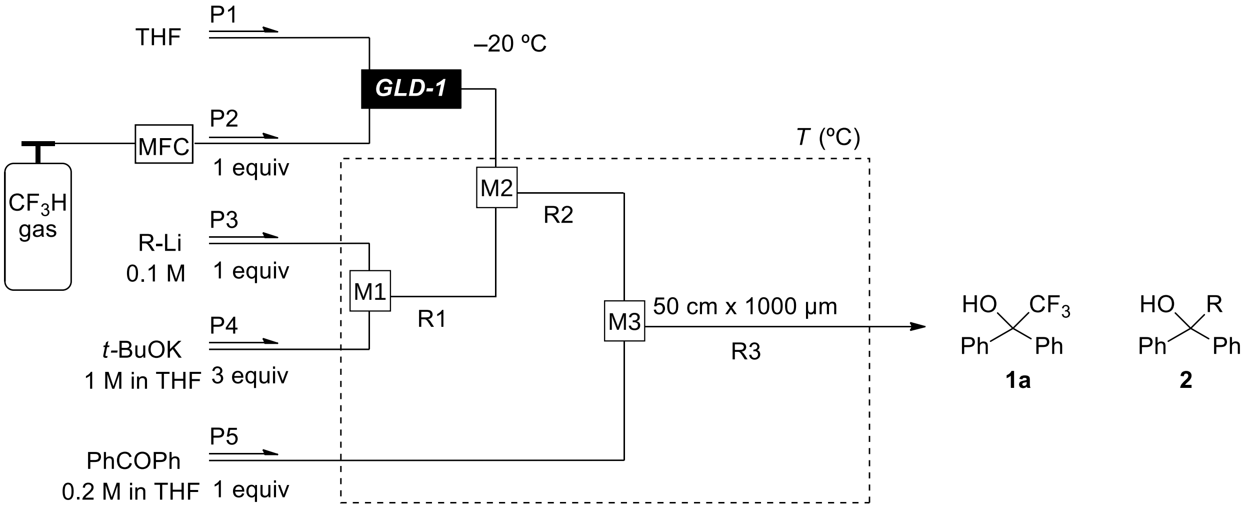

Supplementary Figure 6. Generation of CF_3^- from CF_3H using superbase and subsequent reaction with benzophenone in flow. For the effective heat transfer to a cooling bath (–95, –78, –60, –40 or –20 °C), five pre-cooling stainless steel tubing (P1, P2, P3, P4, and P5: inner diameter ϕ: 1 mm, length L: 50 cm)

A microfluidic device consisting of three T-shaped micromixers (M1, M2, and M3), three microreactors (R1, R2, and R3), one GLD-1 was used. THF (flow rate: 3.0 mL/min) and CF_3H (flow rate: 14.6 mL/min) were introduced to two inlets of GLD-1 device by the syringe pump and MFC respectively. The resulting CF_3H solution in THF was mixed in M2 (inner diameter ϕ = 250 μm) with a mixing solution of organolithium (flow rate: 6.0 mL/min) and *t*-BuOK in THF (flow rate: 1.8 mL/min) which was prepared in M1 (ϕ = 250 μm) and R1 (ϕ = 500 μm or 1000 μm, L = 3.5, 10, 50, 100 or 200 cm). The resulting solution was passed through R2 (ϕ = 500 μm or 1000 μm, L = 3.5, 10, 50 or 150 cm) and reacted with a solution of benzophenone (0.2 M in THF, 3.0 mL/min) in M3 (ϕ = 500 μm) and R3 (ϕ = 1000 μm, L = 50 cm). After a steady-state was reached (90 s), the product solution was collected for 30 s while being quenched with saturated NH_4Cl aqueous solution (2 mL).

For GC yield, 1,3,5-trimethoxybenzene (50 mg) was added to a product solution as an internal standard. Et_2O (6 mL) and brine (2 mL) were added and the organic phase was separated. An aliquot (2 mL) of the organic phase was analyzed by GC spectroscopy (Table S5).
For isolation of the desired product(s), the organic layer of the product solution was separated and the aqueous layer was extracted with Et_2O (20 mL×3). The combined organic layers were dried over Na_2SO_4 and concentrated. The crude product was purified by column chromatography.

Supplementary Table 8. Deprotonation of CF_3H with various bases and the subsequent reaction with benzophenone in the flow reactor under various conditions.

entry	R-Li	T(°C)	t^{R1} (s)	t^{R2} (s)	Yield of **1a** (%)[a]	Yield of **2** (%)[a]	remain E^+ (%)[a]
1	BuLi	–20	6.0	0.04	*n.d.*	*n.d.*	100
2	BuLi	–20	6.0	0.15	*n.d.*	*n.d.*	100
3	BuLi	–20	6.0	0.44	*n.d.*	*n.d.*	100
4	BuLi	–20	6.0	2.2	*n.d.*	*n.d.*	100
5	BuLi	–20	6.0	4.4	*n.d.*	*n.d.*	100
6	BuLi	–20	6.0	6.5	*n.d.*	*n.d.*	100
7	BuLi	–40	6.0	0.04	4	*n.d.*	96
8	BuLi	–40	6.0	0.15	2	*n.d.*	97
9	BuLi	–40	6.0	0.44	1	*n.d.*	98
10	BuLi	–40	6.0	2.2	*n.d.*	*n.d.*	100
11	BuLi	–40	6.0	4.4	*n.d.*	*n.d.*	100
12	BuLi	–40	6.0	6.5	*n.d.*	*n.d.*	100
13	BuLi	–60	6.0	0.04	59	*n.d.*	41
14	BuLi	–60	6.0	0.15	46	*n.d.*	54
15	BuLi	–60	6.0	0.44	33	*n.d.*	66
16	BuLi	–60	6.0	2.2	19	*n.d.*	81
17	BuLi	–60	6.0	4.4	11	*n.d.*	89
18	BuLi	–60	6.0	6.5	1	*n.d.*	98
19	BuLi	–78	6.0	0.04	51	*n.d.*	46
20	BuLi	–78	6.0	0.15	57	*n.d.*	41
21	BuLi	–78	6.0	0.44	52	*n.d.*	48
22	BuLi	–78	6.0	2.2	40	*n.d.*	59
23	BuLi	–78	6.0	4.4	32	*n.d.*	68
24	BuLi	–78	6.0	6.5	26	*n.d.*	73
25	BuLi	–95	6.0	0.04	57	*n.d.*	42
26	BuLi	–95	6.0	0.15	62	*n.d.*	35
27	BuLi	–95	6.0	0.44	67	*n.d.*	32
28	BuLi	–95	6.0	2.2	60	*n.d.*	37
29	BuLi	–95	6.0	4.4	52	*n.d.*	48
30	BuLi	–95	6.0	6.5	32	*n.d.*	68
31	BuLi	–95	0.05	0.44	25	8	66
32	BuLi	–95	0.2	0.44	24	8	67
33	BuLi	–95	0.6	0.44	27	5	68
34	BuLi	–95	3.0	0.44	49	3	45

35	BuLi	–95	6.0	0.44	67	4	25
36	BuLi	–95	12.1	0.44	54	3	42
37	*s*-BuLi	–78	6.0	0.04	48	*n.d.*	52
38	*s*-BuLi	–78	6.0	0.15	42	*n.d.*	54
39	*s*-BuLi	–78	6.0	0.44	34	*n.d.*	64
40	*s*-BuLi	–78	6.0	2.2	22	*n.d.*	72
41	*s*-BuLi	–78	6.0	4.4	13	*n.d.*	85
42	*s*-BuLi	–78	6.0	6.5	10	*n.d.*	89
43	PhLi	–20	6.0	0.04	11	*n.d.*	88
44	PhLi	–20	6.0	0.15	3	*n.d.*	97
45	PhLi	–20	6.0	0.44	1	*n.d.*	99
46	PhLi	–20	6.0	2.2	*n.d.*	*n.d.*	100
47	PhLi	–20	6.0	4.4	*n.d.*	*n.d.*	100
48	PhLi	–20	6.0	6.5	*n.d.*	*n.d.*	100
49	PhLi	–40	6.0	0.04	32	12	55
50	PhLi	–40	6.0	0.15	19	8	73
51	PhLi	–40	6.0	0.44	15	1	76
52	PhLi	–40	6.0	2.2	3	1	94
53	PhLi	–40	6.0	4.4	*n.d.*	*n.d.*	100
54	PhLi	–40	6.0	6.5	*n.d.*	*n.d.*	100
55	PhLi	–60	6.0	0.04	61	6	30
56	PhLi	–60	6.0	0.15	47	4	43
57	PhLi	–60	6.0	0.44	39	*n.d.*	60
58	PhLi	–60	6.0	2.2	28	*n.d.*	72
59	PhLi	–60	6.0	4.4	11	*n.d.*	88
60	PhLi	–60	6.0	6.5	8	*n.d.*	91
61	PhLi	–78	6.0	0.04	51	16	32
62	PhLi	–78	6.0	0.15	81	4	17
63	PhLi	–78	6.0	0.44	67	*n.d.*	32
64	PhLi	–78	6.0	2.2	53	*n.d.*	42
65	PhLi	–78	6.0	4.4	35	*n.d.*	52
66	PhLi	–78	6.0	6.5	32	*n.d.*	54
67	PhLi	–95	6.0	0.04	58	21	12
68	PhLi	–95	6.0	0.15	89	7	1
**69**	**PhLi**	**–95**	**6.0**	**0.44**	**93**	***n.d.***	**2**
70	PhLi	–95	6.0	2.2	80	*n.d.*	15
71	PhLi	–95	6.0	4.4	78	*n.d.*	20

72	PhLi	–95	6.0	6.5	75	*n.d.*	25
73	PhLi	–95	0.05	0.44	30	2	67
74	PhLi	–95	0.2	0.44	31	2	68
75	PhLi	–95	0.6	0.44	38	3	58
76	PhLi	–95	3.0	0.44	70	2	27
77	PhLi	–95	6.0	0.44	93	*n.d.*	2
78	PhLi	–95	12.1	0.44	85	*n.d.*	10
79[b]	PhLi	–95	6.0	0.44	34	24	38
80[c]	PhLi	–95	6.0	0.44	74	16	16

The conversion was calculated based on benzophenone. [a] GC yield. [b] 1 equiv. of *t*-BuOK. [c] 2 equiv. of *t*-BuOK.

Generation of CF_3^- by direct use of CF_3H and subsequent reaction with benzophenone in GLD-2

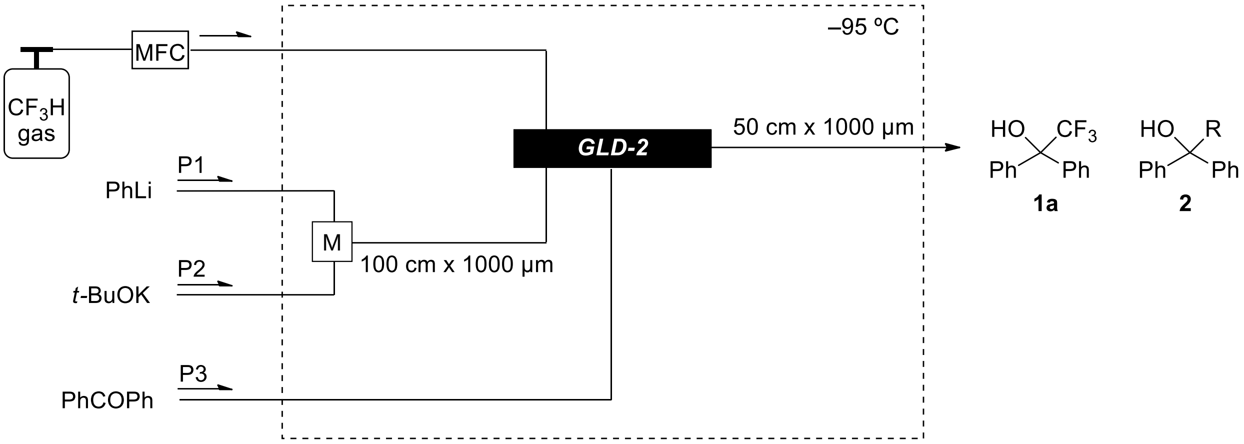

Supplementary Figure 7. Generation of CF_3^- by direct use of CF_3H and subsequent reaction with benzophenone in GLD-2. For the effective heat transfer to a cooling bath (–95 °C), four pre-cooling stainless steel tubings (P1, P2, and P3: inner diameter ϕ: 1 mm, length L: 50 cm) were used.

A solution of PhLi (0.13 M in Et_2O, 6.0 mL/min) and a solution of *t*-BuOK (1.3 M in THF, 1.8 mL/min) were introduced to M (inner tube ϕ = 250 µm) by syringe pumps and passed through tube reactor (ϕ = 1000 µm, L = 100 cm). The resulting solution and CF_3H (flow rate: 14.6 mL/min) were introduced to two inlets of the GLD-2 device and CF_3H was introduced by MFC. The resulting solution was passed through a nano-porous membrane sandwiched by a staggered baffle structure and was mixed with a solution of benzophenone (0.3 M in THF, 3.0 mL/min). The resulting solution was passed through tube reactor (ϕ = 1000 µm, L = 50 cm). After a steady-state was reached (90 s), the product solution was collected for 30 s while being quenched with saturated NH_4Cl aqueous solution (2 mL).

6. Reaction with various electrophilic substrates

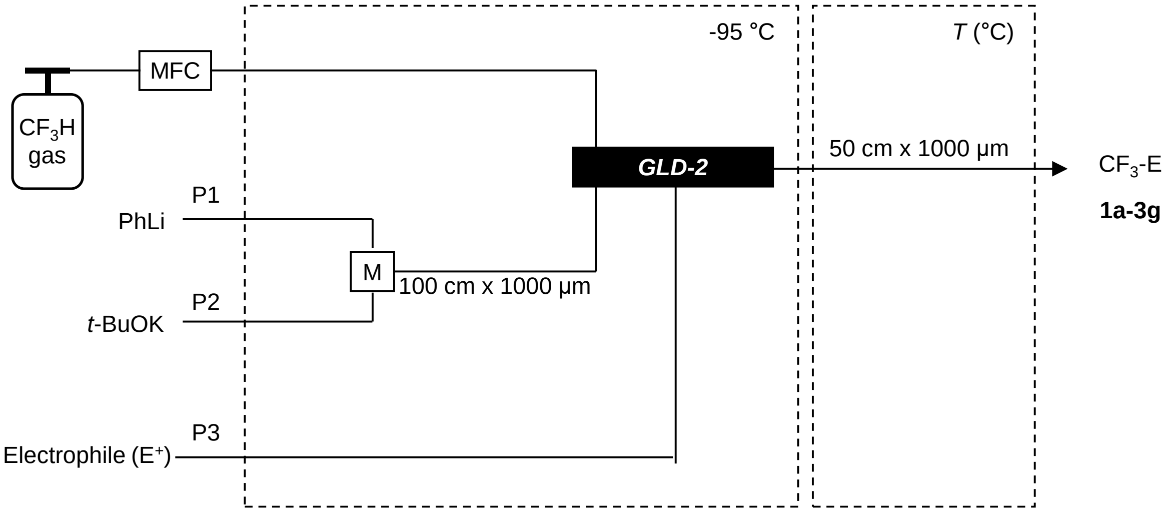

Supplementary Figure 8. Reaction with various electrophilic substrate using GLD-2. For the effective heat transfer to a cooling bath (–95 °C), four pre-cooling stainless steel tubings (P1, P2, and P3: inner diameter ϕ: 1 mm, length L: 50 cm) were used.

The reaction occurred with various electrophiles in GLD-2. The temperature T (°C) was –95 °C unless other mentioned. For isolation of the desired products, the organic layer of the product solution was separated and the aqueous layer was extracted with Et_2O (15 mL×3). The combined organic layer was dried over Na_2SO_4 and concentrated. The crude product was purified by column chromatography or distillation.

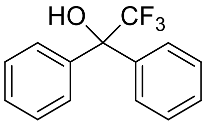

2,2,2-Trifluoro-1,1-diphenylethanol (1a)

The product **1a** was obtained in 83% isolated yield (62.8 mg) as a colorless oil.

^{1}H NMR (500 MHz, $CDCl_3$) δ 7.50–7.49 (m, 4H), 7.37–7.35 (m, 6H), 2.68 (s, 1H) ppm; ^{13}C NMR (125 MHz, $CDCl_3$) δ 139.5, 128.7, 128.4, 125.5 (q, $J_{C\text{-}F}$ = 287 Hz), 79.6 (q, $J_{C\text{-}CF3}$ = 29 Hz) ppm; ^{19}F NMR (470 MHz, $CDCl_3$) δ –74.3 (s, CF_3) ppm; HRMS (EI) (*m/z*) cald. for $C_{14}H_{11}F_3O$, 252.0762; found, 252.0759.

The spectral data were identical to those reported in the literature[10].

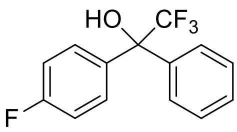

2,2,2-Trifluoro-1-(4-fluorophenyl)-1-phenylethanol (1b)

The product **1b** was obtained in 82% isolated yield (66.5 mg) as a colorless oil.

^{1}H NMR (500 MHz, $CDCl_3$) δ 7.49–7.46 (m, 4H), 7.39–7.36 (m, 3H), 7.06–7.02 (m, 2H), 2.87 (s, 1H) ppm; ^{13}C NMR (125 MHz, $CDCl_3$) δ 163.7, 161.8, 139.3, 135.1, 129.6, 129.5, 128.9, 128.5, 127.3, 125.2 (q, $J_{C\text{-}F}$ = 284 Hz), 115.3, 115.1, 79.1 (q, $J_{C\text{-}CF3}$ = 29 Hz) ppm; ^{19}F NMR (470 MHz, $CDCl_3$) δ –75.0 (s, CF_3), –113.7 (m, Ar-F) ppm; HRMS (EI) (*m/z*) cald. for $C_{14}H_{10}F_4O$, 270.0668; found, 270.0670.

The spectral data were identical to those reported in the literature[10].

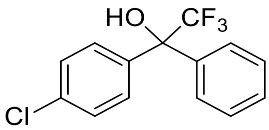

1-(4-Chlorophenyl)-2,2,2-trifluoro-1-phenylethanol (1c)

The product **1c** was obtained in 82% isolated yield (70.5 mg) as a colorless oil.

^{1}H NMR (500 MHz, $CDCl_3$) δ 7.48–7.46 (m, 2H), 7.43–7.42 (m, 2H), 7.39–7.37 (m, 3H), 7.35–7.32 (m, 2H), 2.86 (s, 1H) ppm; ^{13}C NMR (125 MHz, $CDCl_3$) δ 139.0, 137.7, 134.8, 128.99, 128.95, 128.5, 128.4, 127.3, 125.1 (q, $J_{C\text{-}F}$ = 285 Hz), 79.2 (q, $J_{C\text{-}CF3}$ = 29 Hz) ppm; ^{19}F NMR (470 MHz, $CDCl_3$) δ –74.4 (s, CF_3) ppm; HRMS (EI) (*m/z*) cald. for $C_{14}H_{10}ClF_3O$, 286.0372; found, 286.0370.

The spectral data were identical to those reported in the literature[11].

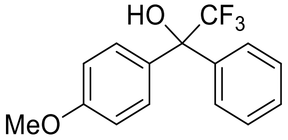

2,2,2-Trifluoro-1-(4-methoxyphenyl)-1-phenylethanol (1d)

The product **1d** was obtained in 80% isolated yield (67.7 mg) as a colorless oil.

^{1}H NMR (500 MHz, $CDCl_3$) δ 7.50–7.48 (m, 2H), 7.40–7.35 (m, 5H), 6.88–6.87 (m, 2H), 3.81 (s, 3H), 2.79 (s, 1H) ppm; ^{13}C NMR (125 MHz, $CDCl_3$) δ 159.6, 139.6, 131.6, 128.9, 128.6, 128.2, 127.5, 125.4 (q, $J_{C\text{-}F}$ = 285 Hz), 113.6, 79.2 (q, $J_{C\text{-}CF3}$ = 29 Hz), 55.3 ppm; ^{19}F NMR (470 MHz, $CDCl_3$) δ –74.4 (s, CF_3) ppm; HRMS (EI) (*m/z*) cald. for $C_{15}H_{13}F_3O_2$, 283.0868; found, 282.0868.

The spectral data were identical to those reported in the literature[10].

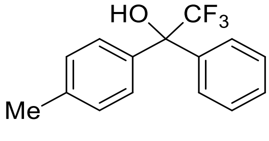

2,2,2-Trifluoro-1-phenyl-1-(*p*-tolyl)ethanol (1e)

The product **1e** was obtained in 81% isolated yield (64.7 mg) as a colorless oil.

^{1}H NMR (500 MHz, $CDCl_3$) δ 7.50–7.48 (m, 2H), 7.40–7.35 (m, 5H), 7.18–7.16 (m, 2H), 2.81 (s, 1H), 2.35 (s, 3H) ppm; ^{13}C NMR (125 MHz, $CDCl_3$) δ 139.5, 138.6, 136.5, 129.0, 128.6, 128.2, 127.4, 127.3,

125.4 (q, $J_{C\text{-}F}$ = 285 Hz), 79.4 (q, $J_{C\text{-}CF3}$ = 29 Hz), 21.1 ppm; ^{19}F NMR (470 MHz, $CDCl_3$) δ –74.4 (s, CF_3) ppm; HRMS (EI) (*m/z*) cald. for $C_{15}H_{13}F_3O$, 266.0918; found, 266.0916.

The spectral data were identical to those reported in the literature[10].

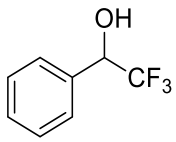

2,2,2-Trifluoro-1-phenylethanol (1f)

The product **1f** was obtained in 71% isolated yield (37.5 mg) as a bright yellow oil.

^{1}H NMR (500 MHz, $CDCl_3$) δ 7.49–7.47 (m, 2H), 7.43–7.41 (m, 3H), 5.04–4.99 (m, 1H), 2.58 (d, *J* = 4.4 Hz, 1H) ppm; ^{13}C NMR (125 MHz, $CDCl_3$) δ 134.1, 129.6, 128.6, 127.5, 124.3 (q, $J_{C\text{-}F}$ = 280 Hz), 72.7 (q, $J_{C\text{-}CF3}$ = 31 Hz) ppm; ^{19}F NMR (470 MHz, $CDCl_3$) δ –78.4 (d, $J_{F\text{-}H}$ = 6.2 Hz) ppm; HRMS (EI) (*m/z*) cald. for $C_8H_7F_3O$, 176.0449; found, 176.0452.

The spectral data were identical to those reported in the literature[12].

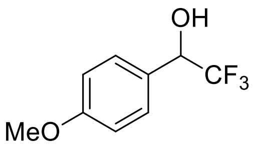

2,2,2-Trifluoro-1-(4-methoxyphenyl)ethanol (1g)

The product **1g** was obtained in 69% isolated yield (42.7 mg) as a colorless oil.

^{1}H NMR (500 MHz, $CDCl_3$) δ 7.40 (d, *J* = 8.5 Hz, 2H), 6.93 (d, *J* = 9.0 Hz, 2H), 4.98–4.96 (m, 1H), 3.82 (s, 3H), 2.48 (br s, 1H) ppm; ^{13}C NMR (125 MHz, $CDCl_3$) δ 160.5, 128.8, 126.13, 124.4 (q, $J_{C\text{-}F}$ = 280 Hz), 114.1, 72.5 (q, $J_{C\text{-}CF3}$ = 33 Hz), 55.3 ppm; ^{19}F NMR (470 MHz, $CDCl_3$) δ –78.6 (d, $J_{F\text{-}H}$ = 6.9 Hz) ppm; HRMS (EI) (*m/z*) cald. for $C_9H_9F_3O_2$, 206.0555; found, 206.0557.

The spectral data were identical to those reported in the literature[10].

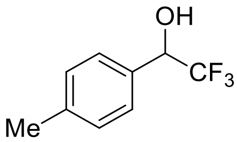

2,2,2-Trifluoro-1-(*p*-tolyl)ethanol (1h)

The product **1h** was obtained in 70% isolated yield (39.9 mg) as a colorless oil.

^{1}H NMR (500 MHz, $CDCl_3$) δ 7.36 (d, *J* = 7.5 Hz, 2H), 7.22 (d, *J* = 7.9 Hz, 2H), 4.99–4.98 (m, 1H), 2.45 (br s, 1H), 2.38 (s, 1H) ppm; ^{13}C NMR (125 MHz, $CDCl_3$) δ 139.6, 131.0, 129.4, 127.3, 124.3 (q, $J_{C\text{-}F}$ = 280 Hz), 72.7 (q, $J_{C\text{-}CF3}$ = 32 Hz), 21.2 ppm; ^{19}F NMR (470 MHz, $CDCl_3$) δ –78.4 (d, $J_{F\text{-}H}$ = 6.8 Hz) ppm; HRMS (EI) (*m/z*) cald. for $C_9H_9F_3O$, 190.0605; found, 190.0603.

The spectral data were identical to those reported in the literature[13].

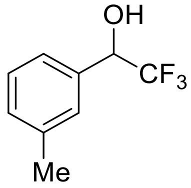

2,2,2-Trifluoro-1-(*m*-tolyl)ethanol (1i)

The product **1i** was obtained in 62% isolated yield (35.4 mg) as a colorless oil.

^{1}H NMR (500 MHz, $CDCl_3$) δ 7.31–7.21 (m, 4H), 4.96 (q, J = 6.8 Hz, 1H), 2.57 (br s, 1H), 2.38 (s, 1H) ppm; ^{13}C NMR (125 MHz, $CDCl_3$) δ 138.5, 133.9, 130.3, 128.5, 128.0, 124.5, 124.3 (q, $J_{C\text{-}F}$ = 281 Hz), 72.8 (q, $J_{C\text{-}CF3}$ = 32 Hz), 21.4 ppm; ^{19}F NMR (470 MHz, $CDCl_3$) δ –78.3 (d, $J_{F\text{-}H}$ = 6.5 Hz) ppm; HRMS (EI) (*m/z*) cald. for $C_9H_9F_3O$, 190.0605; found, 190.0608.

The spectral data were identical to those reported in the literature[10].

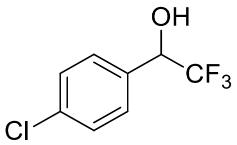

1-(4-Chlorophenyl)-2,2,2-trifluoroethanol (1j)

The product **1j** was obtained in 64% isolated yield (40.4 mg) as a yellowish oil.

^{1}H NMR (500 MHz, $CDCl_3$) δ 7.44–7.36 (m, 4H), 4.96 (q, J = 6.6 Hz, 1H), 2.95 (br s, 1H) ppm; ^{13}C NMR (125 MHz, $CDCl_3$) δ 135.5, 132.5, 128.9, 128.8, 124.1 (q, $J_{C\text{-}F}$ = 280 Hz), 72.0 (q, $J_{C\text{-}CF3}$ = 32 Hz) ppm; ^{19}F NMR (470 MHz, $CDCl_3$) δ –78.5 (d, $J_{F\text{-}H}$ = 6.8 Hz) ppm; HRMS (EI) (*m/z*) cald. for $C_8H_6ClF_3O$, 210.0059; found, 210.0061.

The spectral data were identical to those reported in the literature[14].

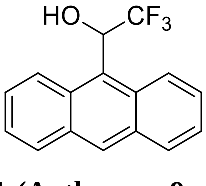

1-(Anthracen-9-yl)-2,2,2-trifluoroethanol (1k)

The product **1k** was obtained in 72% isolated yield (59.7 mg) as an off-white solid.

^{1}H NMR (500 MHz, $CDCl_3$) δ 8.96 (br s, 1H), 8.53 (s. 1H), 8.12 (br s, 1H), 8.02 (d, J = 8.3 Hz, 2H), 7.55–7.47 (m, 4H), 6.63 (q, J = 7.5 Hz, 1H), 3.0 (br s, 1H) ppm; ^{13}C NMR (125 MHz, $CDCl_3$) δ 131.9, 130.8, 129.5, 129.3, 127.2, 126.3, 125.5 (q, $J_{C\text{-}F}$ = 282 Hz), 125.0, 123.9, 122.5, 70.2 (q, $J_{C\text{-}CF3}$ = 33 Hz) ppm; ^{19}F NMR (470 MHz, $CDCl_3$) δ –74.0 (d, $J_{F\text{-}H}$ = 8.1 Hz) ppm; HRMS (EI) (*m/z*) cald. for $C_{16}H_{11}F_3O$, 276.0762; found, 276.0758.

The spectral data were identical to those reported in the literature[15].

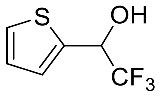

2,2,2-Trifluoro-1-(thiophen-2-yl)ethanol (1l)

The product **1l** was obtained in 51% isolated yield (27.9 mg) as a yellowish oil.

^{1}H NMR (500 MHz, $CDCl_3$) δ 7.39 (dd, J = 5.1, 1.2 Hz, 1H), 7.20 (m, 1H), 7.05 (dd, J = 5.1, 3.6 Hz, 1H), 5.27 (q, J = 6.3 Hz, 1H), 2.80 (br s, 1H) ppm; ^{13}C NMR (125 MHz, $CDCl_3$) δ 136.1, 128.6, 127.5, 127.1, 127.0, 123.7 (q, J_{C-F} = 281 Hz), 69.3 (q, J_{C-CF3} = 34 Hz) ppm; ^{19}F NMR (470 MHz, $CDCl_3$) δ –78.7 (d, J_{F-H} = 5.5 Hz) ppm; HRMS (EI) (*m/z*) cald. for $C_6H_5F_3OS$, 182.0013; found, 182.0013.

The spectral data were identical to those reported in the literature[16].

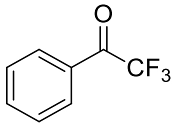

2,2,2-Trifluoro-1-phenylethanone (1m)

The product **1m** was obtained in 68% isolated yield (35.5 mg) as a colorless oil.

^{1}H NMR (500 MHz, $CDCl_3$) δ 8.07 (d, J = 7.9 Hz, 2H), 7.70 (td, J = 7.5, 1.3 Hz, 1H), 7.54 (m, 2H) ppm; ^{13}C NMR (125 MHz, $CDCl_3$) δ 180.5 (q, J_{C-F} = 35 Hz), 135.5, 130.1, 129.1, 116.7 (q, J_{C-CF3} = 290 Hz) ppm; ^{19}F NMR (470 MHz, $CDCl_3$) δ –71.5 (s, CF_3) ppm; HRMS (EI) (*m/z*) cald. for $C_8H_5F_3O$, 174.0292; found, 174.0293.

Spectrum were matched with commercial available 2,2,2-trifuloroacetophenone.

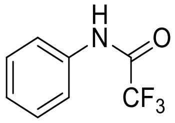

2,2,2-Trifluoro-*N*-phenylacetamide (1n)

The product **1n** was obtained in 73% isolated yield (41.4 mg) as a white solid.

^{1}H NMR (500 MHz, $CDCl_3$) δ 7.83 (br s, 1H), 7.58-7.56 (m, 2H), 7.41 (t, J = 7.6 Hz, 2H), 7.27–7.24 (m, 2H) ppm; ^{13}C NMR (125 MHz, $CDCl_3$) δ 154.8 (q, J_{C-F} = 38 Hz), 135.1, 129.4, 126.4, 120.5, 115.7 (q, J_{C-CF3} = 288 Hz) ppm; ^{19}F NMR (470 MHz, $CDCl_3$) δ –75.7 (s, CF_3) ppm; HRMS (EI) (*m/z*) cald. for $C_8H_6F_3NO$, 189.0401; found, 189.0403.

The spectral data were identical to those reported in the literature[17].

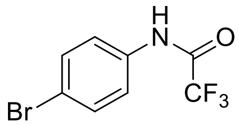

***N*-(4-Bromophenyl)-2,2,2-trifluoroacetamide (1o)**

The product **1o** was obtained in 60% isolated yield (48.2 mg) as a white solid.

^{1}H NMR (500 MHz, $CDCl_3$) δ 7.83 (br s, 1H), 7.53 (d, *J* = 9 Hz, 2H), 7.47 (d, *J* = 9 Hz, 2H) ppm; ^{13}C NMR (125 MHz, $CDCl_3$) δ 154.8 (q, $J_{C\text{-}F}$ = 38 Hz), 134.1, 132.5, 122.1, 119.5, 115.6 (q, $J_{C\text{-}CF3}$ = 288 Hz) ppm; ^{19}F NMR (470 MHz, $CDCl_3$) δ –75.7 (s, CF_3) ppm; HRMS (EI) (*m/z*) cald. for $C_8H_5BrF_3NO$, 266.9507; found, 266.9508.

The spectral data were identical to those reported in the literature[17].

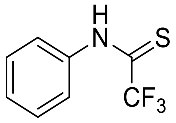

2,2,2-Trifluoro-*N*-phenylethanethioamide (1p)

The product **1p** was obtained in 92% isolated yield (56.6 mg) as a yellow oil.

^{1}H NMR (500 MHz, $CDCl_3$) δ 9.24 (br s, 1H), 7.77 (d, *J* = 7.5 Hz, 2H), 7.47 (t, *J* = 8.2 Hz, 2H), 7.36 (t, *J* = 7.4 Hz, 1H) ppm; ^{13}C NMR (125 MHz, $CDCl_3$) δ 180.4 (q, $J_{C\text{-}F}$ = 35 Hz), 136.5, 129.4, 128.1, 123.0, 117.4 (q, $J_{C\text{-}CF3}$ = 279 Hz) ppm; ^{19}F NMR (470 MHz, $CDCl_3$) δ –69.8 (s, CF_3) ppm; HRMS (EI) (*m/z*) cald. for $C_8H_6F_3NS$, 205.0173; found, 205.0175.

The spectral data were identical to those reported in the literature[18].

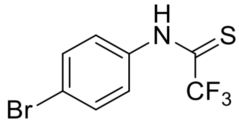

***N*-(4-Bromophenyl)-2,2,2-trifluoroethanethioamide (1q)**

The product **1q** was obtained in 90% isolated yield (76.7 mg) as a yellow solid.

^{1}H NMR (500 MHz, $CDCl_3$) δ 9.17 (br s, 1H), 7.69 (d, *J* = 9.0 Hz, 2H), 7.58 (d, *J* = 9.0 Hz, 2H) ppm; ^{13}C NMR (125 MHz, $CDCl_3$) δ 180.5 (q, $J_{C\text{-}F}$ = 35 Hz), 135.5, 132.5, 124.6, 121.2, 117.3 (q, $J_{C\text{-}CF3}$ = 279 Hz) ppm; ^{19}F NMR (470 MHz, $CDCl_3$) δ –69.8 (s, CF_3) ppm; HRMS (EI) (*m/z*) cald. for $C_8H_5BrF_3NS$, 282.9278; found, 282.9281.

The spectral data were identical to those reported in the literature[18].

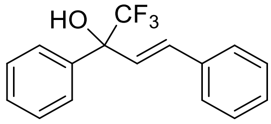

(*E*)-1,1,1-Trifluoro-2,4-diphenylbut-3-en-2-ol (1r)

The product **1r** was obtained in 91% isolated yield (76.1 mg) as a colorless oil.

^{1}H NMR (500 MHz, $CDCl_3$) δ 7.64 (m, 2H), 7.44–7.38 (m, 5H), 7.37–7.33 (m, 2H), 7.31–7.28 (m, 1H), 6.88 (d, J = 16.1 Hz, 1H), 6.72 (d, J = 16.1 Hz, 1H) ppm; ^{13}C NMR (125 MHz, $CDCl_3$) δ 137.4, 135.5, 135.6, 128.9, 128.8, 128.7, 128.4, 127.0, 126.9, 126.5, 125.1 (q, $J_{C\text{-}F}$ = 284 Hz), 77.3 (q, $J_{C\text{-}CF3}$ = 29 Hz) ppm; ^{19}F NMR (470 MHz, $CDCl_3$) δ –78.6 (s, CF_3) ppm; HRMS (EI) (*m/z*) cald. for $C_{16}H_{13}F_3O$, 278.0918; found, 278.0921.

The spectral data were identical to those reported in the literature[18].

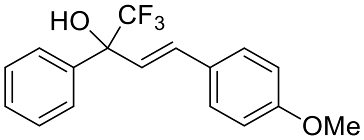

(*E*)-1,1,1-Trifluoro-4-(4-methoxyphenyl)-2-phenylbut-3-en-2-ol (1s)

The product **1s** was obtained in 94% isolated yield (86.9 mg) as a yellow oil.

^{1}H NMR (500 MHz, $CDCl_3$) δ 7.64 (m, 2H), 7.42–7.34 (m, 5H), 6.88–6.86 (m, 2H), 6.79 (d, J = 16.1 Hz, 1H), 6.58 (d, J = 16.1 Hz, 1H), 3.81 (s, 3H) ppm; ^{13}C NMR (125 MHz, $CDCl_3$) δ 160.0, 137.6, 133.2, 128.8, 128.3, 128.2, 126.9, 125.1 (q, $J_{C\text{-}F}$ = 284 Hz), 124.2, 114.2, 77.3 (q, $J_{C\text{-}CF3}$ = 29 Hz), 55.4 ppm; ^{19}F NMR (470 MHz, $CDCl_3$) δ –78.6 (s, CF_3) ppm; HRMS (EI) (*m/z*) cald. for $C_{17}H_{15}F_3O_2$, 308.1024; found, 308.1027.

The spectral data were identical to those reported in the literature[19].

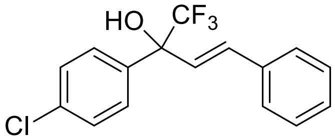

(*E*)-2-(4-Chlorophenyl)-1,1,1-trifluoro-4-phenylbut-3-en-2-ol (1t)

The product **1t** was obtained in 85% isolated yield (79.4 mg) as a colorless oil.

^{1}H NMR (500 MHz, $CDCl_3$) δ 7.59–7.57 (m, 2H), 7.43–7.31 (m, 7H), 7.57–7.55 (m, 2H), 7.45–7.40 (m, 3H), 6.85 (d, J = 16.1 Hz, 1H), 6.69 (d, J = 16.1 Hz, 1H), 2.66 (s, 1H) ppm; ^{13}C NMR (125 MHz, $CDCl_3$) δ 135.8, 135.3, 135.0, 134.2, 128.9, 128.8, 128.6, 128.5, 127.0, 126.02, 124.9 (q, $J_{C\text{-}F}$ = 284 Hz), 77.1 (q, $J_{C\text{-}CF3}$ = 29 Hz) ppm; ^{19}F NMR (470 MHz, $CDCl_3$) δ –78.7 (s, CF_3) ppm; HRMS (EI) (*m/z*) cald. for $C_{16}H_{12}ClF_3O$, 312.0529; found, 312.0530.

The spectral data were identical to those reported in the literature[20].

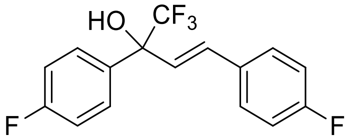

(*E*)-1,1,1-Trifluoro-2,4-bis(4-fluorophenyl)but-3-en-2-ol (1u)

The product **1u** was obtained in 78% isolated yield (73.5 mg) as a yellow oil.

^{1}H NMR (500 MHz, $CDCl_3$) δ 7.61 (dd, *J* = 8.6, 5.4 Hz, 2H), 7.40 (dd, *J* = 8.6, 5.4 Hz, 2H), 7.09 (t, *J* = 8.7 Hz, 2H), 7.04 (t, *J* = 8.6 Hz, 2H), 6.82 (d, *J* = 16.1 Hz, 1H), 6.69 (d, *J* = 16.1 Hz, 1H), 2.65 (s, 1H) ppm; ^{13}C NMR (125 MHz, $CDCl_3$) δ 164.0 (d, *J* = 6.1 Hz), 161.9 (d, *J* = 5.5 Hz), 133.0 (d, *J* = 3.4 Hz), 132.8, 131.5 (d, *J* = 3.3 Hz), 128.9 (d, *J* = 8.1 Hz), 128.6 (d, *J* = 8.1 Hz), 126.0, 124.9 (q, *J* = 284 Hz), 115.8 (d, *J* = 22 Hz), 115.3 (d, *J* = 22 Hz), 76.9 (q, *J* = 29 Hz) ppm; ^{19}F NMR (470 MHz, $CDCl_3$) δ –78.8 (s, CF_3), –112.4 (s, Ar-F), –112.9 (s, Ar-F) ppm; HRMS (EI) (*m/z*) cald. for $C_{16}H_{11}F_5O$, 314.0730; found, 314.0727.

The spectral data were identical to those reported in the literature[10].

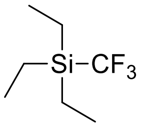

Triethyl(trifluoromethyl)silane (1v)

The product **1x** was obtained in 79% isolated yield (43.7 mg) as a colorless oil.

^{1}H NMR (500 MHz, $CDCl_3$) δ 1.04 (t, *J* = 8.0 Hz, 9H), 0.78 (q, *J* = 8.0 Hz, 6H) ppm; ^{13}C NMR (125 MHz, $CDCl_3$) δ 132.1 (q, $J_{C\text{-}F}$ = 321 Hz), 6.5, 0.8 ppm; ^{19}F NMR (470 MHz, $CDCl_3$) δ –61.3 (s, CF_3) ppm.

The spectral data were identical to those reported in the literature[10].

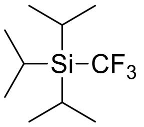

Triisopropyl(trifluoromethyl)silane (1w)

The product **1y** was obtained in 87% isolated yield (59.1 mg) as a colorless oil.

^{1}H NMR (500 MHz, $CDCl_3$) δ 1.33–1.14 (m, 3H), 1.14 (d, *J* = 7.5 Hz, 18H) ppm; ^{13}C NMR (125 MHz, $CDCl_3$) δ 132.5 (q, $J_{C\text{-}F}$ = 324 Hz), 17.9, 9.5 ppm; ^{19}F NMR (470 MHz, $CDCl_3$) δ –55.5 (s, CF_3) ppm; HRMS (EI) (*m/z*) cald. for $C_{10}H_{21}F_3Si$, 226.1365; found, 226.1363.

The spectral data were identical to those reported in the literature[10].

CF_3–BF_3K

Potassium trifluoro(trifluoromethyl)borate (1x)

The product **1z** was obtained in 80% isolated yield (42.2 mg) as a colorless solid. The resulting solution was added to a solution of KHF_2 (6 equiv) in glacial acetic acid. The reaction mixture was stirred at –50 °C for 1 h and then for 3 h at 80 °C. All volatiles were removed under vacuum and the residue was diluted with acetone and neutralized by the addition of K_2CO_3. The precipitate was filtered off. The filtrate was concentrated and the addition of CH_2Cl_2 for 60 min of operation time. ^{19}F NMR (470 MHz, D_2O) δ –76.7 (q, J = 34 Hz, CF_3), 155.1 (q, J = 41 Hz, 3F) ppm; ^{11}B NMR (160 MHz, D_2O) δ –1.33 (m, 1B) ppm.
The spectral data were identical to those reported in the literature[10].

Chemoselective reaction with multi-functionalized electrophilic substrates

For the synthesis of compounds **3a**–**3e**, the reactions were carried out using 1,4-cyclohexanedione, 1,4-benzoquinone, 4-formylbenzonitrile, methyl 4-formylbenzoate and terephthalaldehyde as electrophiles, respectively in GLD-2. For isolation of the desired products, the organic layer of the product solution was separated and the aqueous layer was extracted with Et_2O (15 mL×3). The combined organic layer was dried over Na_2SO_4 and concentrated. The crude product was purified by column chromatography.

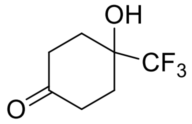

4-Hydroxy-4-(trifluoromethyl)cyclohexanone (3a)

The product **3a** was obtained in 75% isolated yield (41 mg) as an off-white solid.
^{1}H NMR (500 MHz, $CDCl_3$) δ 2.73 (ddd, J = 6.6, 6.3, 6.5 Hz, 2H), 2.51 (s, 1H), 2.41-2.37 (m, 2H), 2.20–2.16 (m, 2H), 2.09 (ddd, J = 5.1, 5.0, 5.1 Hz, 2H) ppm; ^{13}C NMR (125 MHz, $CDCl_3$) δ 209.4, 126.0 (q, $J_{C\text{-}F}$ = 283 Hz), 71.6 (q, $J_{C\text{-}CF3}$ = 29 Hz), 35.2, 29.9 ppm; ^{19}F NMR (470 MHz, $CDCl_3$) δ –83.8 (s, CF_3) ppm; HRMS (EI) (*m/z*) cald. for $C_7H_9F_3O_2$, 182.0555; found, 182.0552.
The spectral data were identical to those reported in the literature[21].

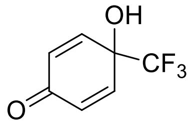

4-Hydroxy-4-(trifluoromethyl)cyclohexa-2,5-dienone (3b)

The product **3b** was obtained in 71% isolated yield (37.9 mg) as a white solid.
^{1}H NMR (500 MHz, $CDCl_3$) δ 6.88 (d, J = 10.5 Hz, 2H), 6.43 (d, J = 10.0 Hz, 2H), 3.29 (br s, 1H) ppm; ^{13}C NMR (125 MHz, $CDCl_3$) δ 184.1, 141.0, 132.2, 123.4 (q, $J_{C\text{-}F}$ = 285 Hz), 69.6 (q, $J_{C\text{-}CF3}$ = 31

Hz) ppm; ^{19}F NMR (470 MHz, $CDCl_3$) δ –79.7 (s, CF_3) ppm; HRMS (EI) (*m/z*) cald. for $C_7H_5F_3O_2$, 178.0242; found, 178.0243.

The spectral data were identical to those reported in the literature[22].

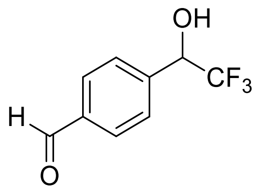

4-(2,2,2-Trifluoro-1-hydroxyethyl)benzaldehyde (3c)

The product **3c** was obtained in 54% isolated yield (33.1 mg) as a white solid.

^{1}H NMR (500 MHz, $CDCl_3$) δ 7.92 (d, J = 8.5 Hz, 2H), 7.68 (d, J = 8.0 Hz, 2H), 5.15 (q, J = 6.5 Hz, 1H), 3.10 (s, 3H) ppm; ^{13}C NMR (125 MHz, $CDCl_3$) δ 192.0, 140.3, 137.0, 129.9, 128.2, 123.9 (q, $J_{C\text{-}F}$ = 281 Hz), 72.3 (q, $J_{C\text{-}CF3}$ = 31 Hz) ppm; ^{19}F NMR (470 MHz, $CDCl_3$) δ –78.1 (d, $J_{F\text{-}H}$= 6.5 Hz) ppm; HRMS (EI) (*m/z*) cald. for $C_9H_7F_3O_2$, 204.0398; found, 234.0396.

The spectral data were identical to those reported in the literature[23].

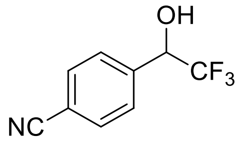

4-(2,2,2-Trifluoro-1-hydroxyethyl)benzonitrile (3d)

The product **3d** was obtained in 69% isolated yield (41.6 mg) as a white solid.

^{1}H NMR (500 MHz, $CDCl_3$) δ 7.74 (d, J = 8.5 Hz, 2H), 7.65 (d, J = 8.1 Hz, 2H), 5.14 (q, J = 6.5 Hz, 1H), 2.89 (br s, 1H) ppm; ^{13}C NMR (125 MHz, $CDCl_3$) δ 139.1, 132.3, 128.3, 123.8 (q, $J_{C\text{-}F}$ = 281 Hz), 118.3, 113.2, 71.9 (q, $J_{C\text{-}CF3}$ = 32 Hz) ppm; ^{19}F NMR (470 MHz, $CDCl_3$) δ –78.2 (d, $J_{F\text{-}H}$ = 6.7 Hz) ppm; HRMS (EI) (*m/z*) cald. for $C_9H_6F_3NO$, 201.0401; found, 201.0403.

The spectral data were identical to those reported in the literature[14].

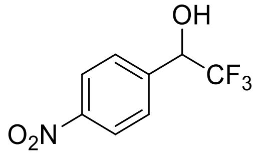

2,2,2-Trifluoro-1-(4-nitrophenyl)ethanol (3e)

The product **1j** was obtained in 55% isolated yield (36.5 mg) as an off-white solid.

^{1}H NMR (500 MHz, $CDCl_3$) δ 8.30 (d, J = 8.9 Hz, 2H), 7.72 (d, J = 8.9 Hz, 2H), 5.23–5.18 (m, 1H), 2.81 (d, J = 4.4 Hz, 1H) ppm; ^{13}C NMR (125 MHz, $CDCl_3$) δ 148.6, 140.4, 128.5, 123.7, 123.6 (q, $J_{C\text{-}}$

$_{F}$ = 281 Hz), 71.8 (q, $J_{C\text{-}CF3}$ = 32 Hz) ppm; ^{19}F NMR (470 MHz, $CDCl_3$) δ –78.2 (d, $J_{F\text{-}H}$ = 5.9 Hz) ppm; HRMS (EI) (*m/z*) cald. for $C_8H_6F_3NO_3$, 221.0300; found, 221.0303.
The spectral data were identical to those reported in the literature[14].

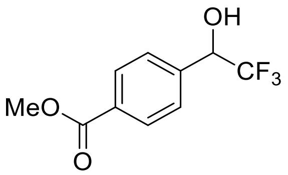

Methyl 4-(2,2,2-trifluoro-1-hydroxyethyl)benzoate (3f)

The product **3e** was obtained in 63% isolated yield (44.3 mg) as a colorless oil.
^{1}H NMR (500 MHz, $CDCl_3$) δ 8.03 (d, *J* = 8.5 Hz, 2H), 7.55 (d, *J* = 8.0 Hz, 2H), 5.08 (q, *J* = 7.0 Hz, 1H), 3.91 (s, 3H), 3.22 (br s, 1H) ppm; ^{13}C NMR (125 MHz, $CDCl_3$) δ 166.9, 139.0, 131.0, 129.7, 127.5, 124.1 (q, $J_{C\text{-}F}$ = 281 Hz), 72.3 (q, $J_{C\text{-}CF3}$ = 31 Hz), 52.4 ppm; ^{19}F NMR (470 MHz, $CDCl_3$) δ –78.1 (d, $J_{F\text{-}H}$ = 6.5 Hz) ppm; HRMS (EI) (*m/z*) cald. for $C_{10}H_9F_3O_3$, 234.0504; found, 234.0501.
The spectral data were identical to those reported in the literature[11].

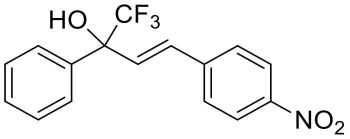

(*E*)-1,1,1-Trifluoro-4-(4-nitrophenyl)-2-phenylbut-3-en-2-ol (3g)

The product **3g** was obtained in 73% isolated yield (70.8 mg) as a yellow oil.
^{1}H NMR (500 MHz, $CDCl_3$) δ 8.19 (m, 2H), 7.65–7.63 (m, 2H), 7.57–7.55 (m, 2H), 7.45–7.40 (m, 3H), 6.89 (d, *J* = 16.0 Hz, 1H), 6.85 (d, *J* = 16.0 Hz, 1H), 3.10 (s, 1H) ppm; ^{13}C NMR (125 MHz, $CDCl_3$) δ 147.5, 141.9, 136.8, 131.2, 130.9, 129.1, 128.6, 127.6, 124.8 (q, $J_{C\text{-}F}$ = 284 Hz), 124.1, 114.2, 77.3 (q, $J_{C\text{-}CF3}$ = 29 Hz) ppm; ^{19}F NMR (470 MHz, $CDCl_3$) δ –78.1 (s, CF_3) ppm; HRMS (EI) (*m/z*) cald. for $C_{16}H_{12}F_3NO_3$, 323.0769; found, 323.0768.

7. Continuous flow mass production through GLD-2

For the synthesis of compounds **1v**, **1w,** and **1x**, the reactions were carried out using chlorotriethylsilane, chlorotriisopropylsilane, and trimethyl borate as an electrophile, respectively. In the case of using chlorotrethylsilane and chlorotriisopropylsilane, for isolation of the desired products, the organic layer of the product solution was separated and the aqueous layer was extracted with Et_2O and was further washed with cold concentrated sulfuric acid (98%) to remove most of the siloxane and silanols formed

in the reaction. The organic layer was then washed with water until the pH of the water showed neutral and dried over Na_2SO_4. The solvent was removed under vacuum to obtain a crude product which was distilled under vacuum[7].

In the case of using trimethyl borate, the resulting solution was added to a solution of KHF_2 (6 equivalent) in glacial acetic acid. The reaction mixture was stirred at –50 °C for 1 h and then for 3 h at 80 °C. All volatiles were removed under vacuum and the residue was diluted with acetone and neutralized by the addition of K_2CO_3. The precipitate was filtered off. The filtrate was concentrated and the addition of CH_2Cl_2[24].

8. ^{1}H, ^{13}C, ^{19}F, and ^{11}B NMR Spectra

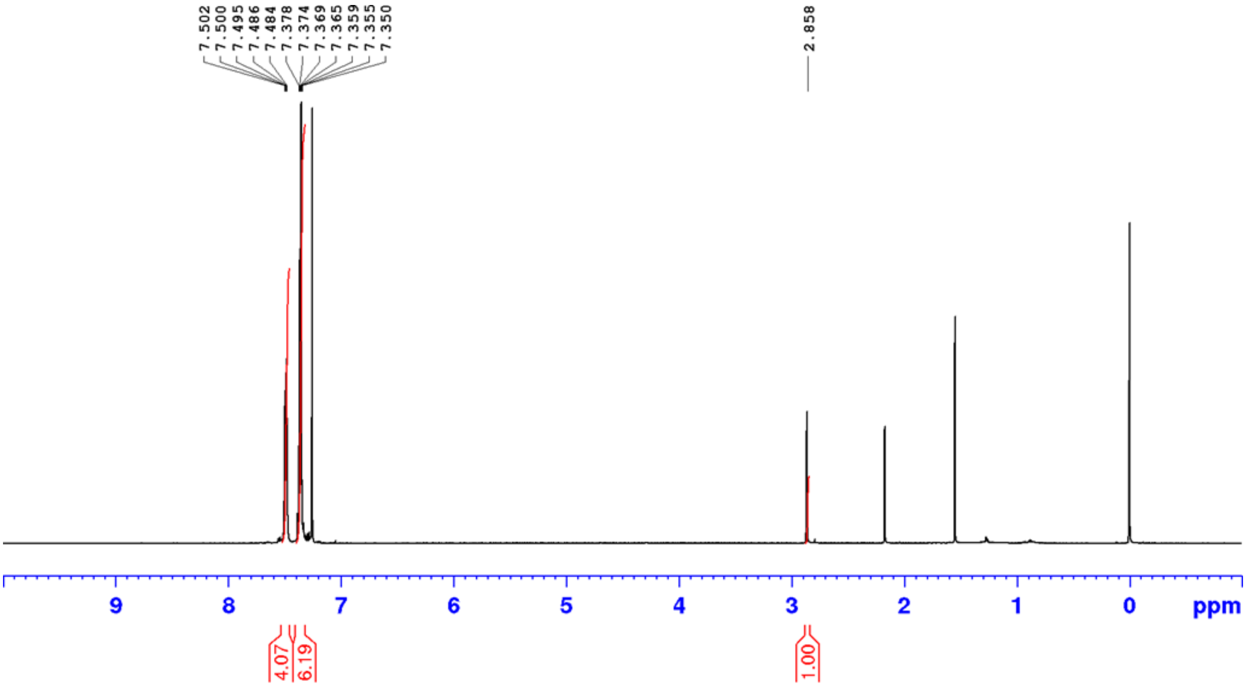

Supplementary Figure 9. ^{1}H NMR of compound 1a. The sample has been recorded in 500 MHz, $CDCl_3$ at 25 °C.

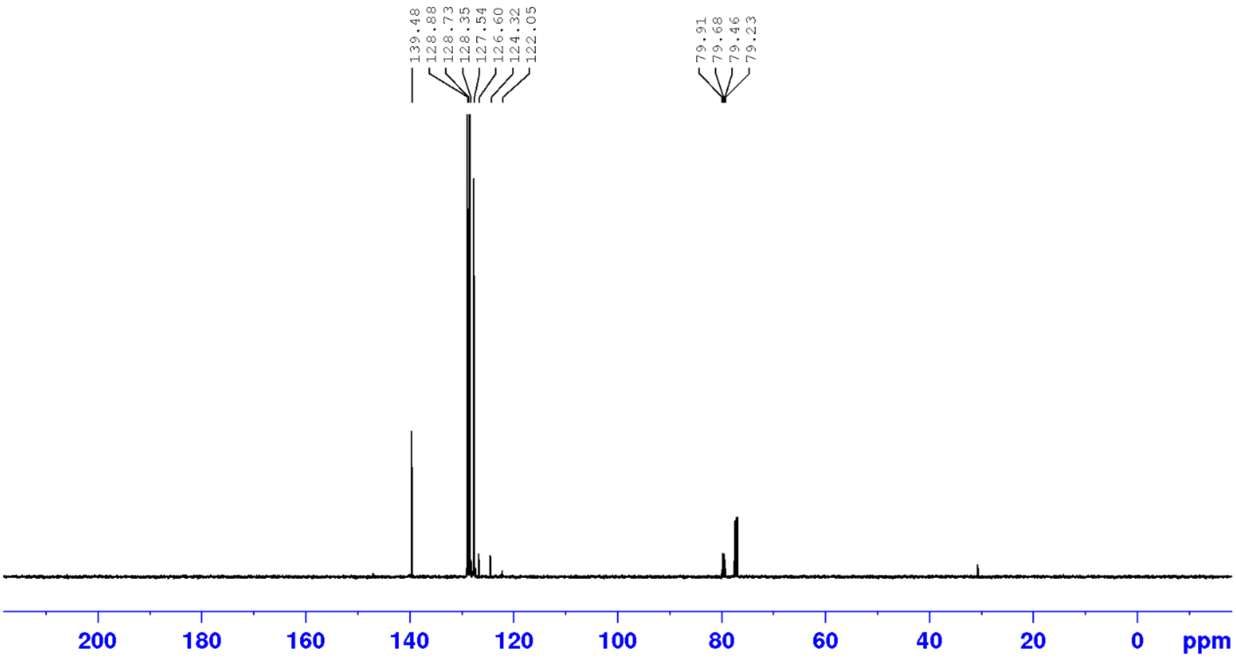

Supplementary Figure 10. ^{13}C NMR of compound 1a. The sample has been recorded in 125 MHz, $CDCl_3$ at 25 °C.

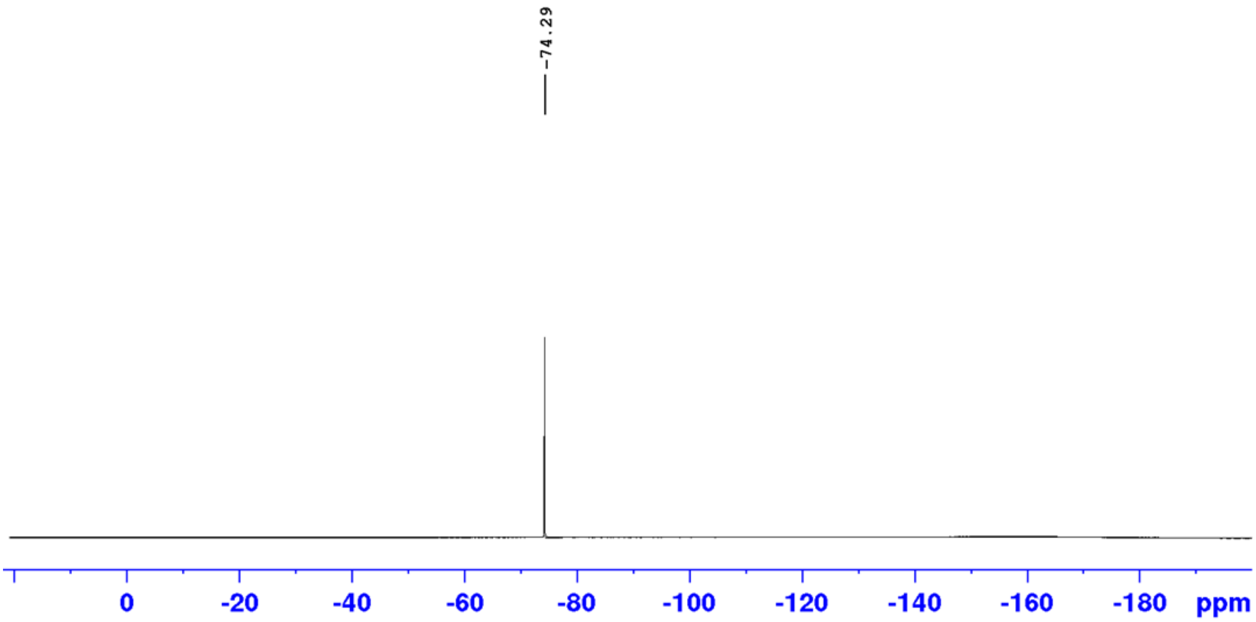

Supplementary Figure 11. ^{19}F NMR of compound 1a. The sample has been recorded in 470 MHz, $CDCl_3$ at 25 °C.

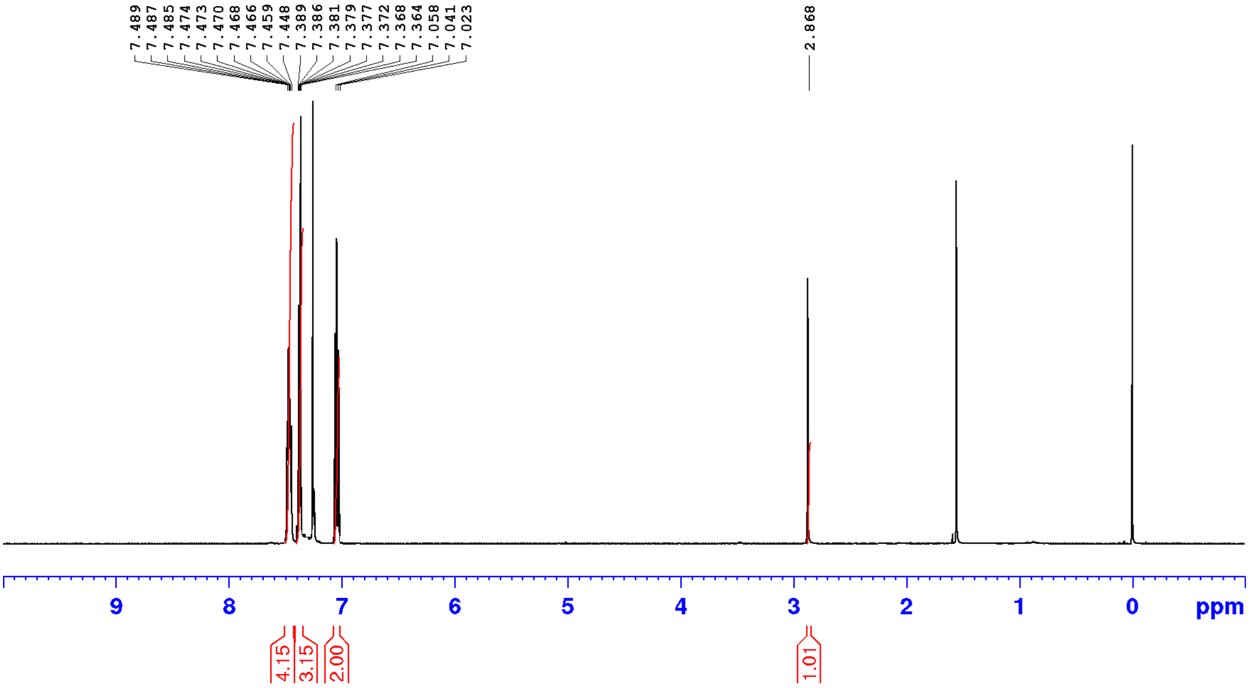

Supplementary Figure 12. ^{1}H NMR of compound 1b. The sample has been recorded in 500 MHz, $CDCl_3$ at 25 °C

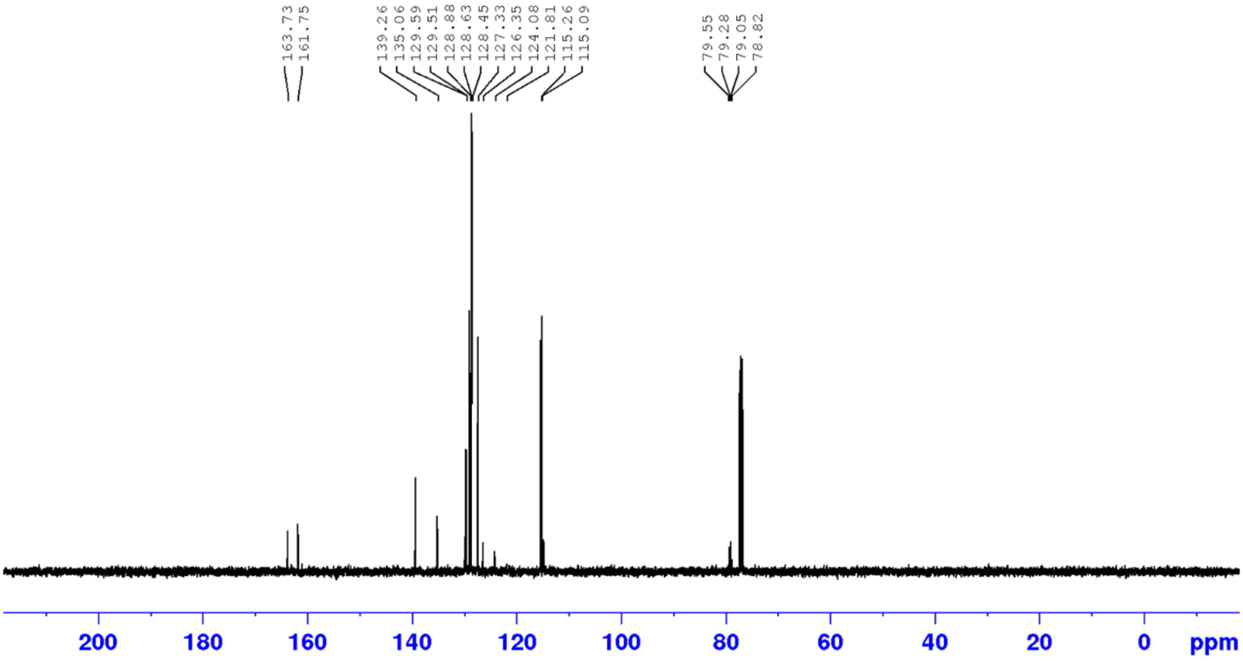

Supplementary Figure 13. ^{13}C NMR of compound 1b. The sample has been recorded in 125 MHz, $CDCl_3$ at 25 °C.

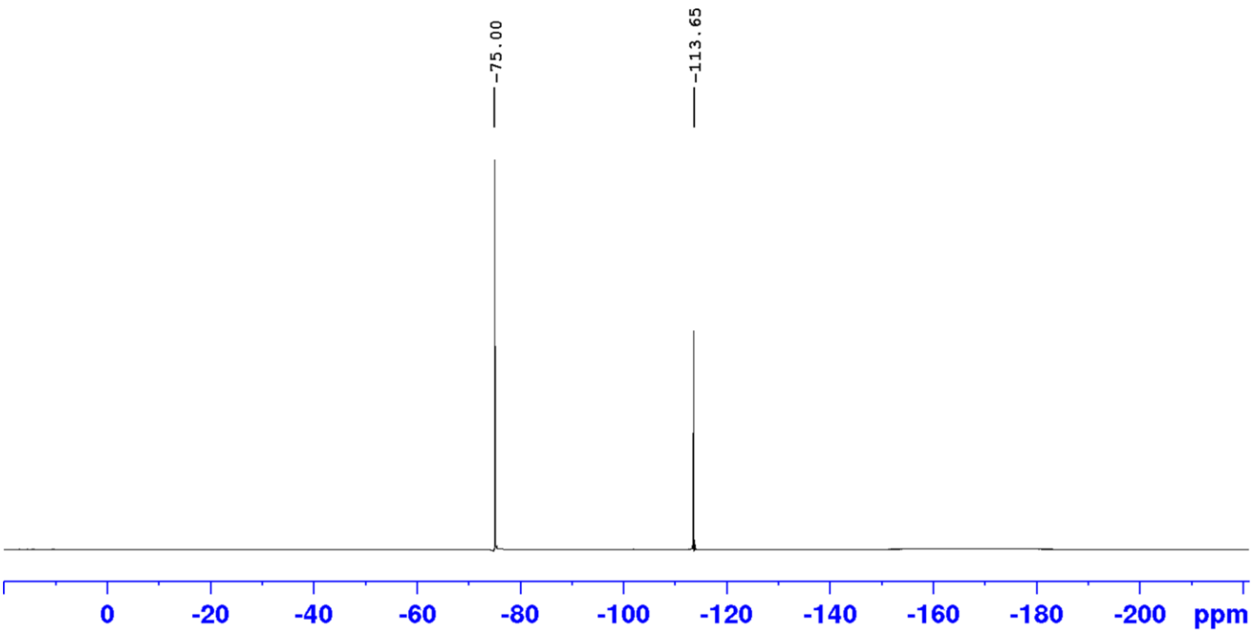

Supplementary Figure 14. ^{19}F NMR of compound 1b. The sample has been recorded in 470 MHz, $CDCl_3$ at 25 °C.

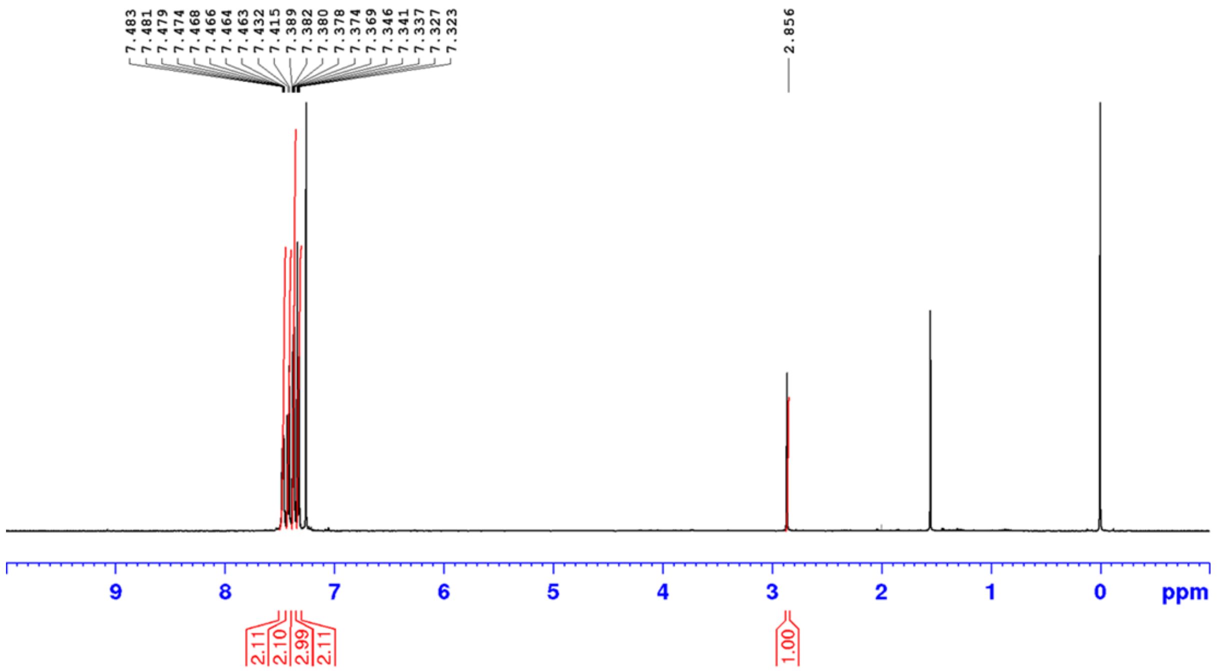

Supplementary Figure 15. ^{1}H NMR of compound 1c. The sample has been recorded in 500 MHz, $CDCl_3$ at 25 °C

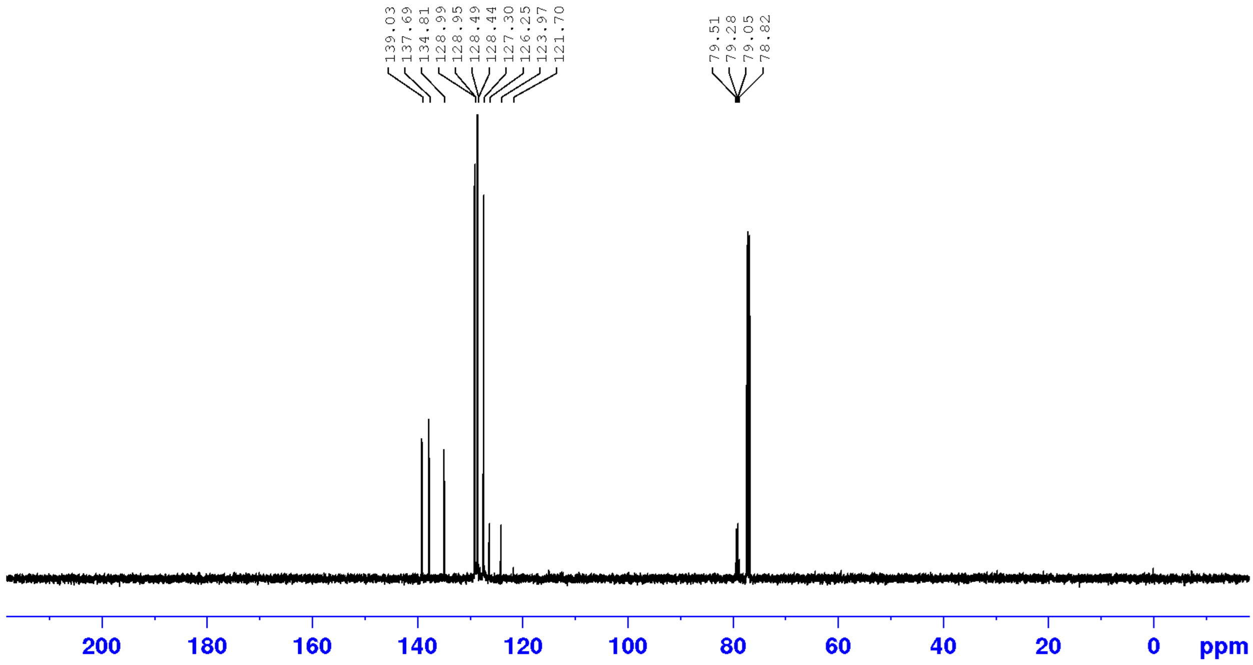

Supplementary Figure 16. ^{13}C NMR of compound 1c. The sample has been recorded in 125 MHz, $CDCl_3$ at 25 °C.

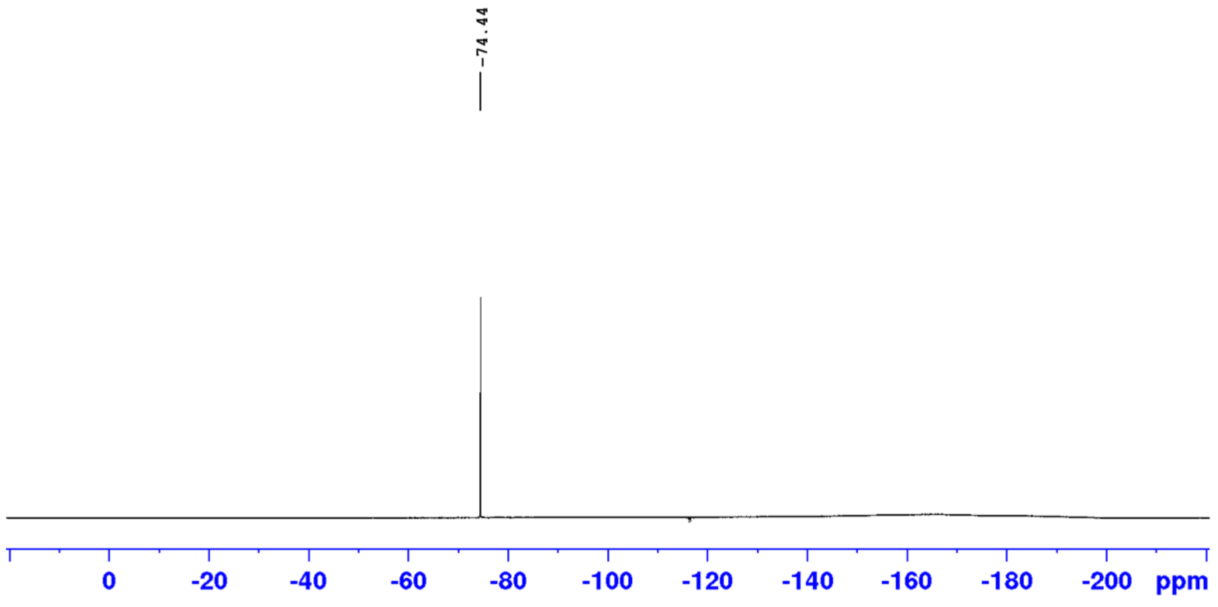

Supplementary Figure 17. ^{19}F NMR of compound 1c. The sample has been recorded in 470 MHz, $CDCl_3$ at 25 °C.

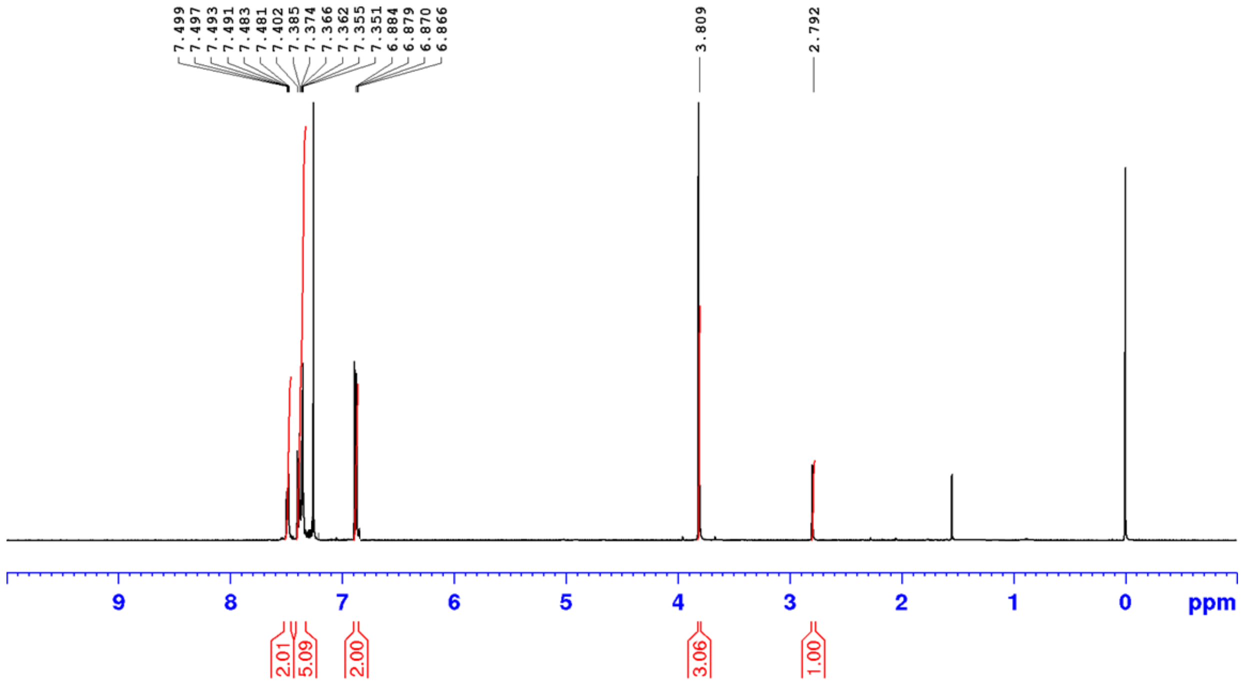

Supplementary Figure 18. ^{1}H NMR of compound 1d. The sample has been recorded in 500 MHz, $CDCl_3$ at 25 °C.

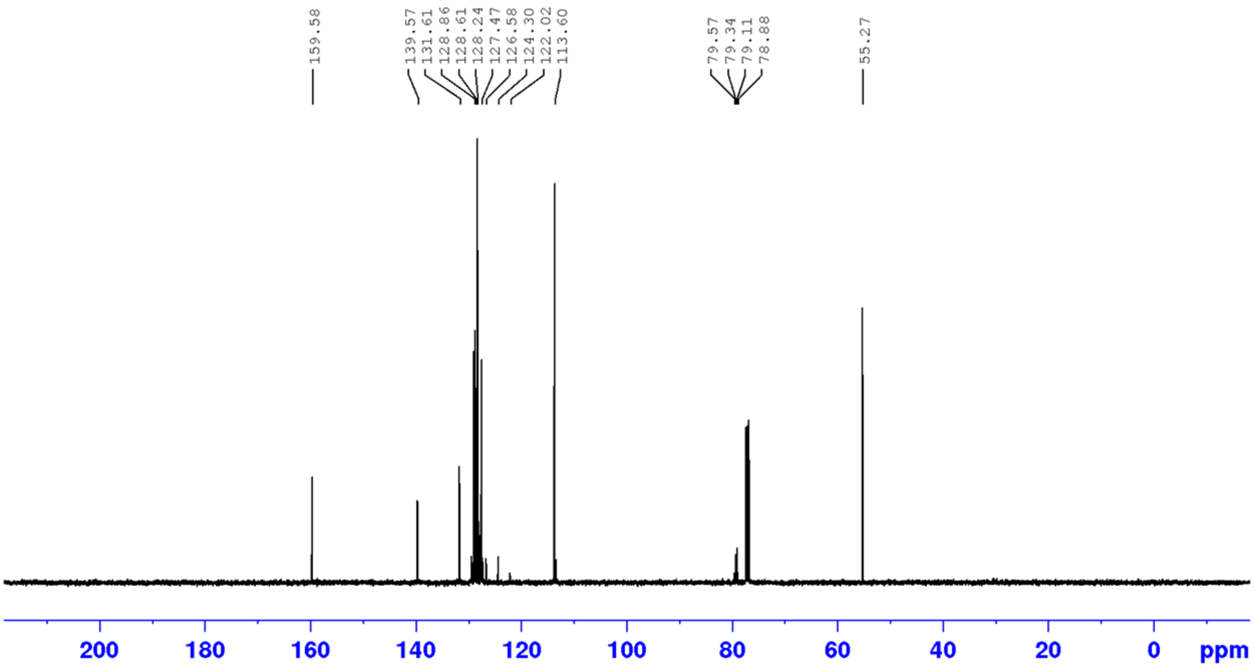

Supplementary Figure 19. ^{13}C NMR of compound 1d. The sample has been recorded in 125 MHz, $CDCl_3$ at 25 °C.

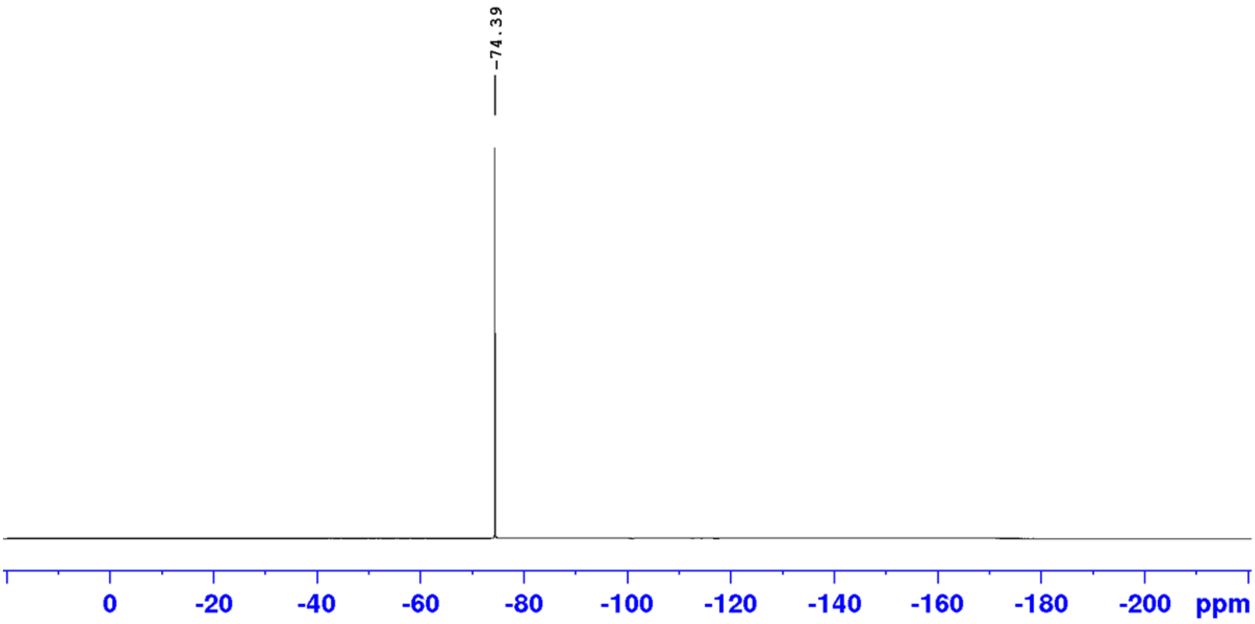

Supplementary Figure 20. ^{19}F NMR of compound 1d. The sample has been recorded in 470 MHz, $CDCl_3$ at 25 °C.

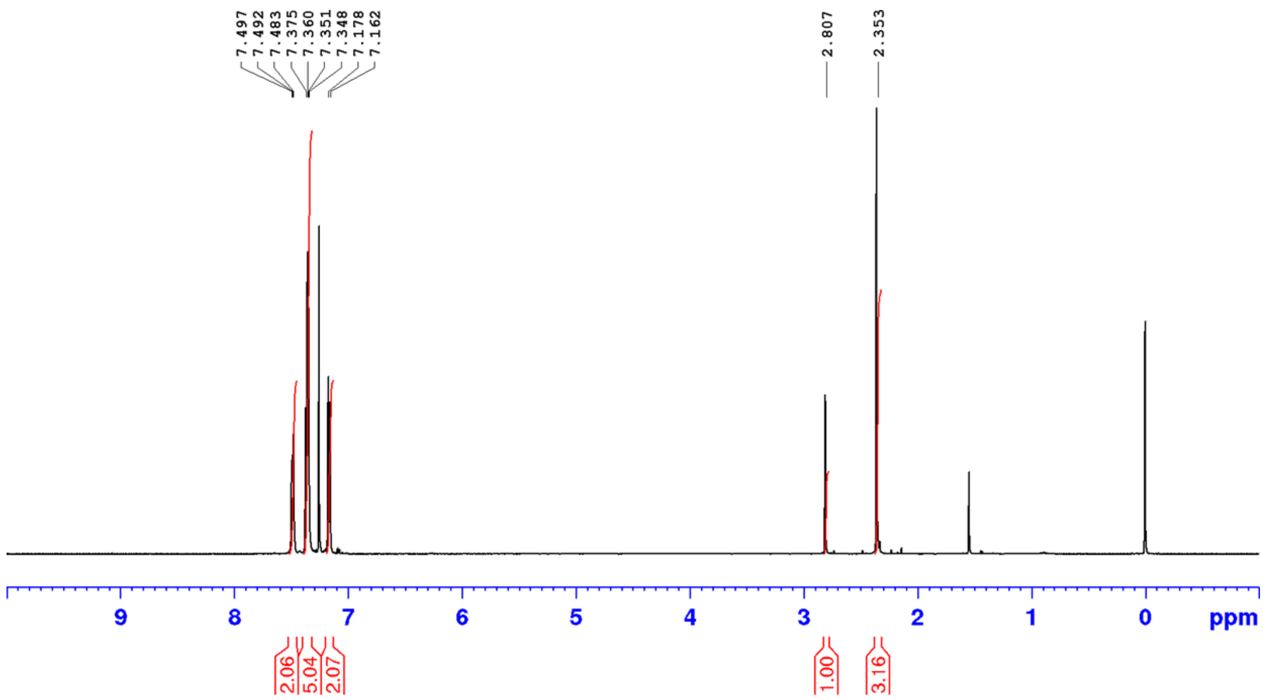

Supplementary Figure 21. ¹H NMR of compound 1e. The sample has been recorded in 500 MHz, $CDCl_3$ at 25 °C

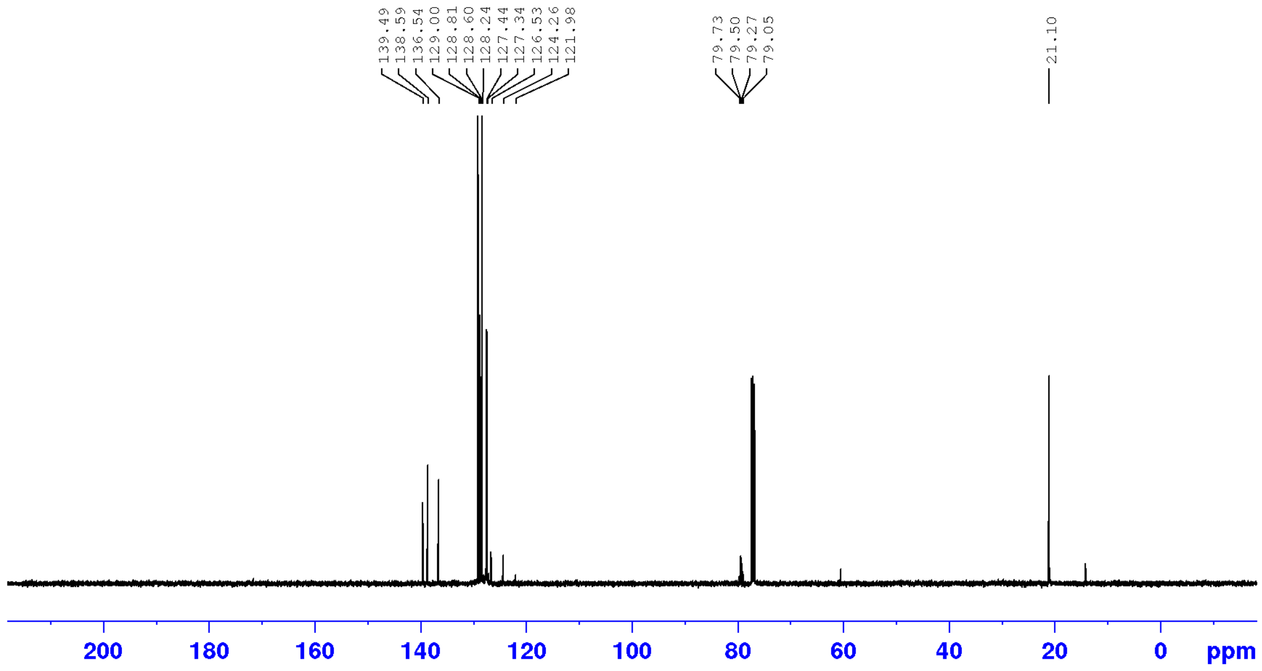

Supplementary Figure 22. ¹³C NMR of compound 1e. The sample has been recorded in 125 MHz, $CDCl_3$ at 25 °C.

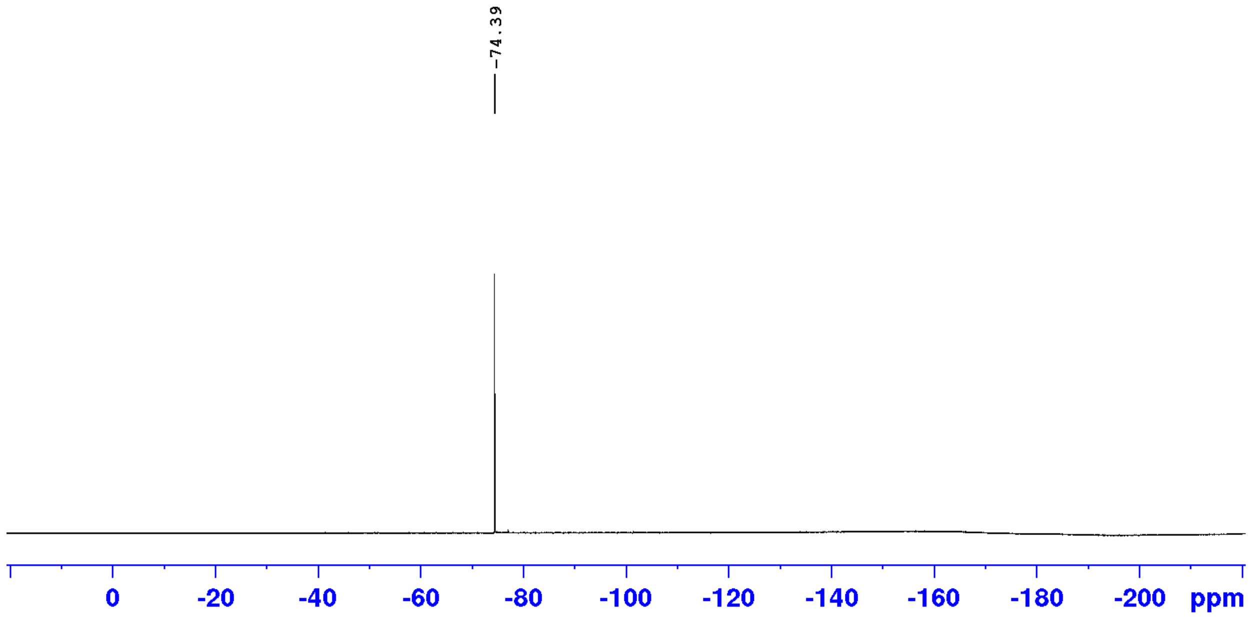

Supplementary Figure 23. ^{19}F NMR of compound 1e. The sample has been recorded in 470 MHz, $CDCl_3$ at 25 °C.

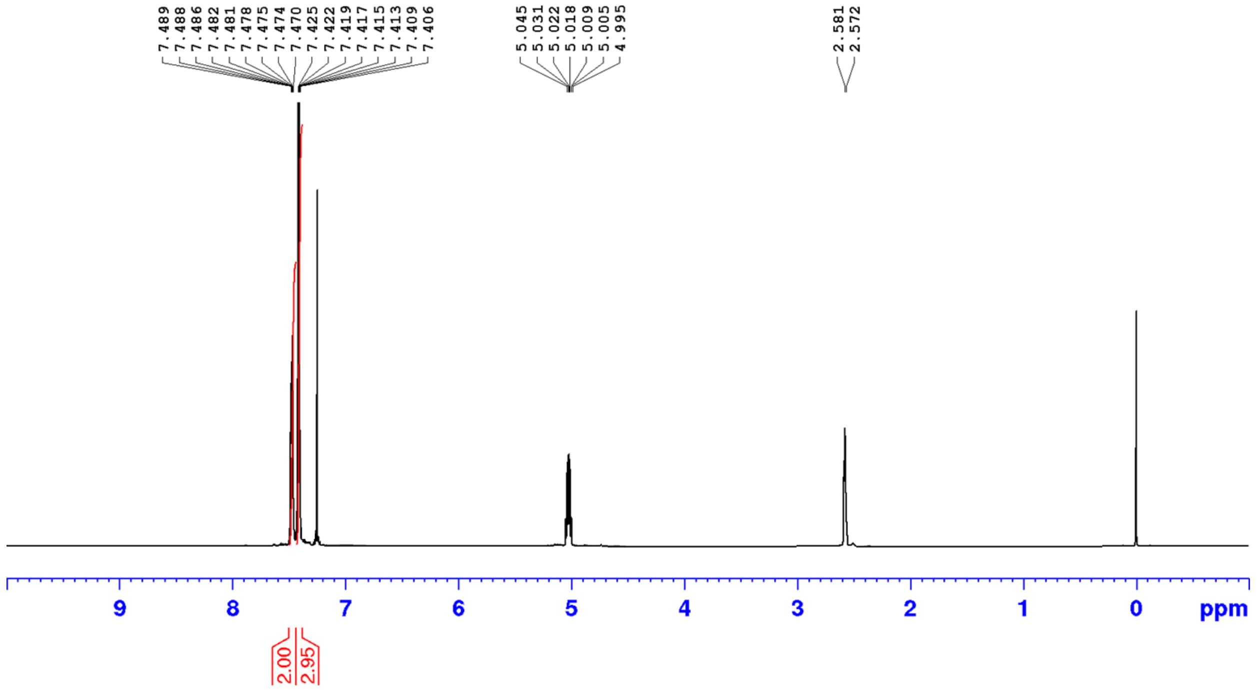

Supplementary Figure 24. ^{1}H NMR of compound 1f. The sample has been recorded in 500 MHz, $CDCl_3$ at 25 °C

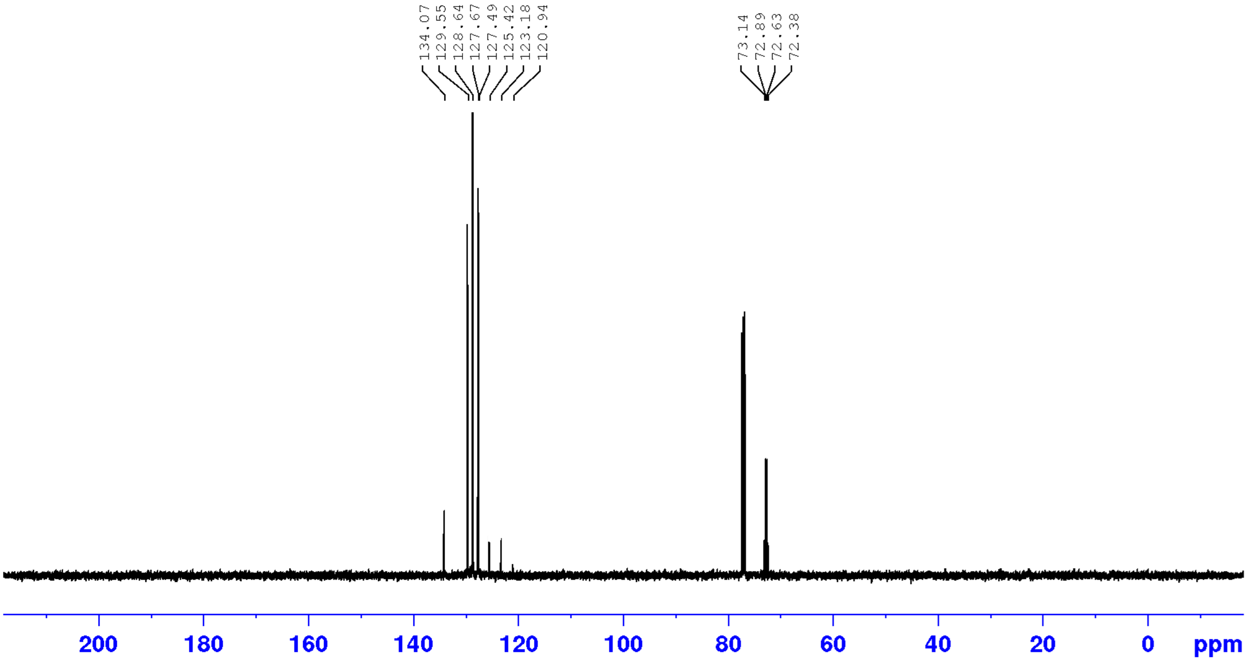

Supplementary Figure 25. ^{13}C NMR of compound 1f. The sample has been recorded in 125 MHz, $CDCl_3$ at 25 °C.

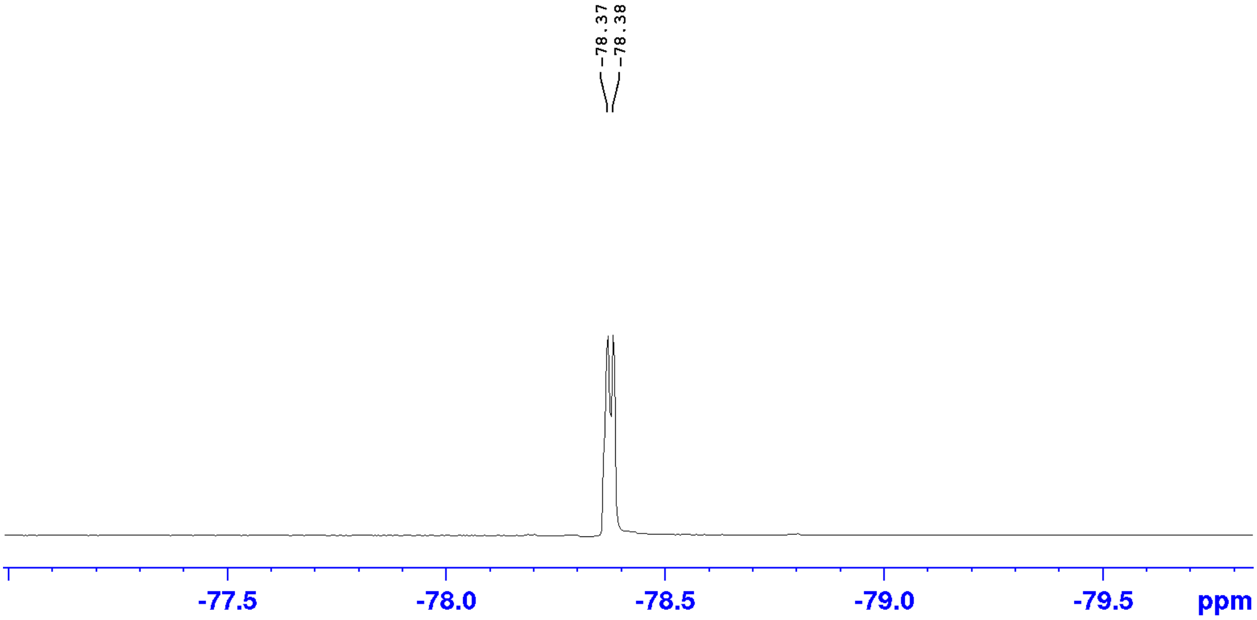

Supplementary Figure 26. ^{19}F NMR of compound 1f. The sample has been recorded in 470 MHz, $CDCl_3$ at 25 °C.

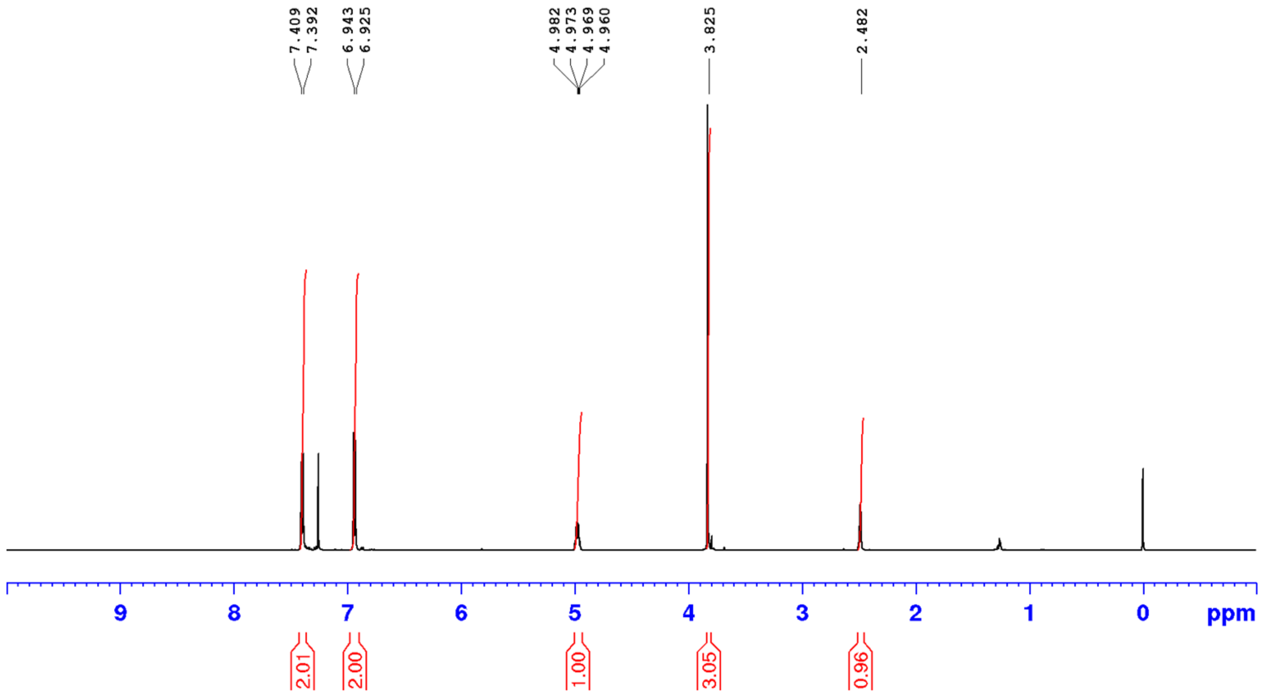

Supplementary Figure 27. ^{1}H NMR of compound 1g. The sample has been recorded in 500 MHz, $CDCl_3$ at 25 °C

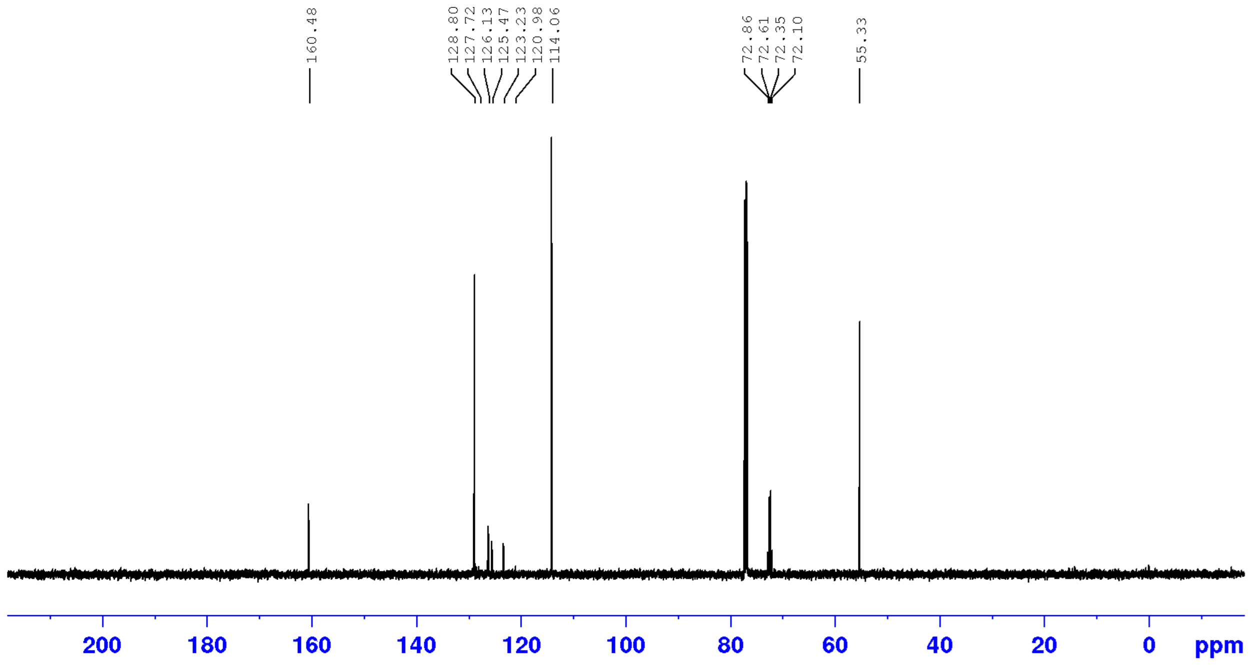

Supplementary Figure 28. ^{13}C NMR of compound 1g. The sample has been recorded in 125 MHz, $CDCl_3$ at 25 °C.

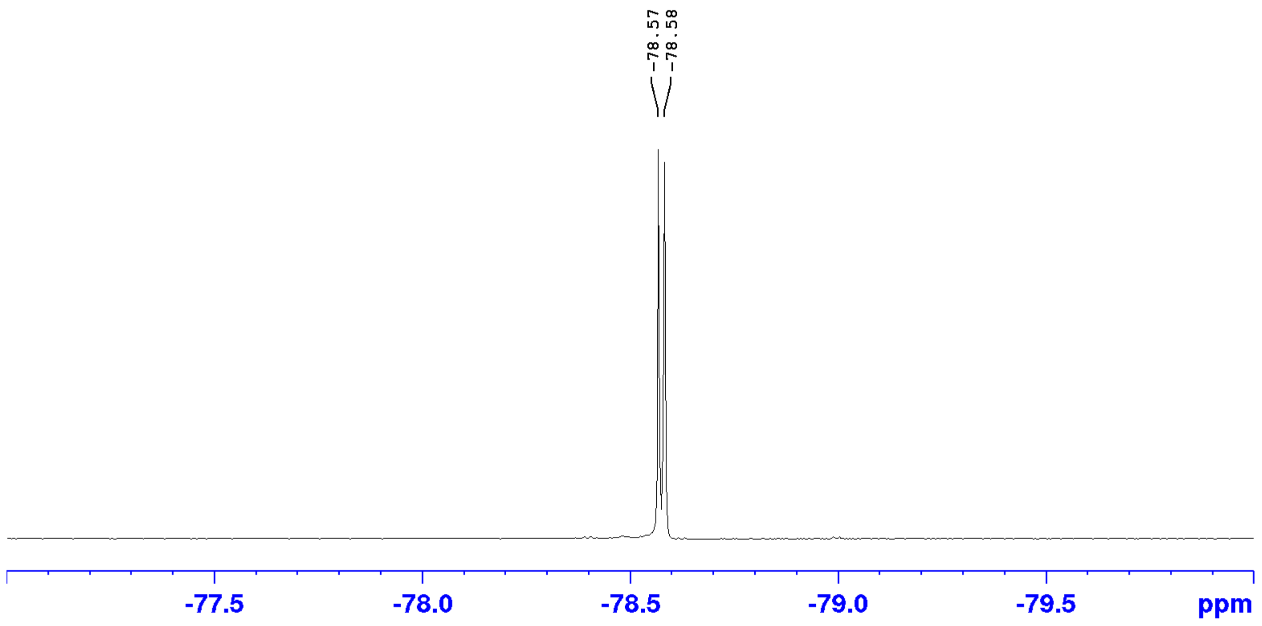

Supplementary Figure 29. ^{19}F NMR of compound 1g. The sample has been recorded in 470 MHz, $CDCl_3$ at 25 °C.

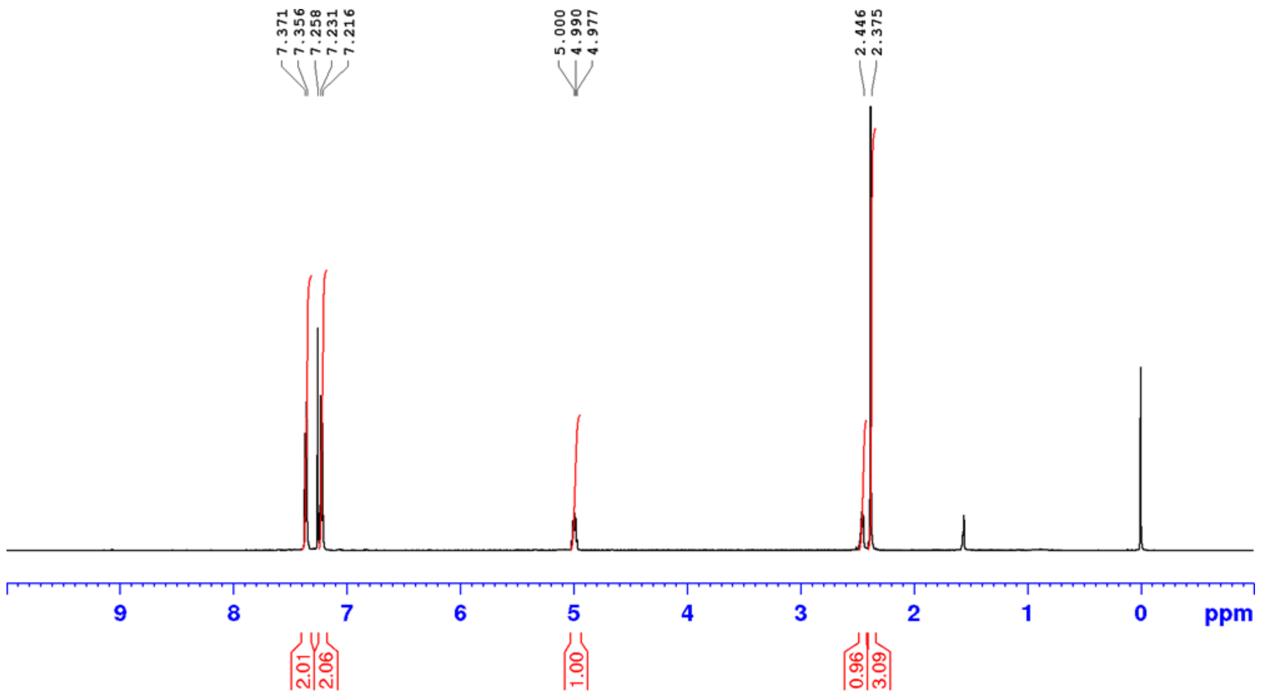

Supplementary Figure 30. ^{1}H NMR of compound 1h. The sample has been recorded in 500 MHz, $CDCl_3$ at 25 °C.

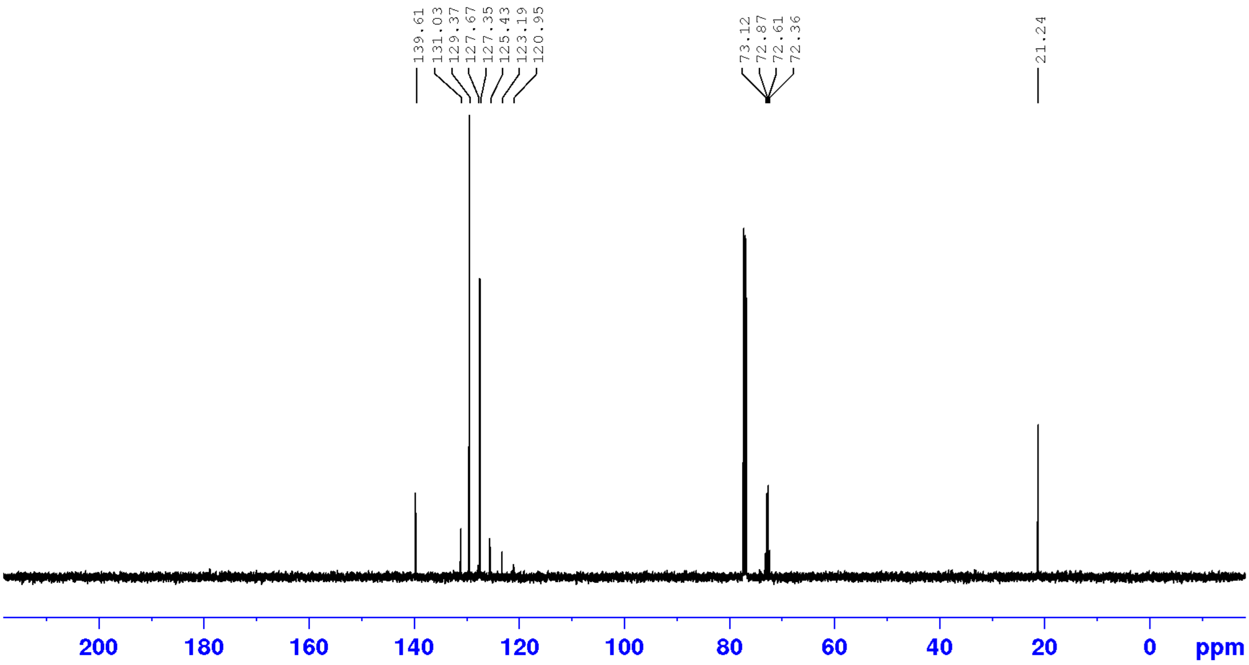

Supplementary Figure 31. ^{13}C NMR of compound 1h. The sample has been recorded in 125 MHz, $CDCl_3$ at 25 °C.

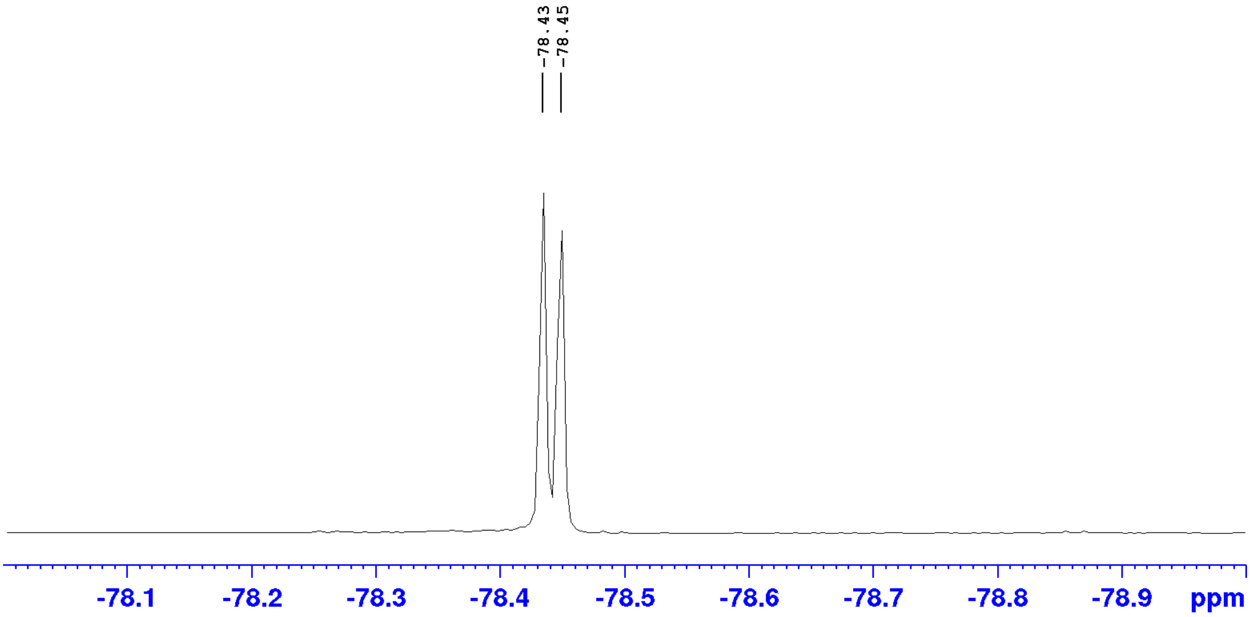

Supplementary Figure 32. ^{19}F NMR of compound 1h. The sample has been recorded in 470 MHz, $CDCl_3$ at 25 °C.

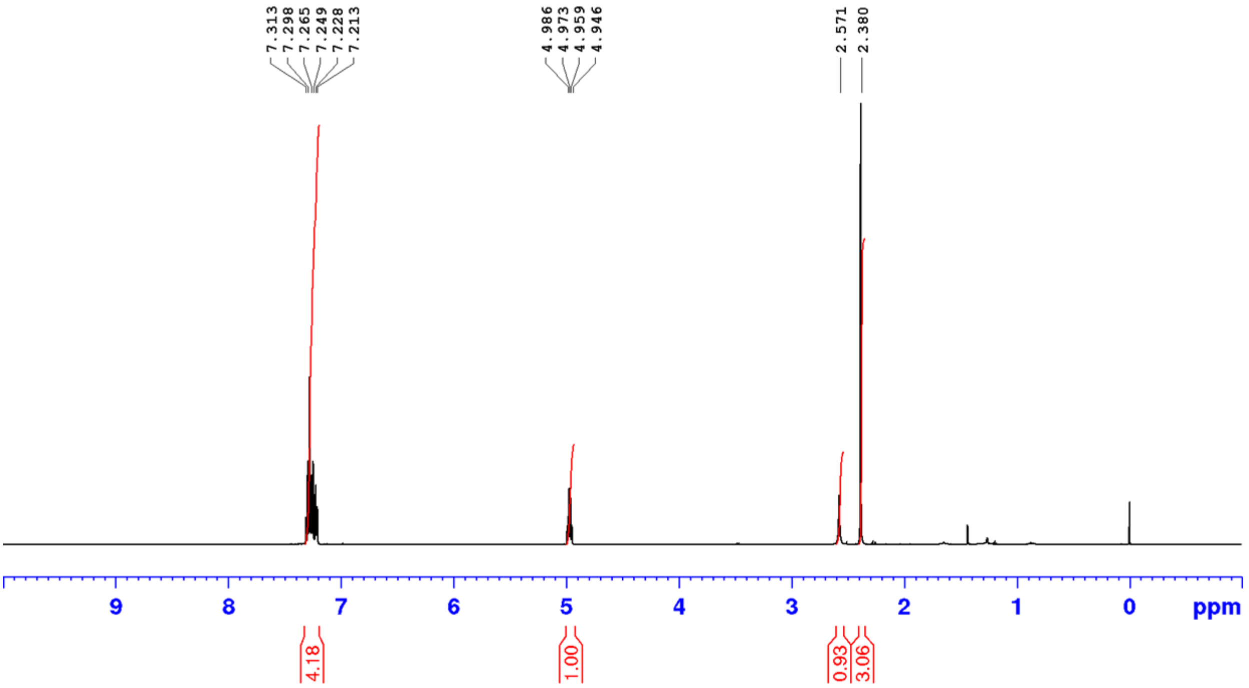

Supplementary Figure 33. ¹H NMR of compound 1i. The sample has been recorded in 500 MHz, $CDCl_3$ at 25 °C.

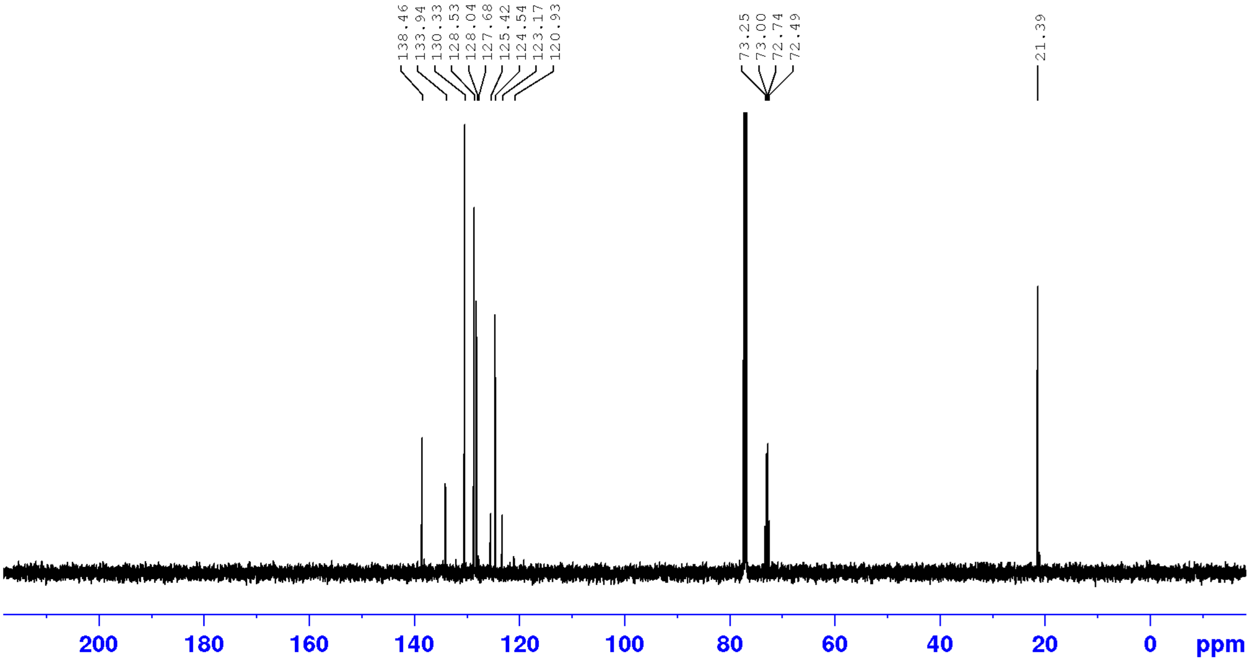

Supplementary Figure 34. ¹³C NMR of compound 1i. The sample has been recorded in 125 MHz, $CDCl_3$ at 25 °C.

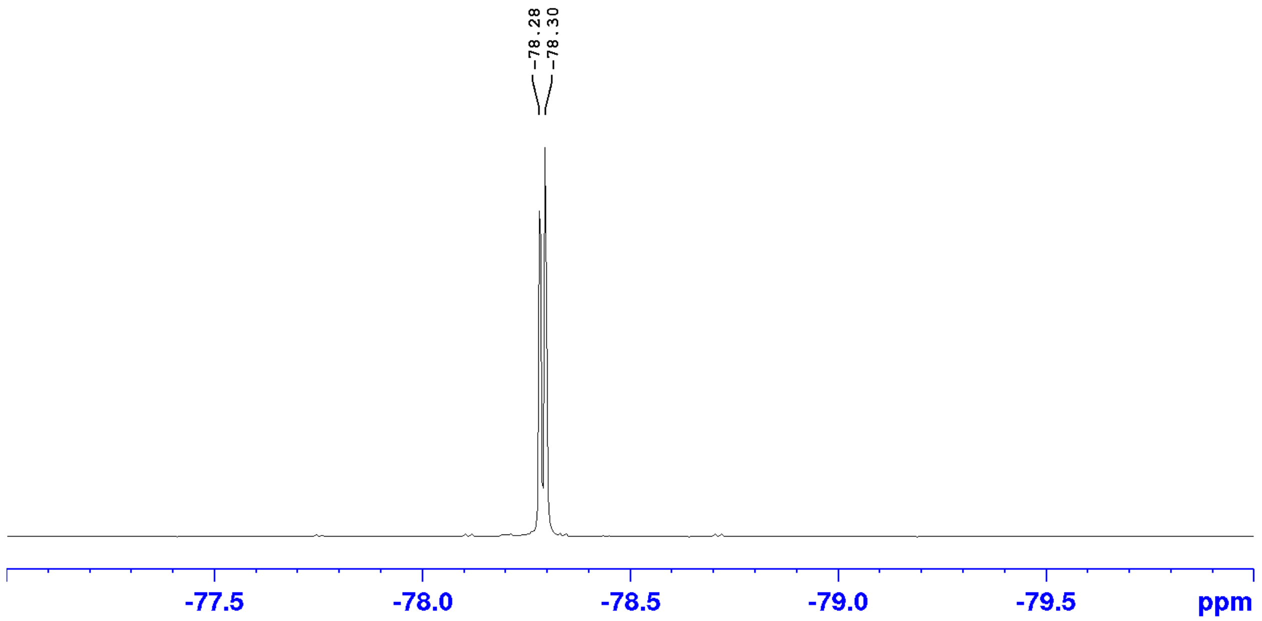

Supplementary Figure 35. ^{19}F NMR of compound 1i. The sample has been recorded in 470 MHz, $CDCl_3$ at 25 °C.

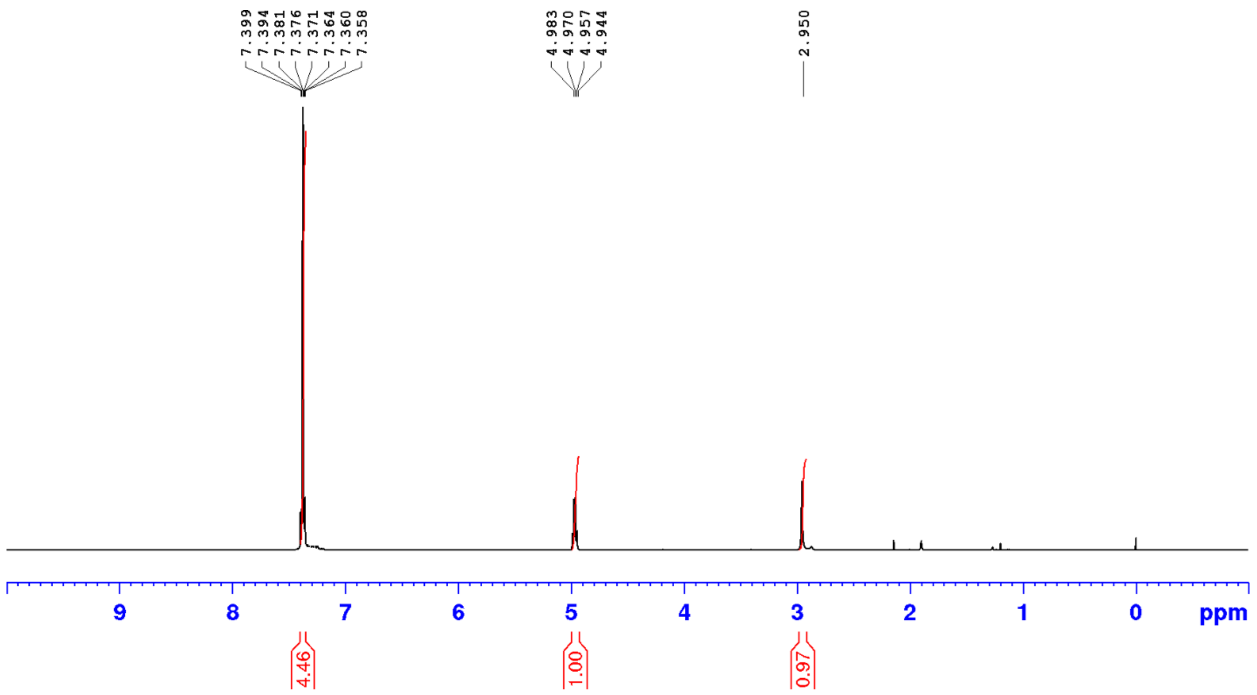

Supplementary Figure 36. ^{1}H NMR of compound 1j. The sample has been recorded in 500 MHz, $CDCl_3$ at 25 °C.

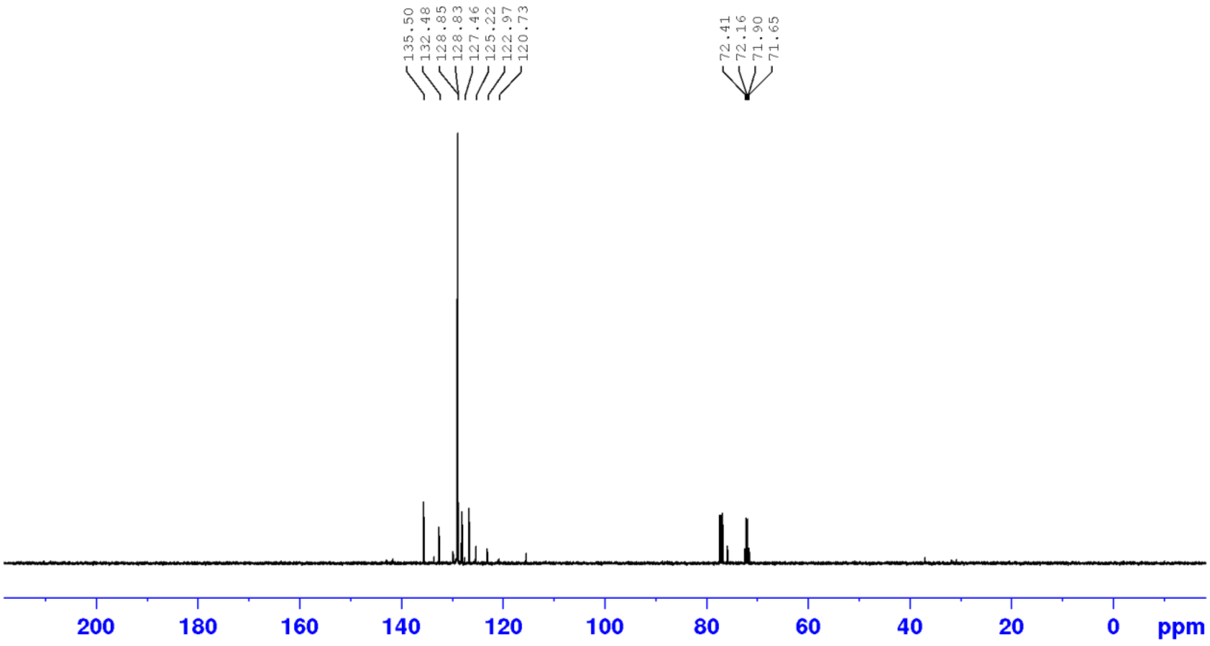

Supplementary Figure 37. ^{13}C NMR of compound 1j. The sample has been recorded in 125 MHz, $CDCl_3$ at 25 °C.

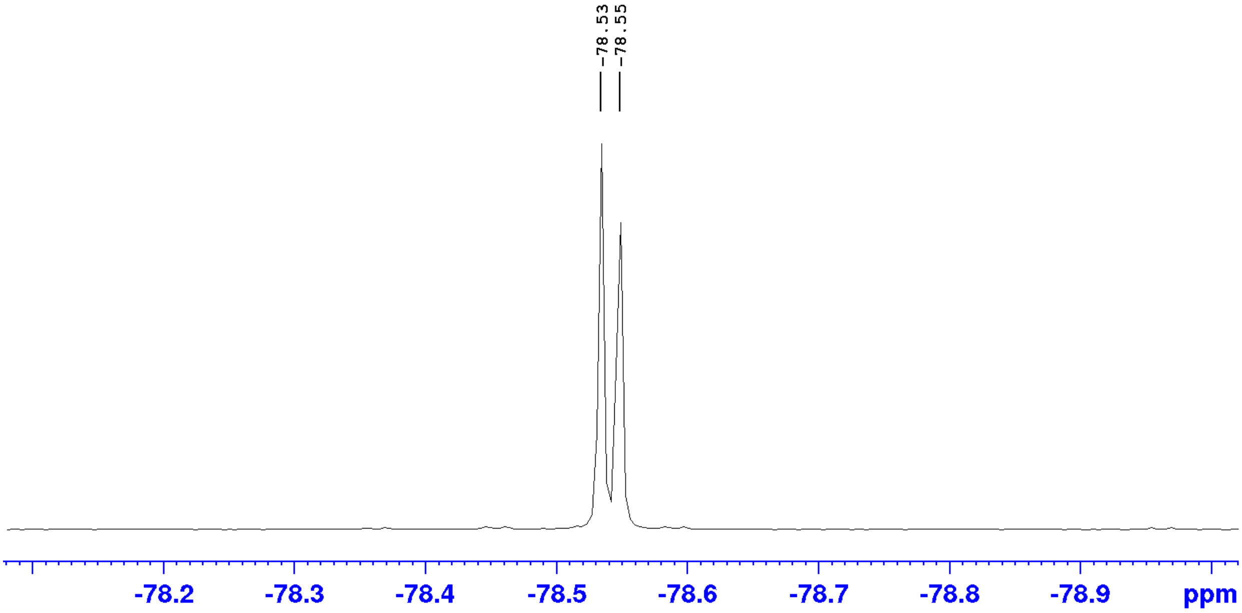

Supplementary Figure 38. ^{19}F NMR of compound 1j. The sample has been recorded in 470 MHz, $CDCl_3$ at 25 °C.

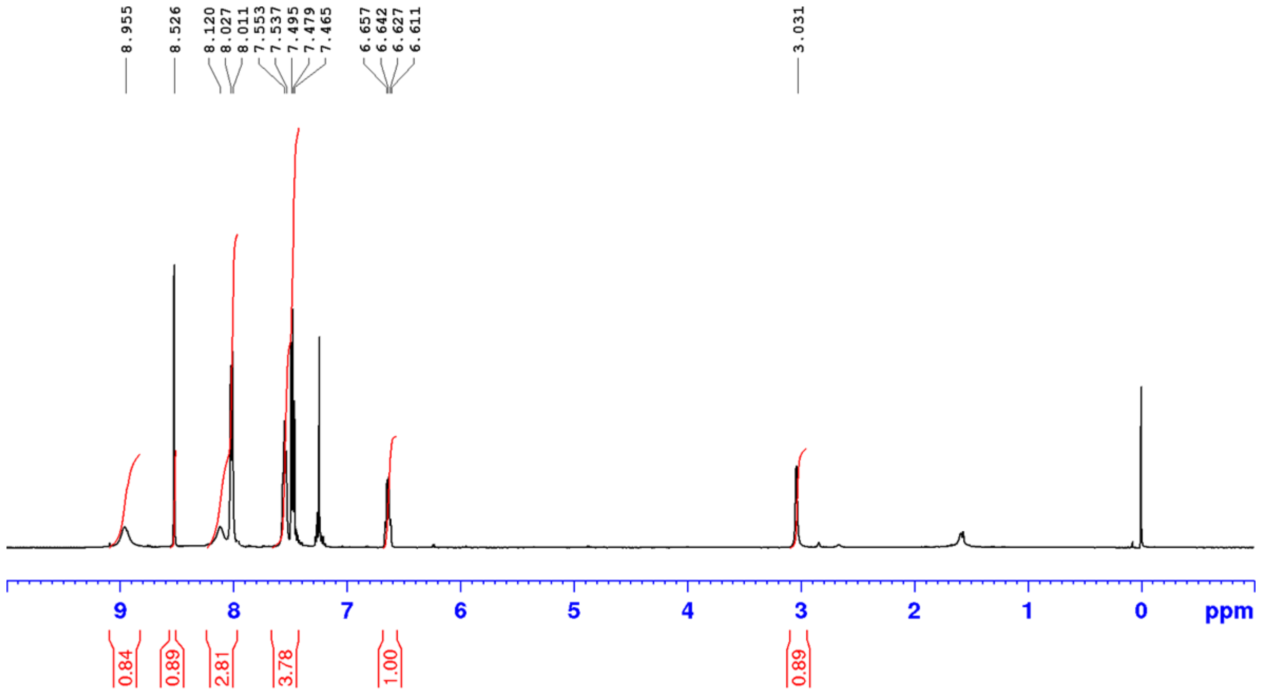

Supplementary Figure 39. ^{1}H NMR of compound 1k. The sample has been recorded in 500 MHz, $CDCl_3$ at 25 °C.

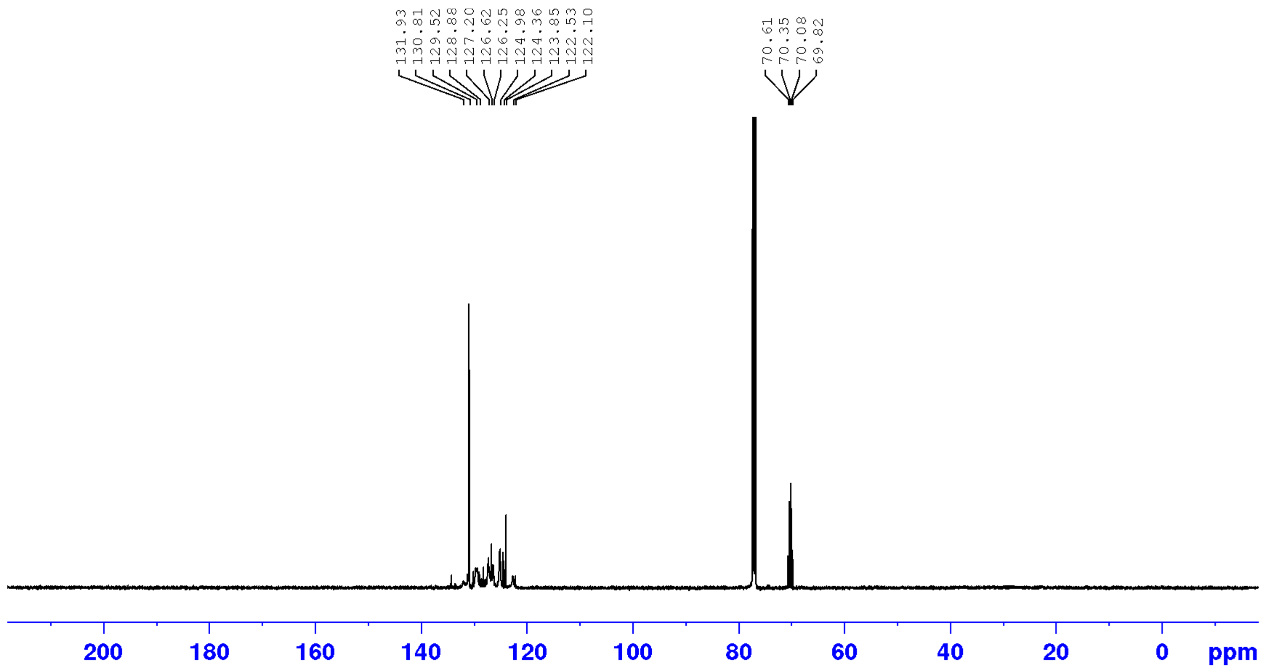

Supplementary Figure 40. ^{13}C NMR of compound 1k. The sample has been recorded in 125 MHz, $CDCl_3$ at 25 °C.

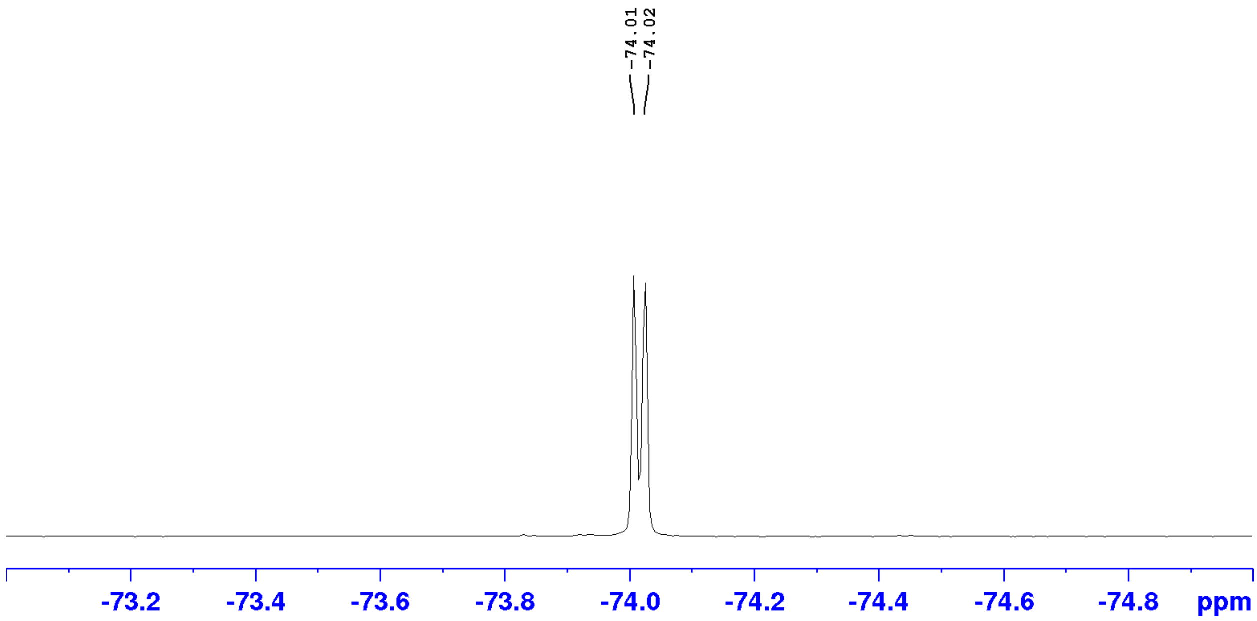

Supplementary Figure 41. ^{19}F NMR of compound 1k. The sample has been recorded in 470 MHz, $CDCl_3$ at 25 °C.

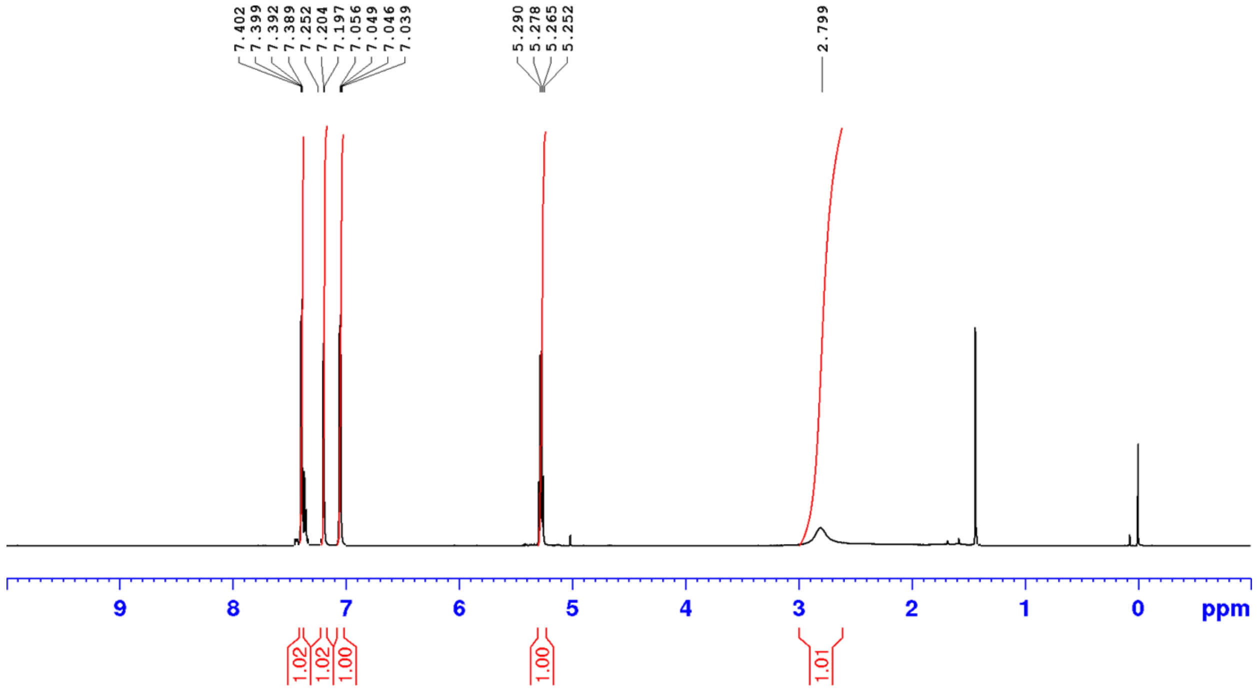

Supplementary Figure 42. ^{1}H NMR of compound 1l. The sample has been recorded in 500 MHz, $CDCl_3$ at 25 °C.

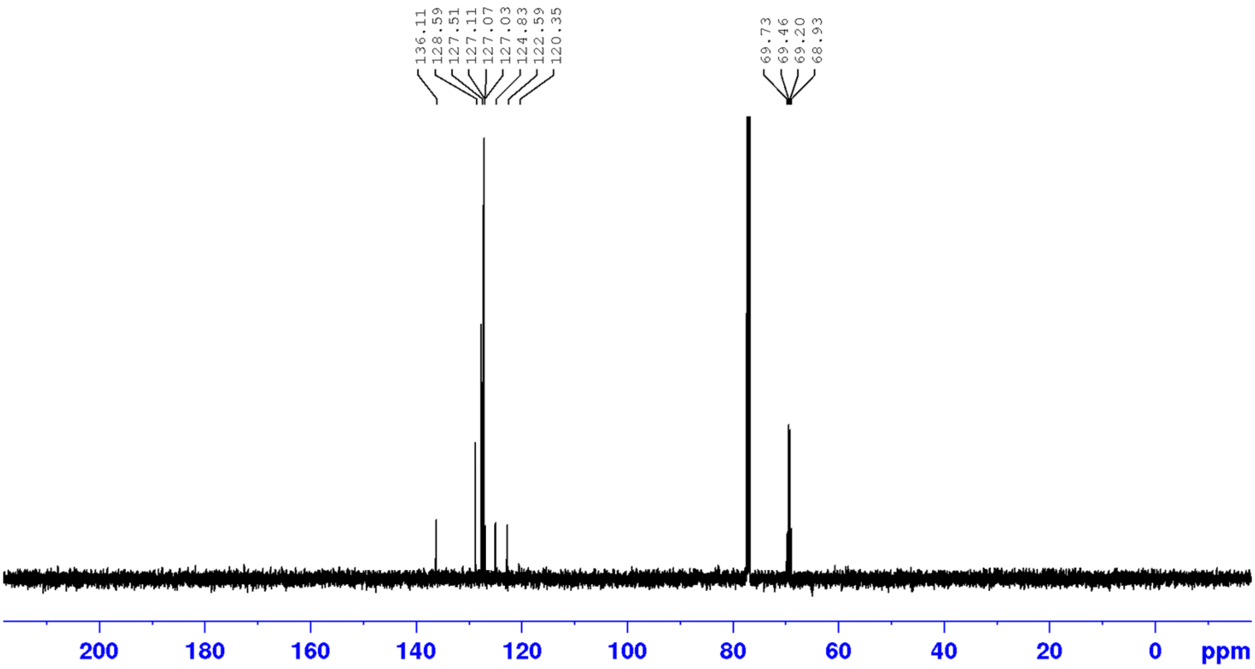

Supplementary Figure 43. ^{13}C NMR of compound 1l. The sample has been recorded in 125 MHz, $CDCl_3$ at 25 °C.

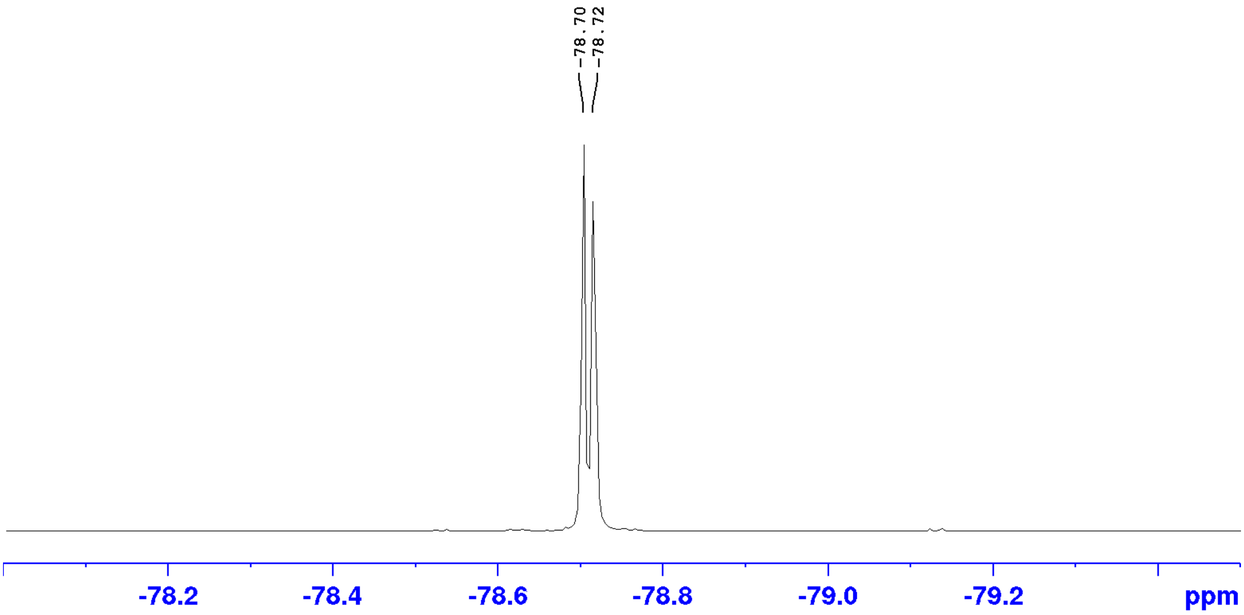

Supplementary Figure 44. ^{19}F NMR of compound 1l. The sample has been recorded in 470 MHz, $CDCl_3$ at 25 °C.

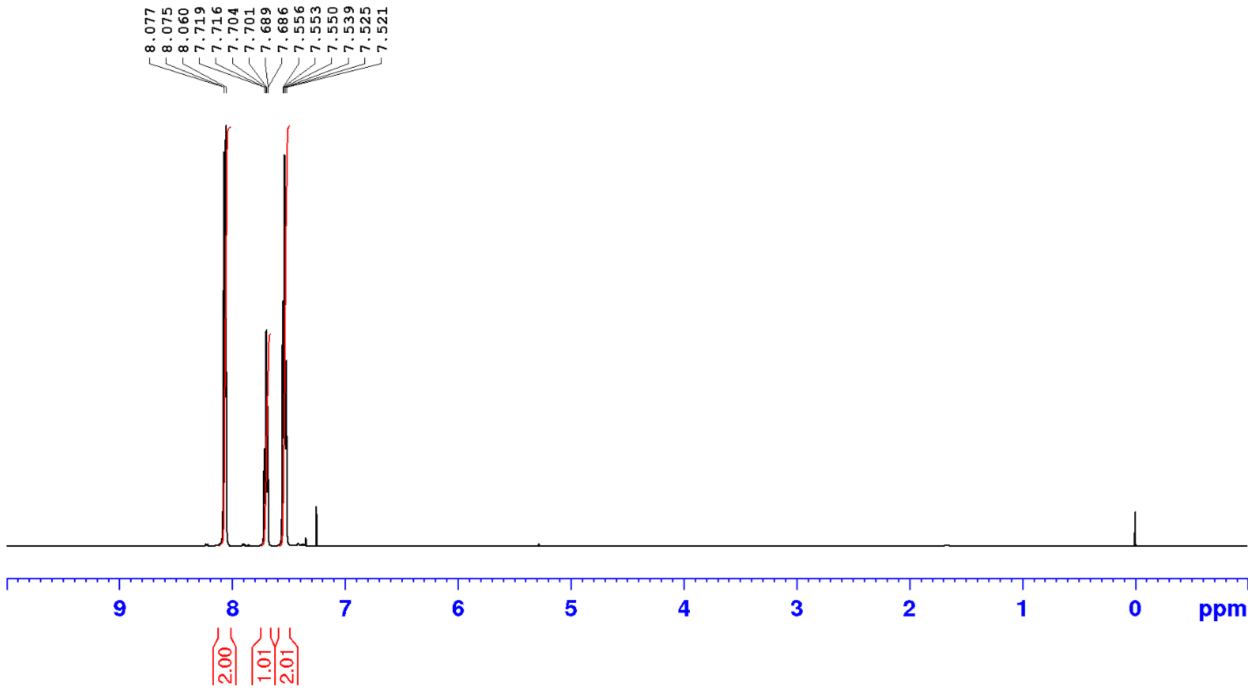

Supplementary Figure 45. ^{1}H NMR of compound 1m. The sample has been recorded in 500 MHz, $CDCl_3$ at 25 °C.

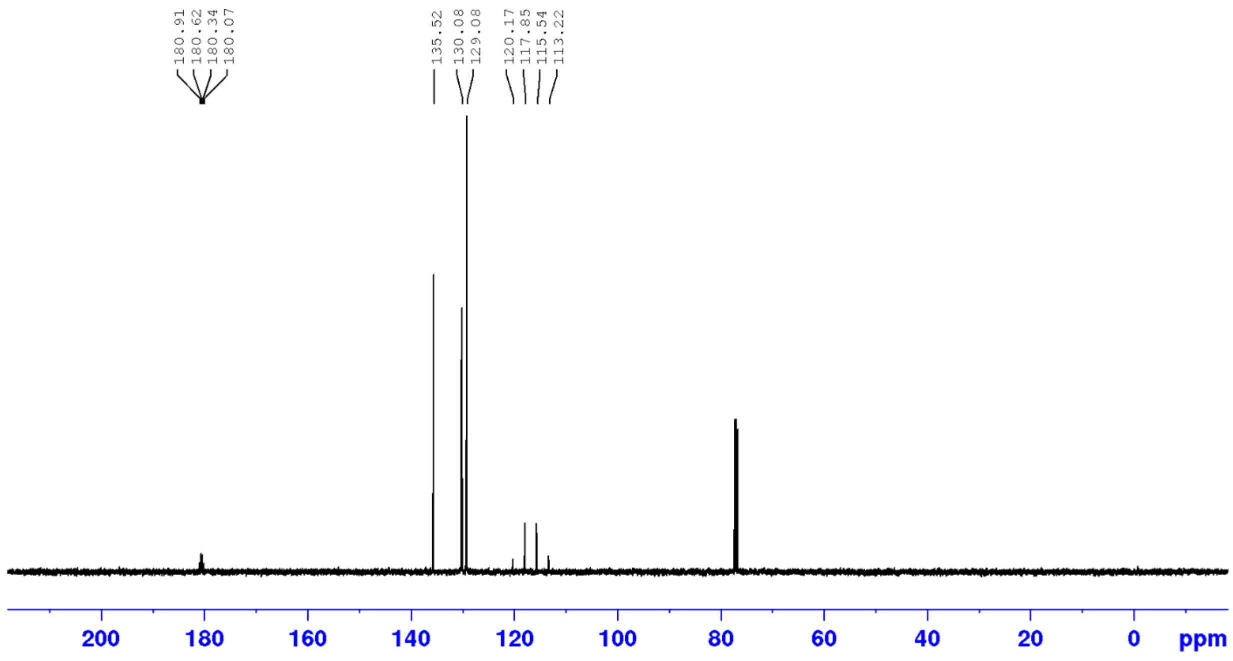

Supplementary Figure 46. ^{13}C NMR of compound 1m. The sample has been recorded in 125 MHz, $CDCl_3$ at 25 °C.

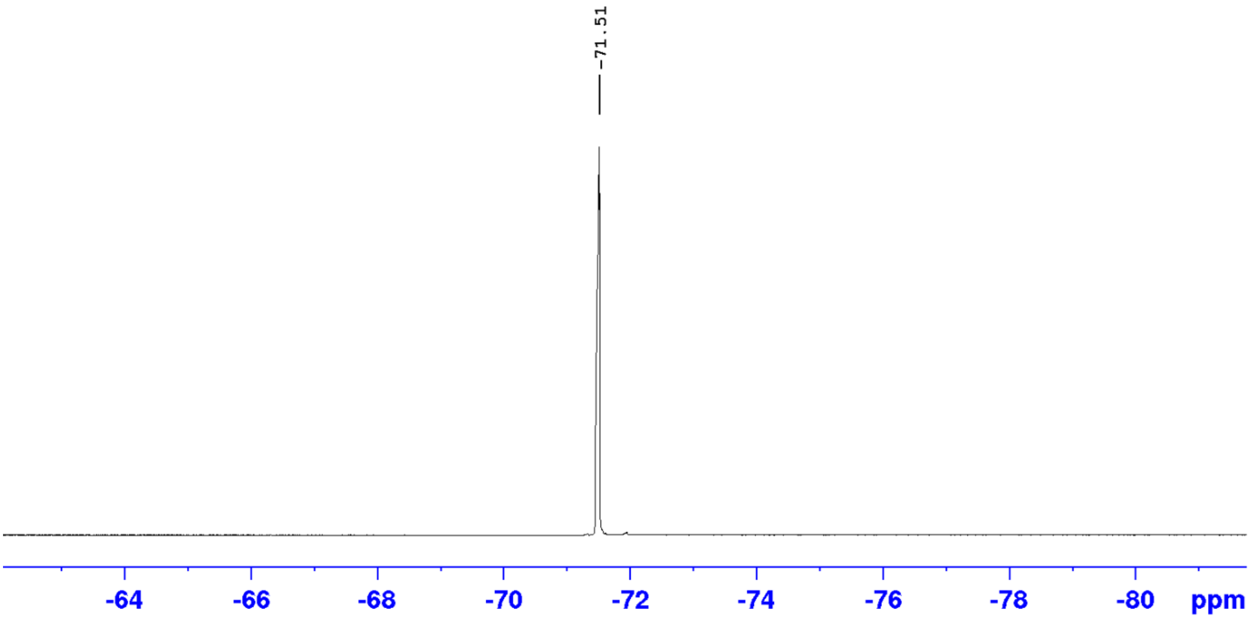

Supplementary Figure 47. ^{19}F NMR of compound 1m. The sample has been recorded in 470 MHz, $CDCl_3$ at 25 °C.

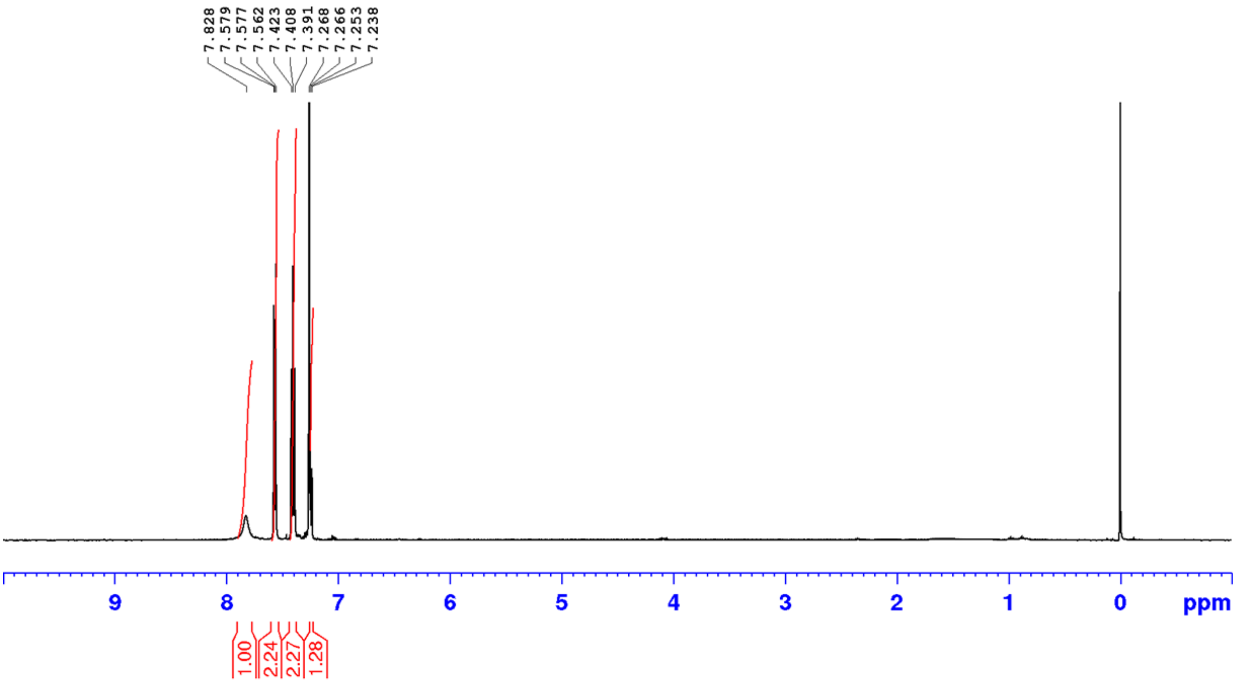

Supplementary Figure 48. ^{1}H NMR of compound 1n. The sample has been recorded in 500 MHz, $CDCl_3$ at 25 °C.

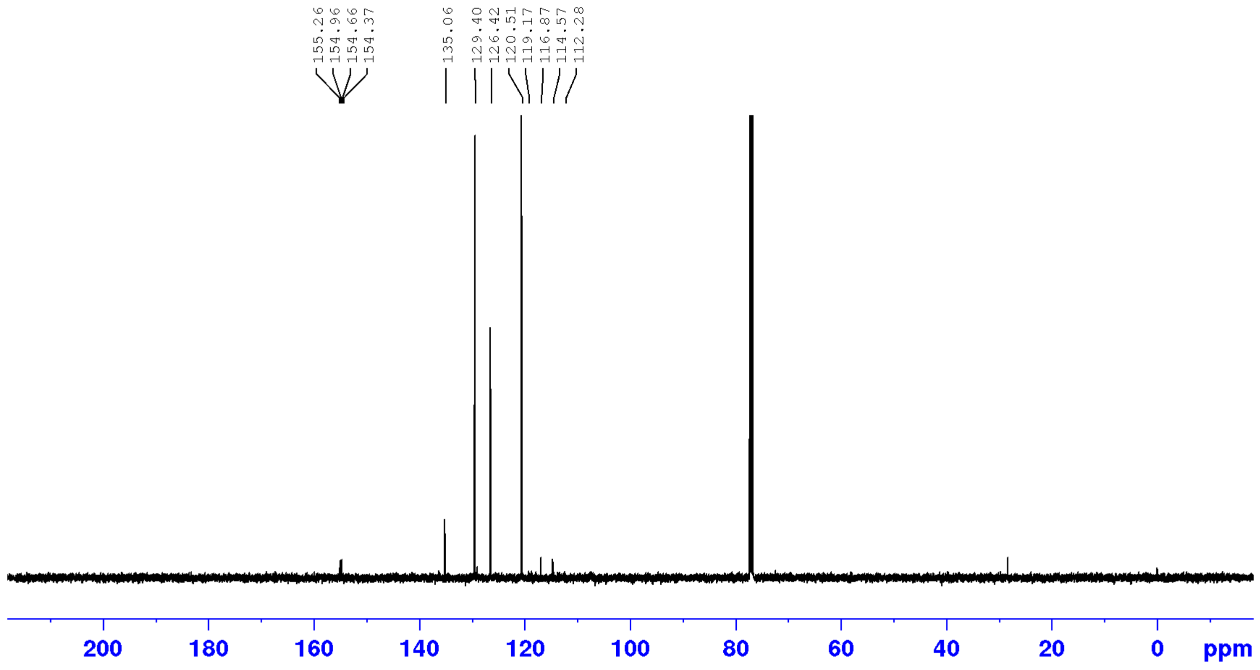

Supplementary Figure 49. ^{13}C NMR of compound 1n. The sample has been recorded in 125 MHz, $CDCl_3$ at 25 °C.

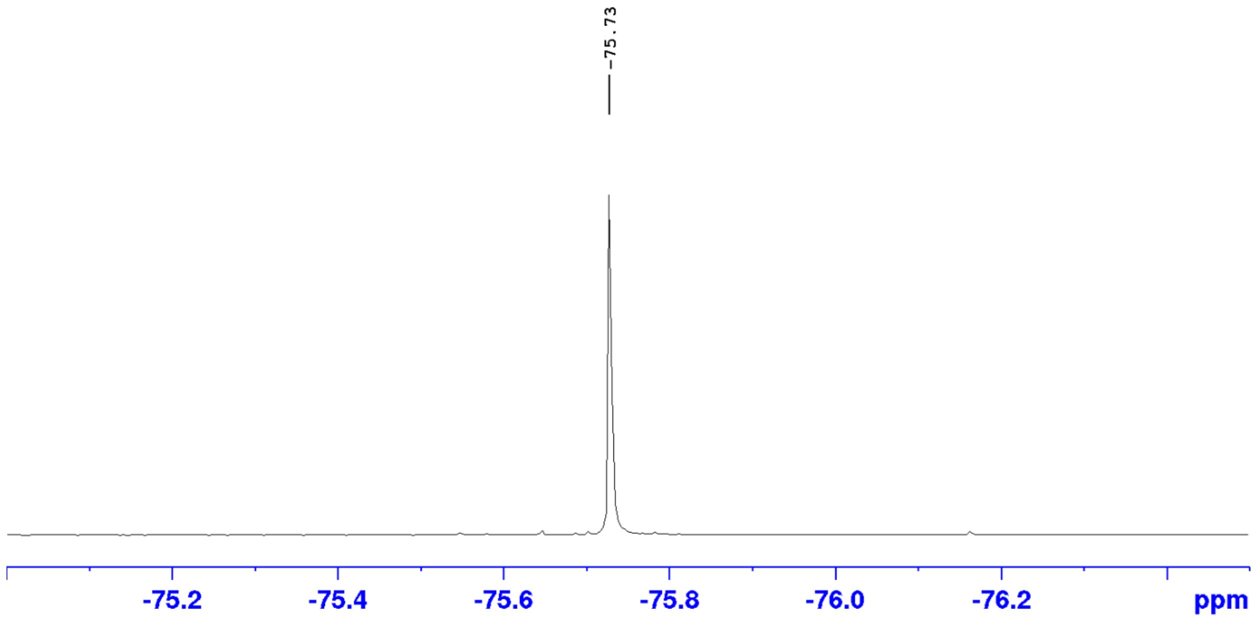

Supplementary Figure 50. ^{19}F NMR of compound 1n. The sample has been recorded in 470 MHz, $CDCl_3$ at 25 °C.

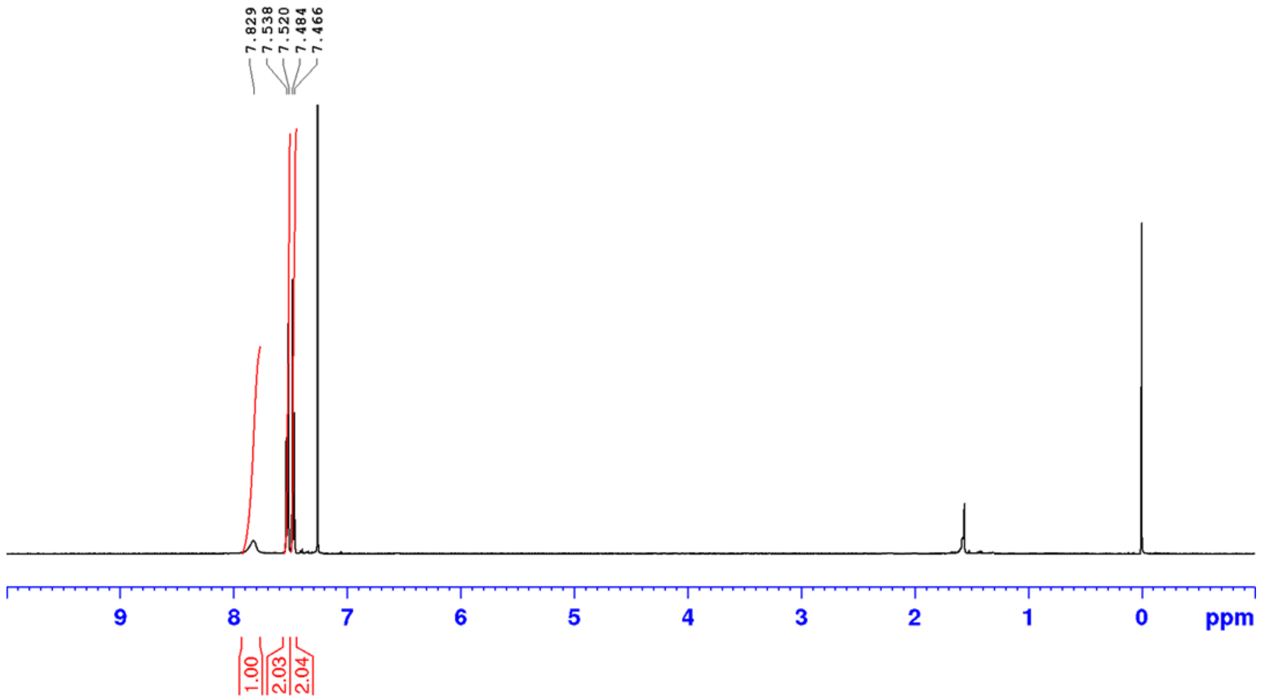

Supplementary Figure 51. ^{1}H NMR of compound 1o. The sample has been recorded in 500 MHz, $CDCl_3$ at 25 °C.

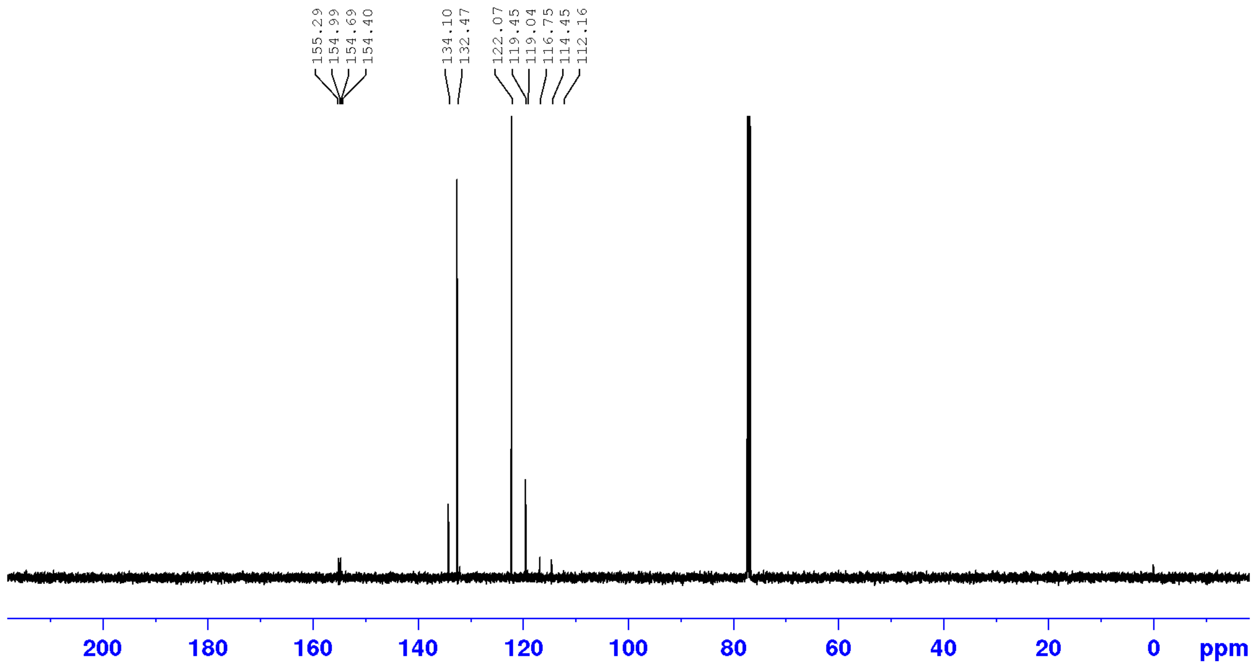

Supplementary Figure 52. ^{13}C NMR of compound 1o. The sample has been recorded in 125 MHz, $CDCl_3$ at 25 °C.

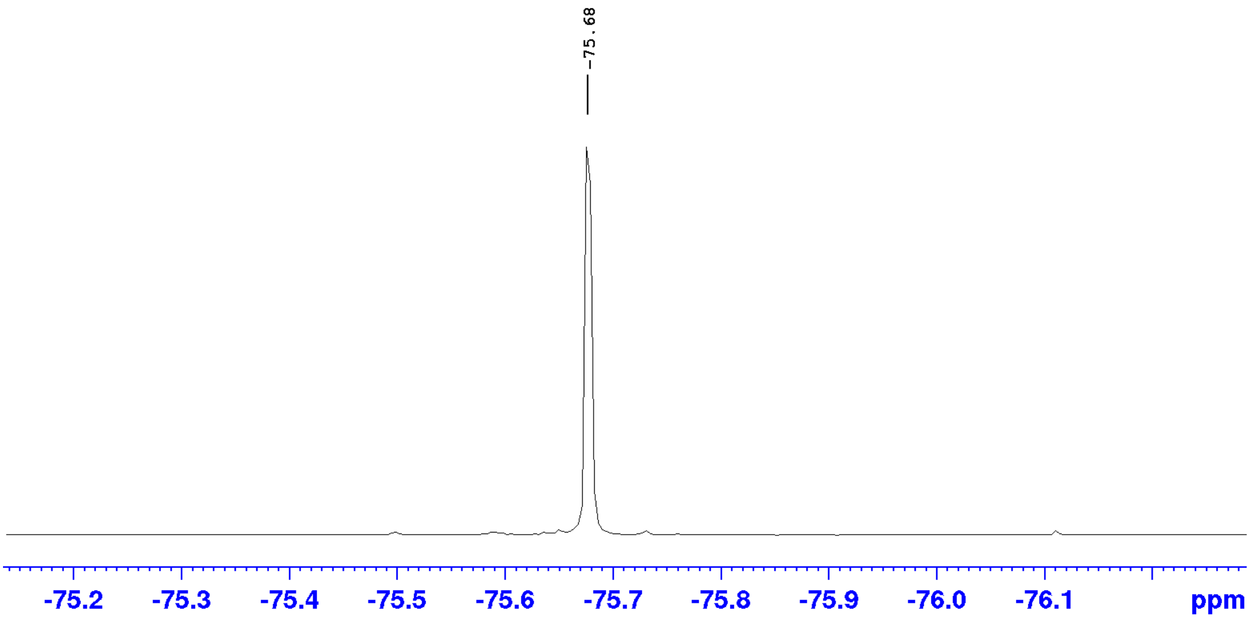

Supplementary Figure 53. ^{19}F NMR of compound 1o. The sample has been recorded in 470 MHz, $CDCl_3$ at 25 °C.

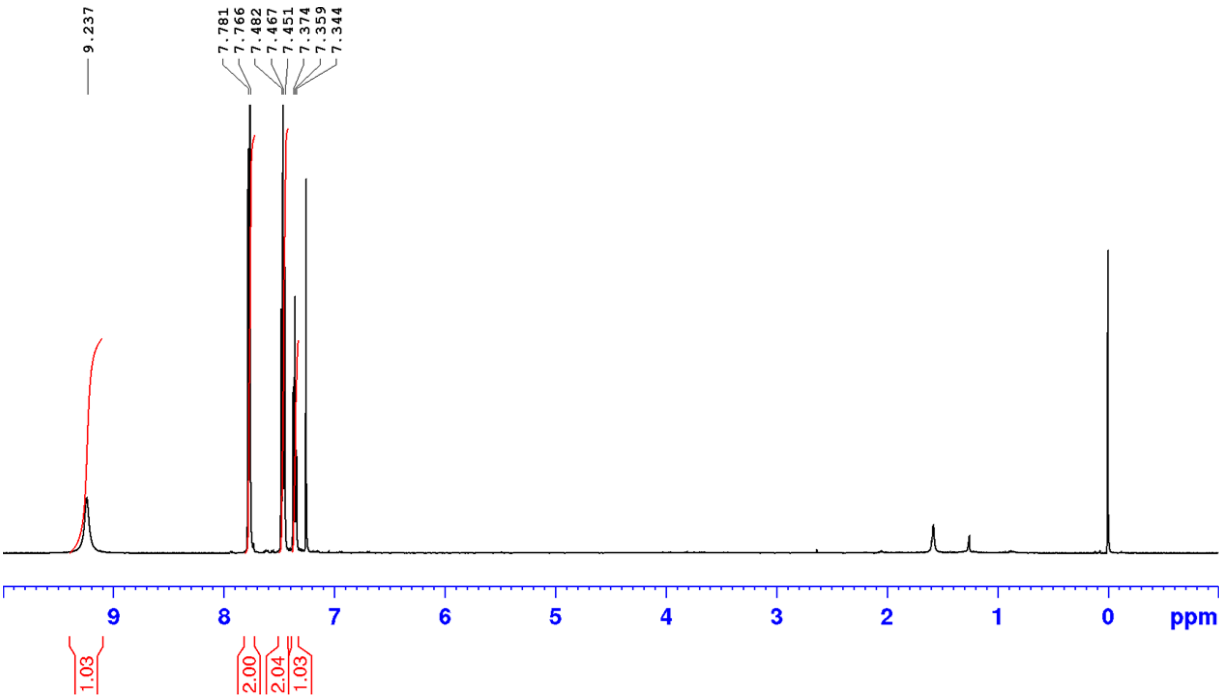

Supplementary Figure 54. ^{1}H NMR of compound 1p. The sample has been recorded in 500 MHz, $CDCl_3$ at 25 °C.

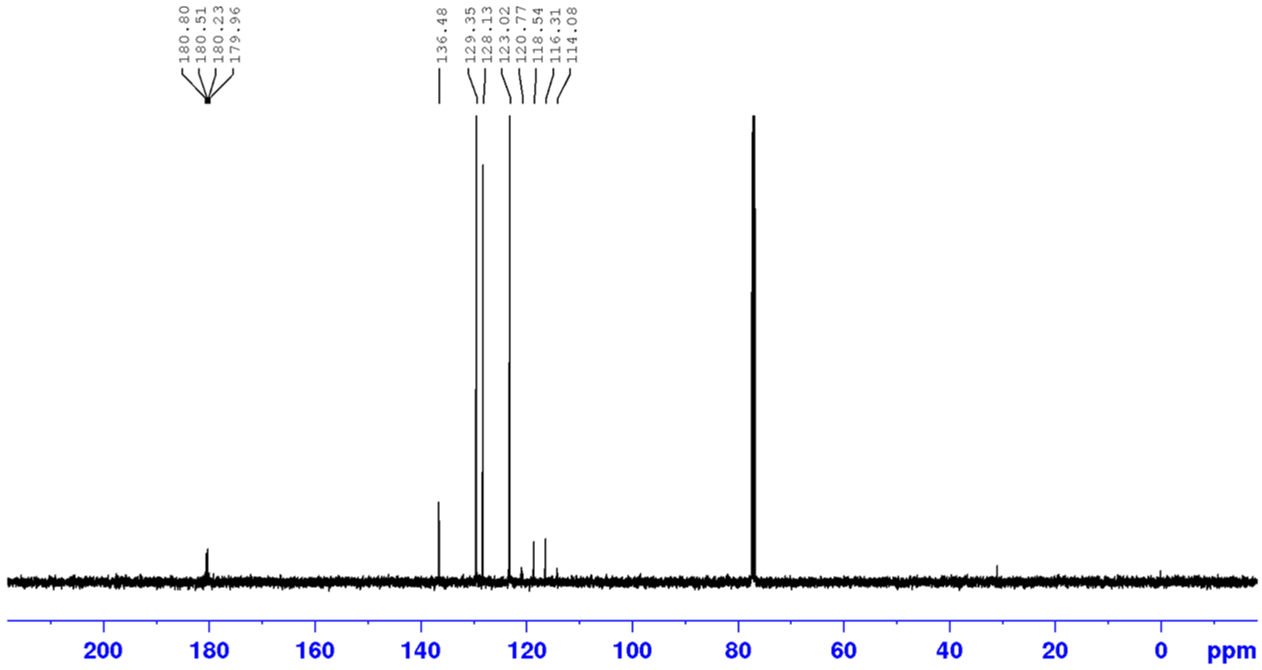

Supplementary Figure 55. ^{13}C NMR of compound 1p. The sample has been recorded in 125 MHz, $CDCl_3$ at 25 °C.

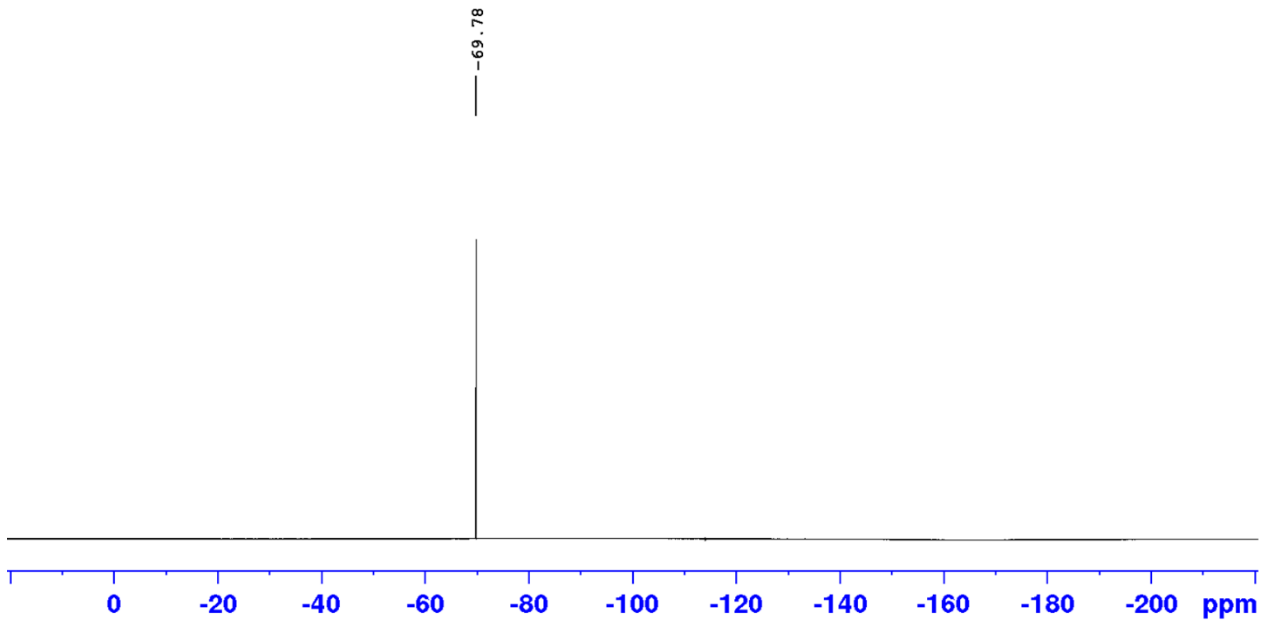

Supplementary Figure 56. ^{19}F NMR of compound 1p. The sample has been recorded in 470 MHz, $CDCl_3$ at 25 °C.

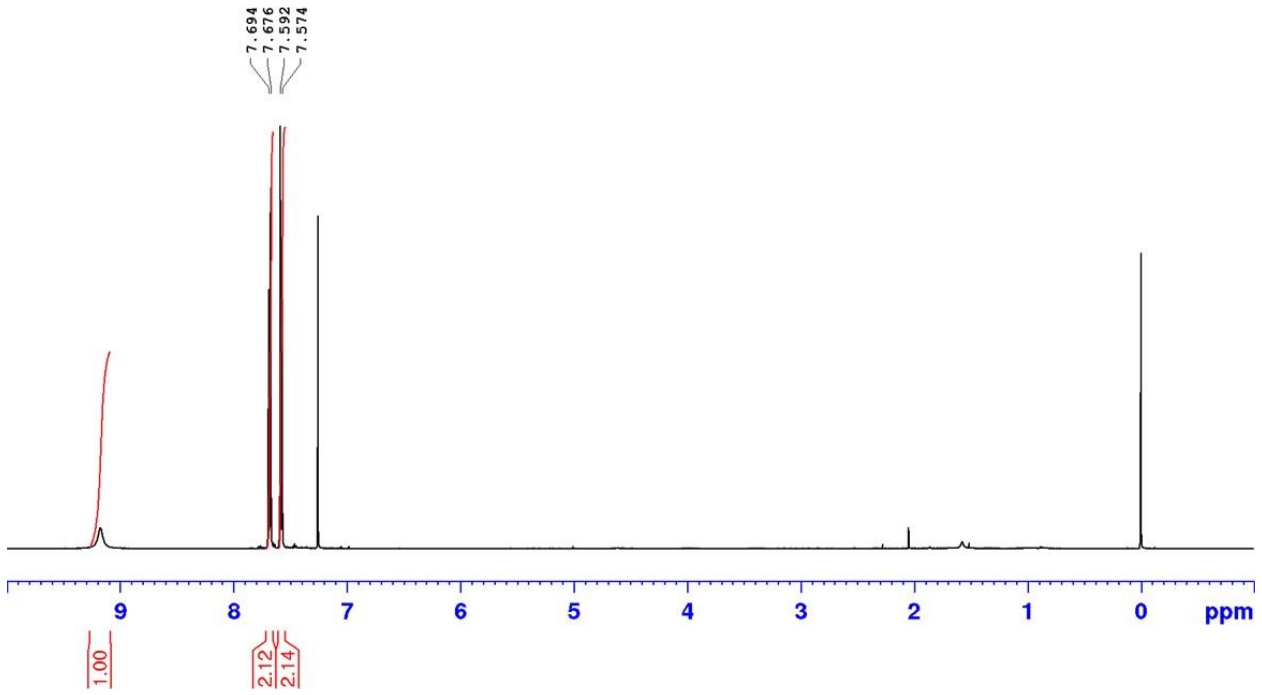

Supplementary Figure 57. ^{1}H NMR of compound 1q. The sample has been recorded in 500 MHz, $CDCl_3$ at 25 °C.

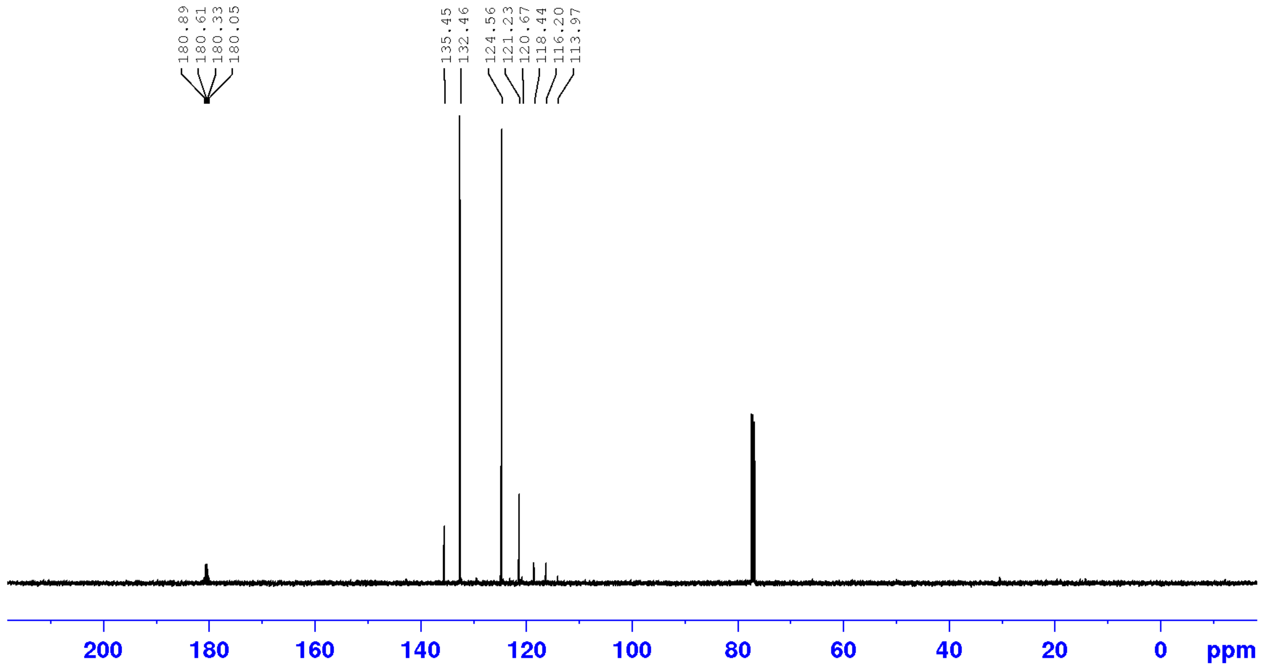

Supplementary Figure 58. ^{13}C NMR of compound 1q. The sample has been recorded in 125 MHz, $CDCl_3$ at 25 °C.

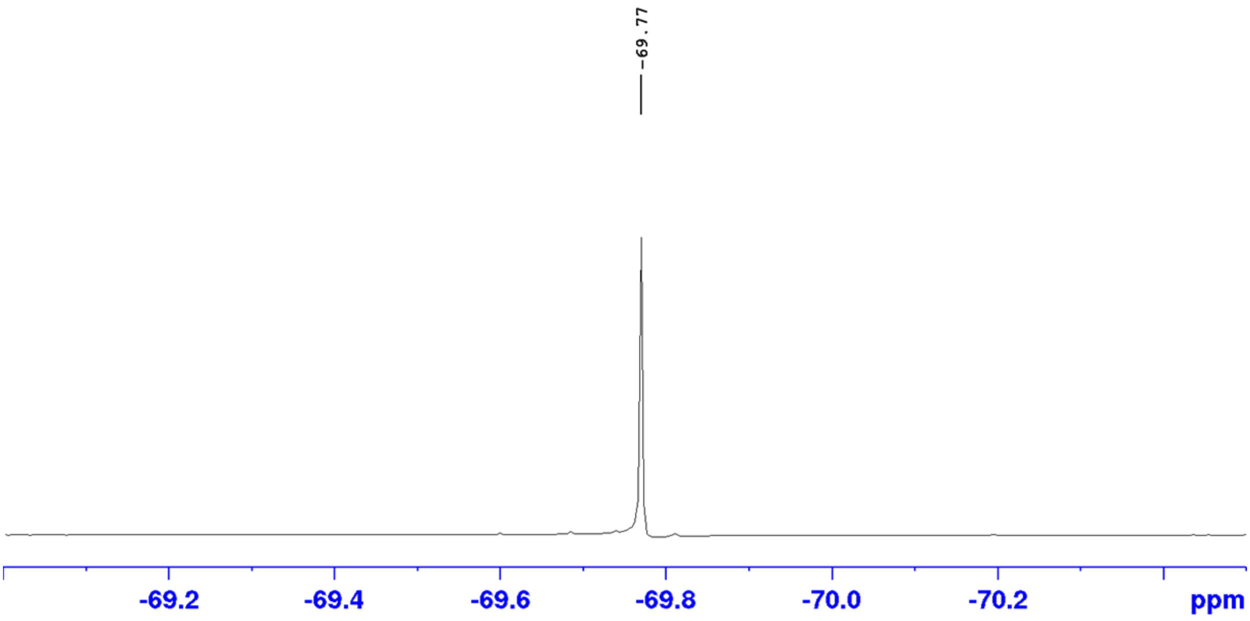

Supplementary Figure 59. ^{19}F NMR of compound 1q. The sample has been recorded in 470 MHz, $CDCl_3$ at 25 °C.

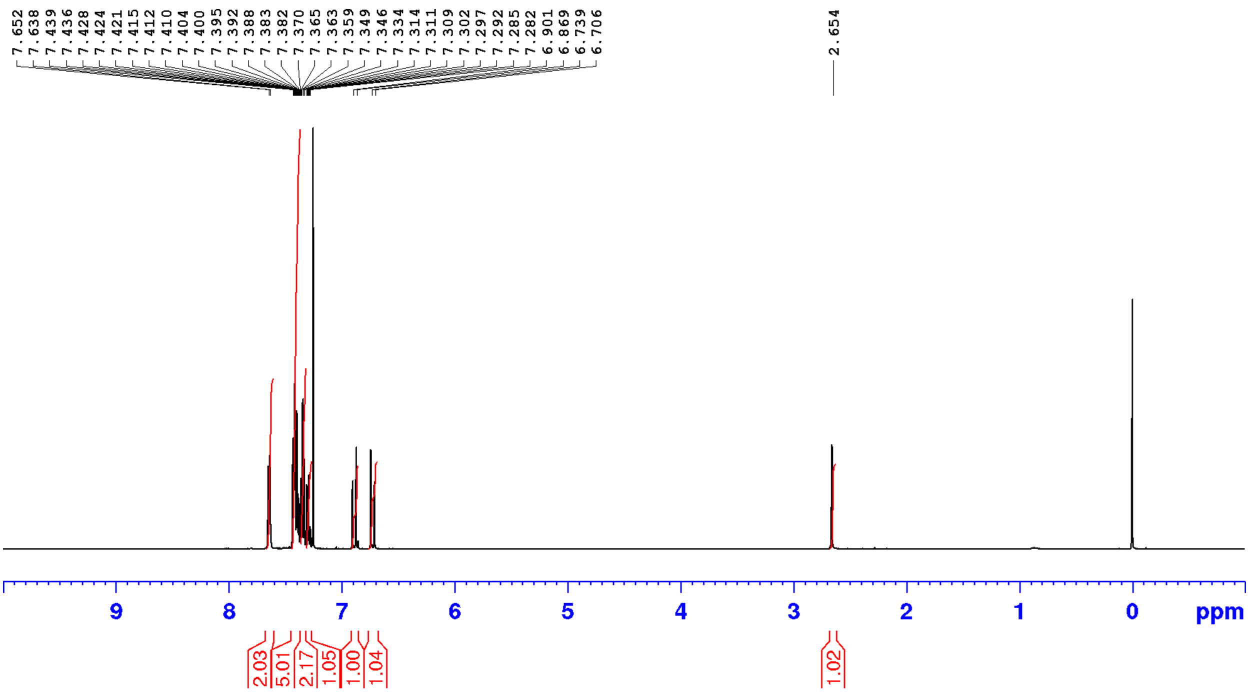

Supplementary Figure 60. ^{1}H NMR of compound 1r. The sample has been recorded in 500 MHz, $CDCl_3$ at 25 °C.

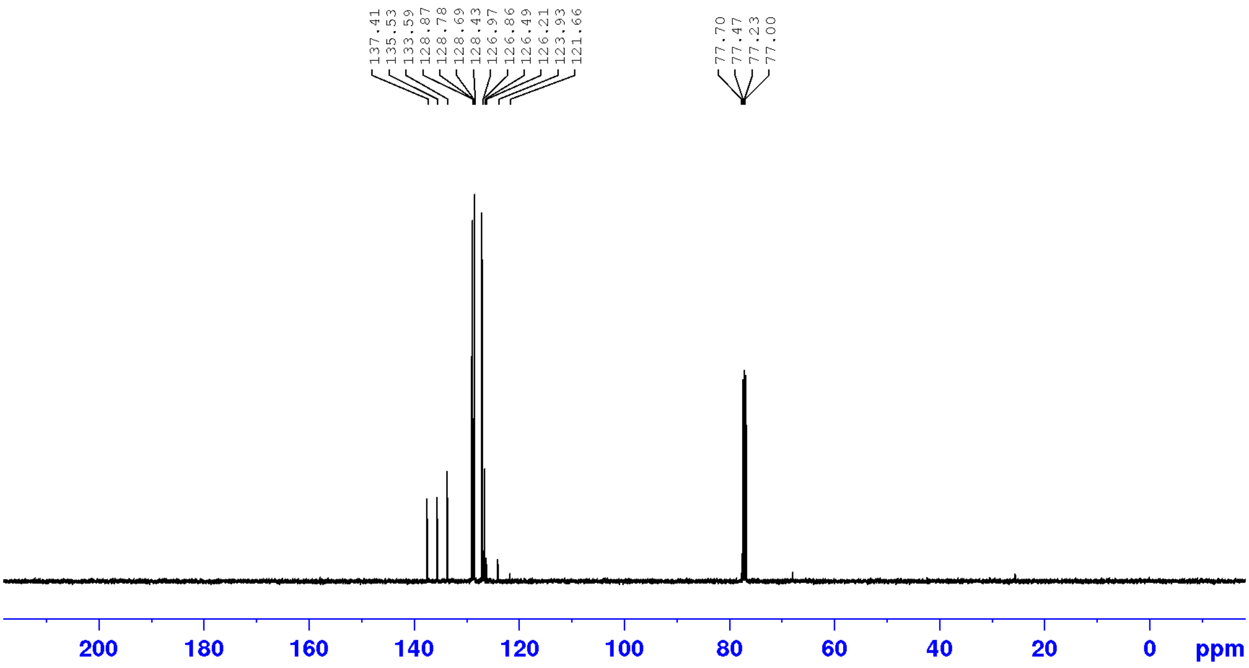

Supplementary Figure 61. ^{13}C NMR of compound 1r. The sample has been recorded in 125 MHz, $CDCl_3$ at 25 °C.

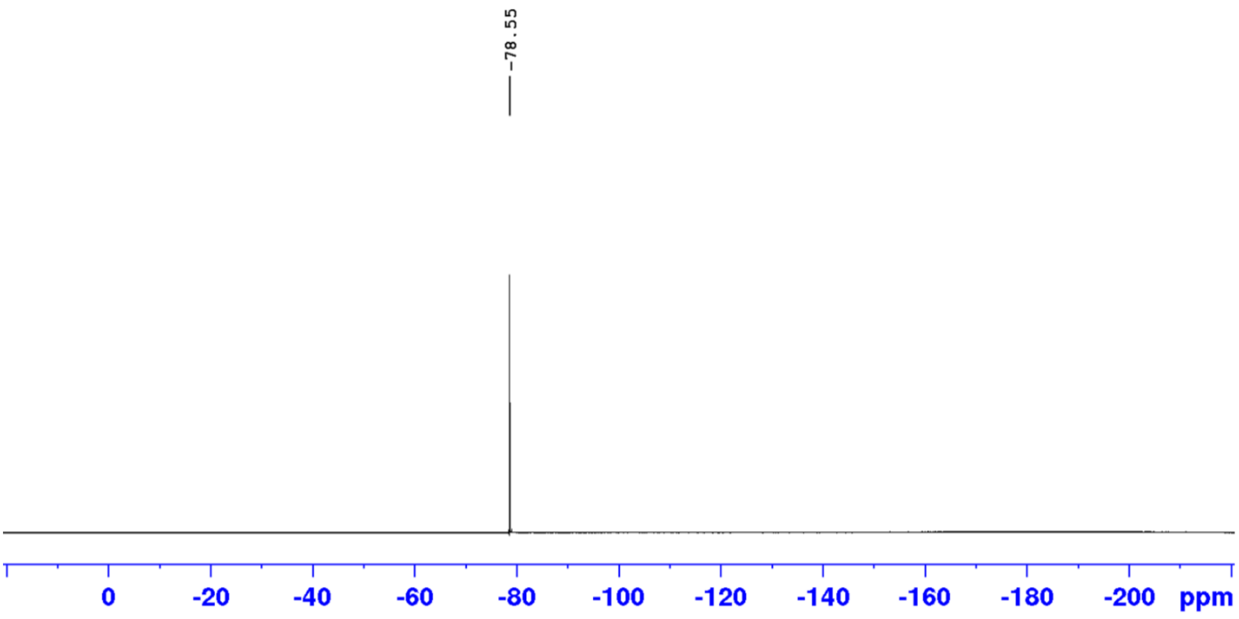

Supplementary Figure 62. ^{19}F NMR of compound 1r. The sample has been recorded in 470 MHz, $CDCl_3$ at 25 °C.

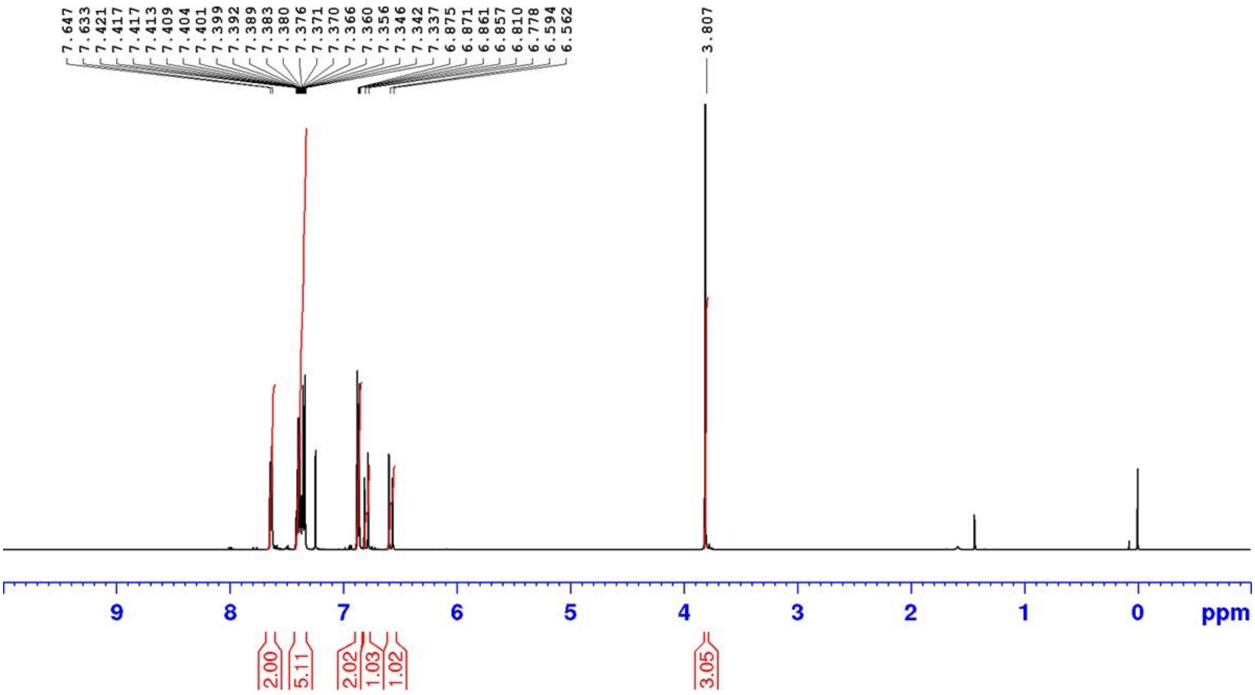

Supplementary Figure 63. ^{1}H NMR of compound 1s. The sample has been recorded in 500 MHz, $CDCl_3$ at 25 ℃.

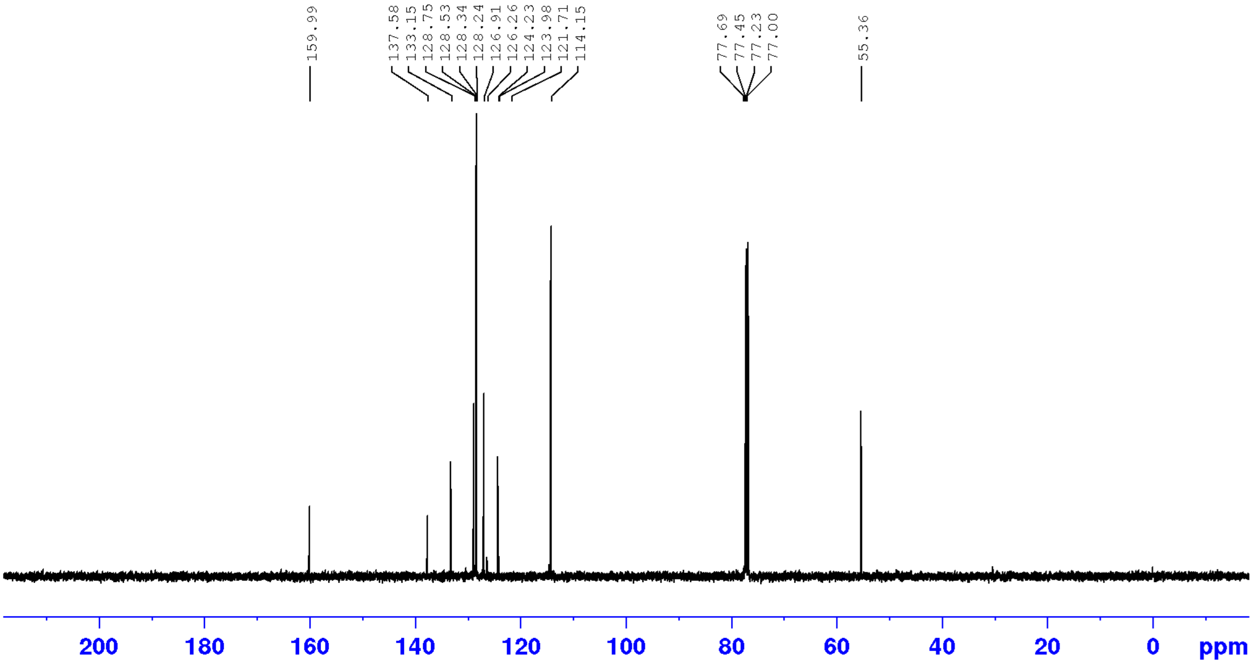

Supplementary Figure 64. ^{13}C NMR of compound 1s. The sample has been recorded in 125 MHz, $CDCl_3$ at 25 ℃.

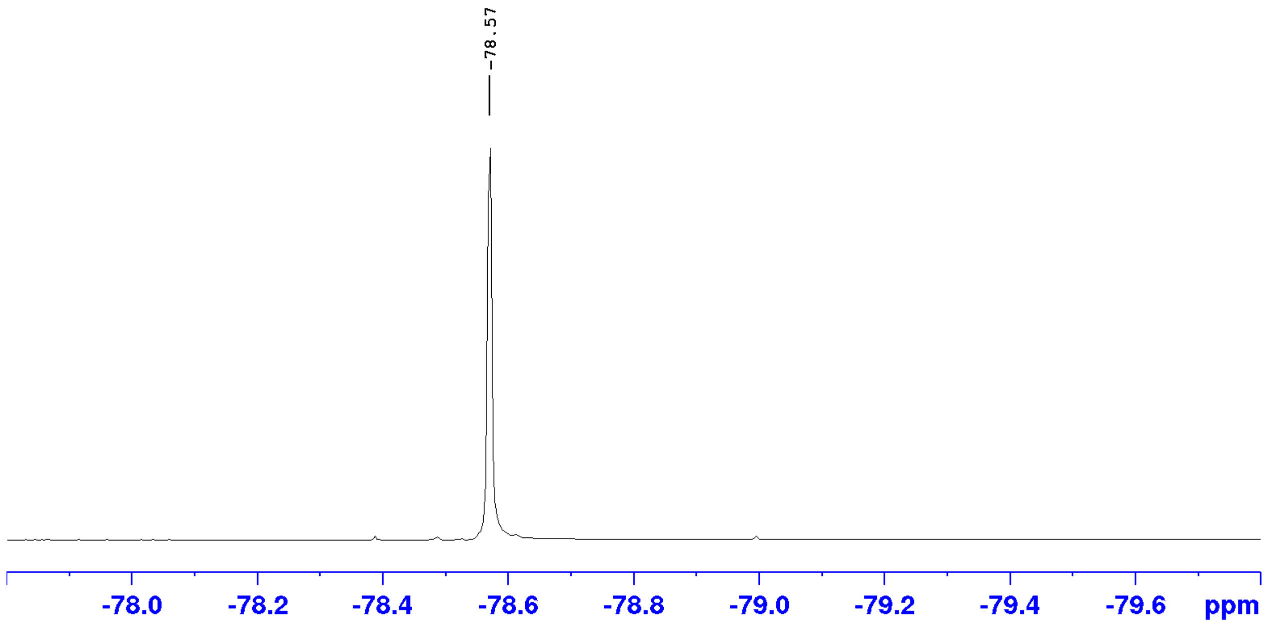

Supplementary Figure 65. ^{19}**F NMR of compound 1s.** The sample has been recorded in 470 MHz, $CDCl_3$ at 25 °C.

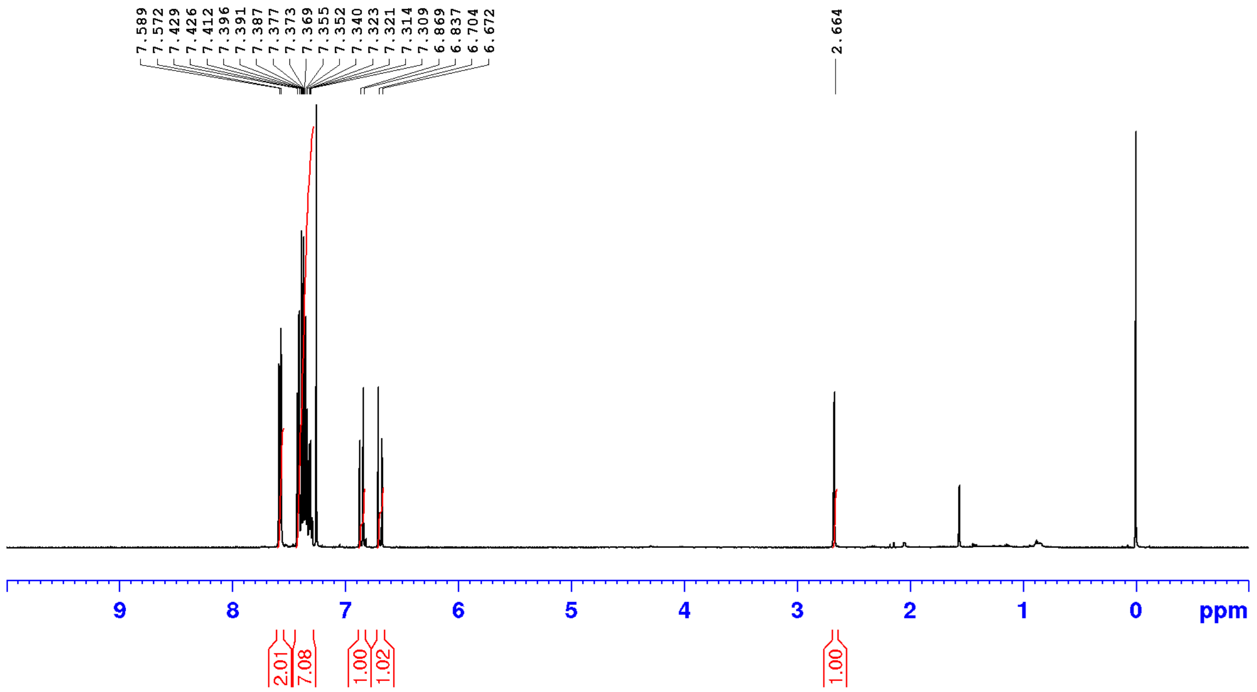

Supplementary Figure 66. ^{1}**H NMR of compound 1t.** The sample has been recorded in 500 MHz, $CDCl_3$ at 25 °C.

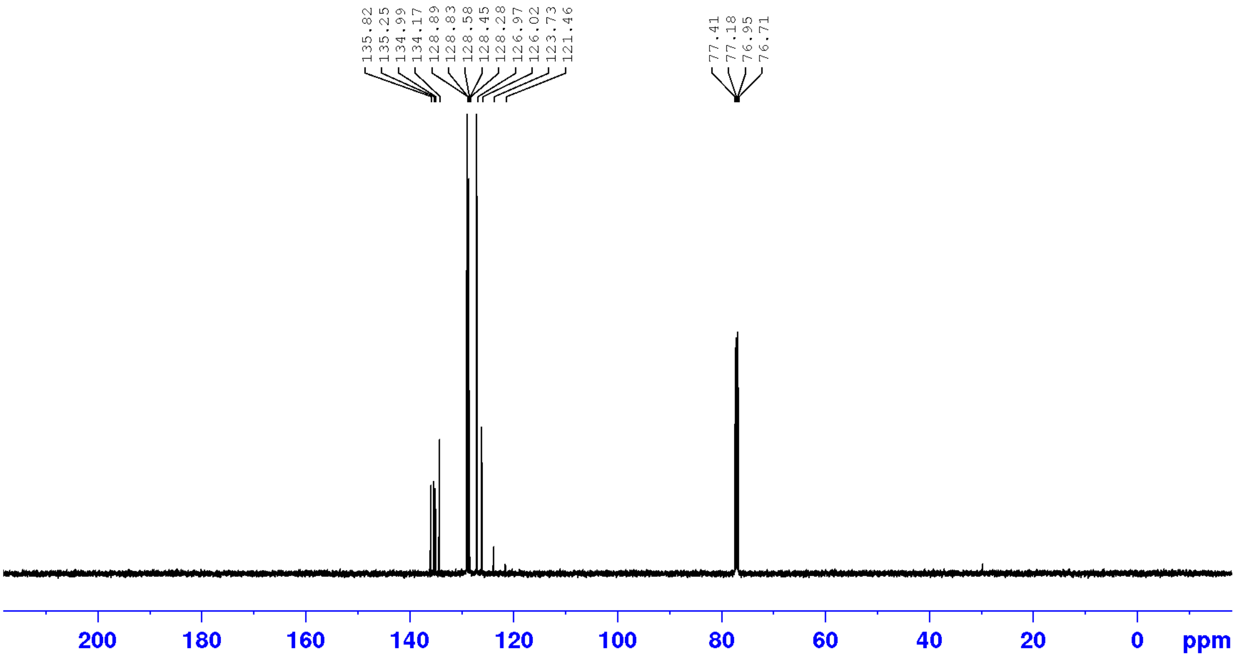

Supplementary Figure 67. ^{13}**C NMR of compound 1t.** The sample has been recorded in 125 MHz, $CDCl_3$ at 25 °C.

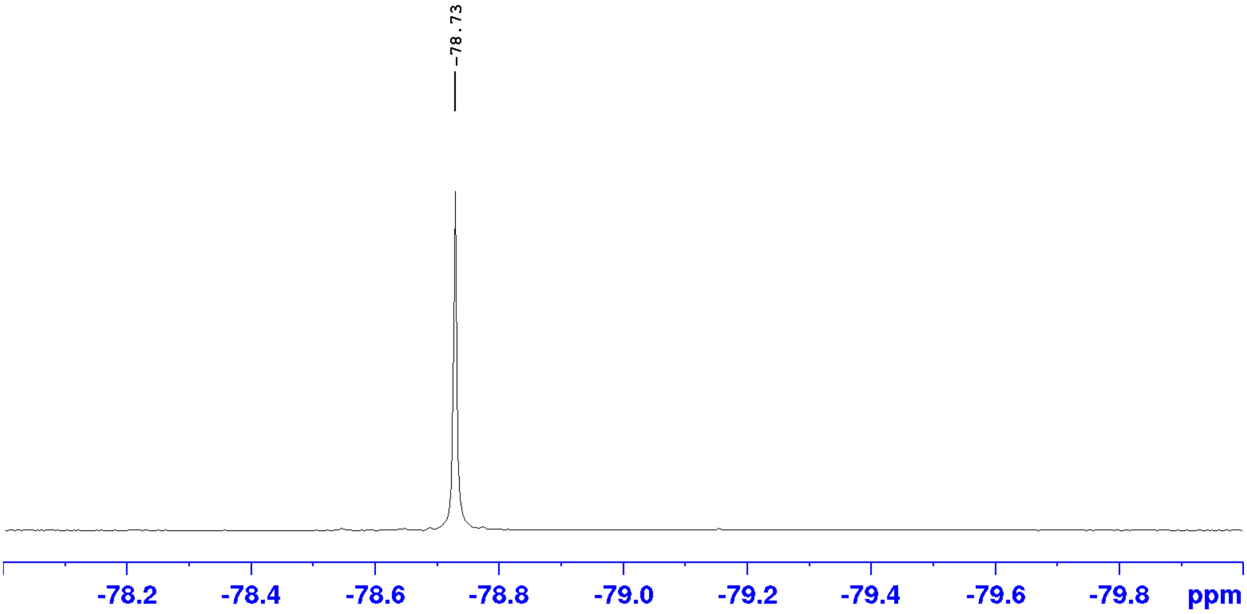

Supplementary Figure 68. ^{19}**F NMR of compound 1t.** The sample has been recorded in 470 MHz, $CDCl_3$ at 25 °C.

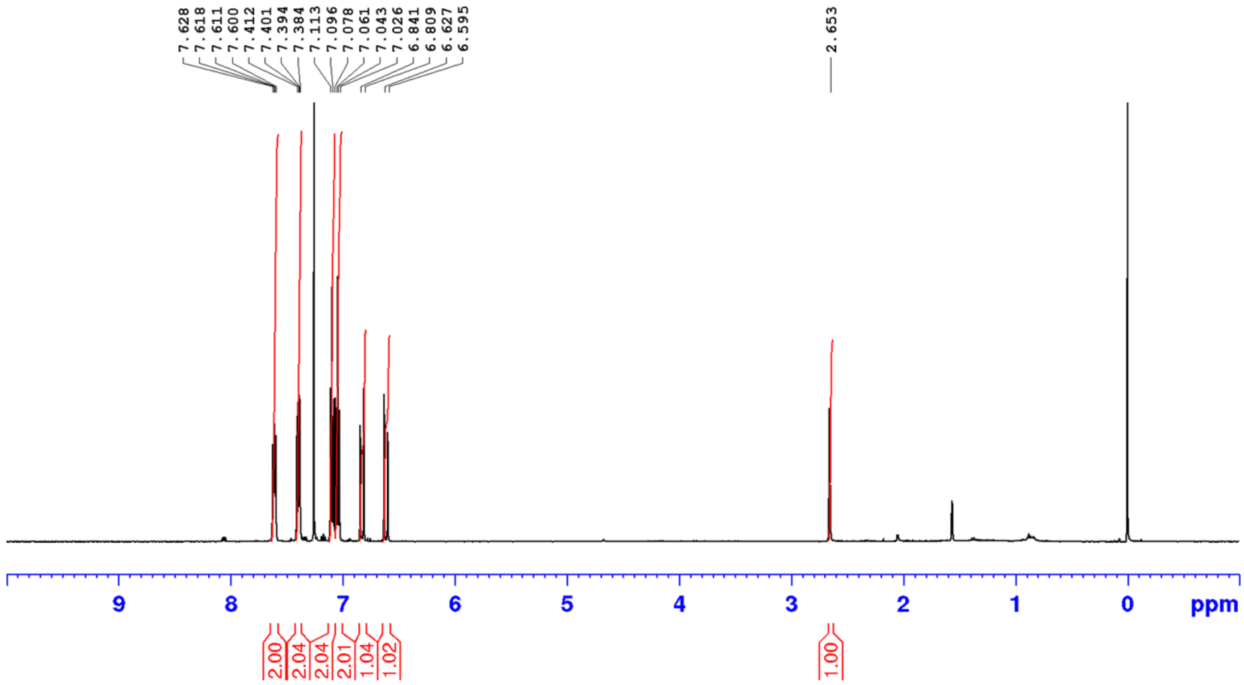

Supplementary Figure 69. ^{1}H NMR of compound 1u. The sample has been recorded in 500 MHz, $CDCl_3$ at 25 °C.

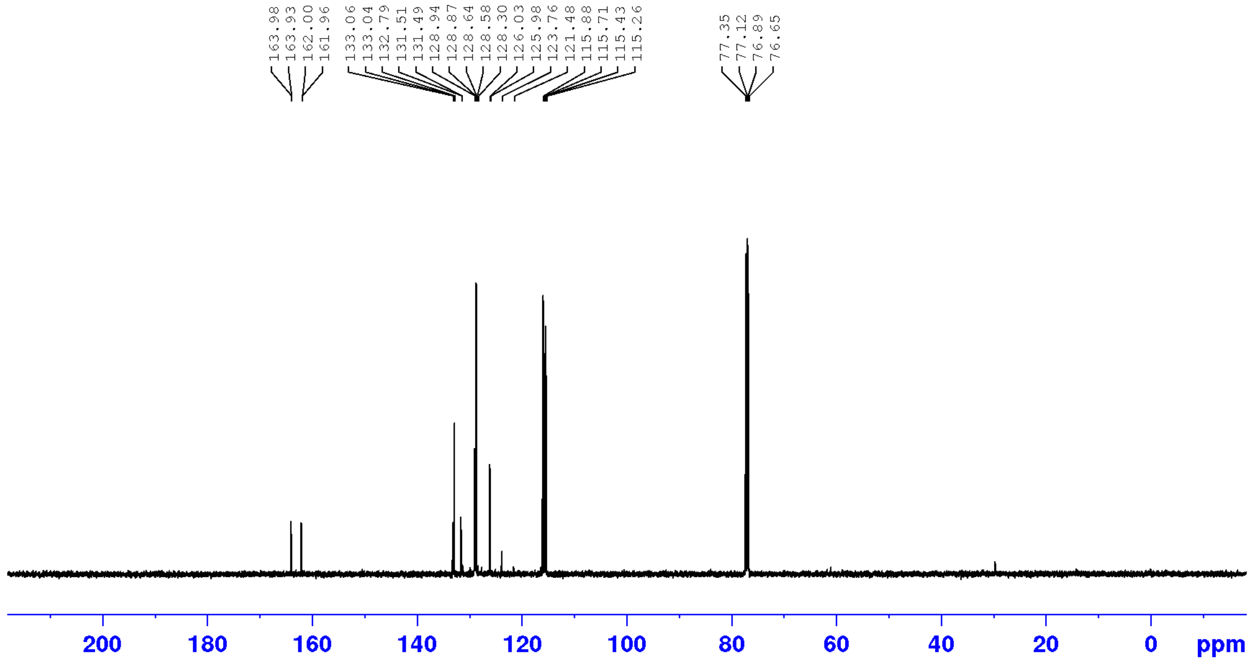

Supplementary Figure 70. ^{13}C NMR of compound 1u. The sample has been recorded in 125 MHz, $CDCl_3$ at 25 °C.

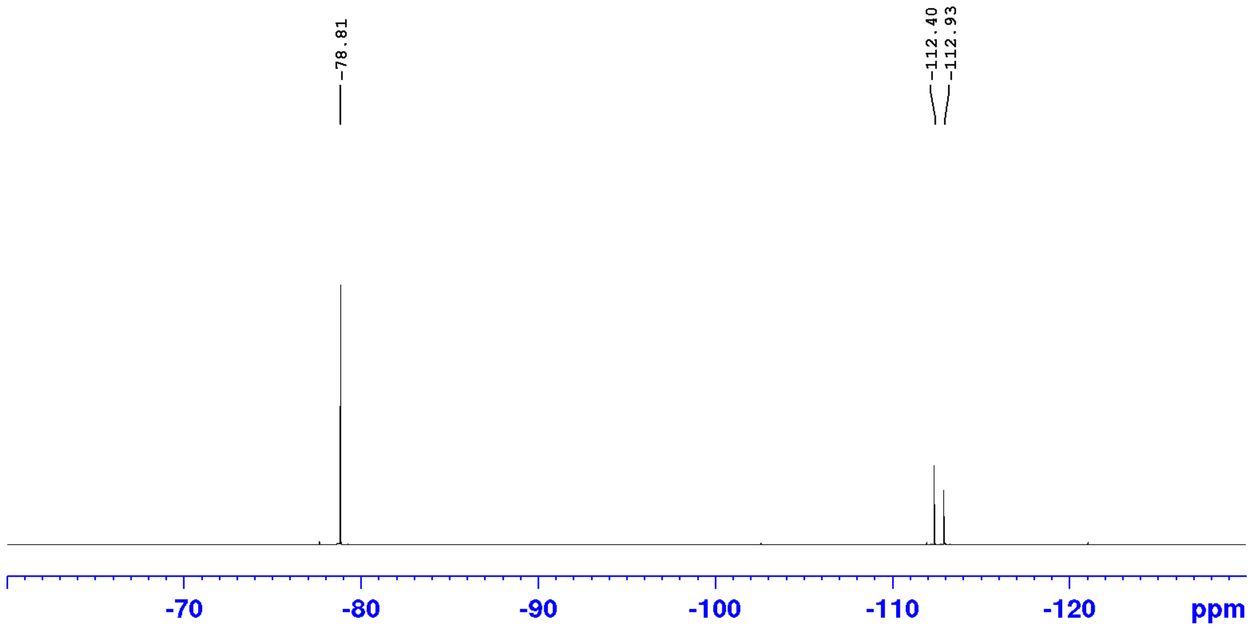

Supplementary Figure 71. ^{19}F NMR of compound 1u. The sample has been recorded in 470 MHz, $CDCl_3$ at 25 °C.

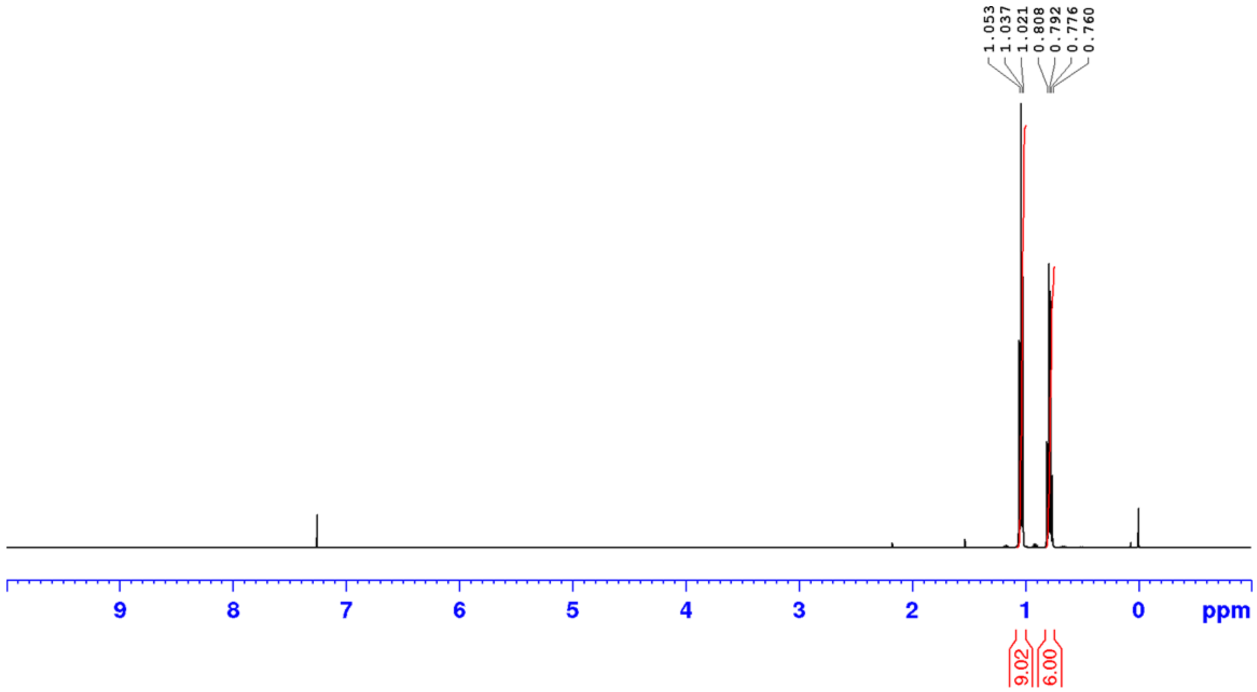

Supplementary Figure 72. ^{1}H NMR of compound 1v. The sample has been recorded in 500 MHz, $CDCl_3$ at 25 °C.

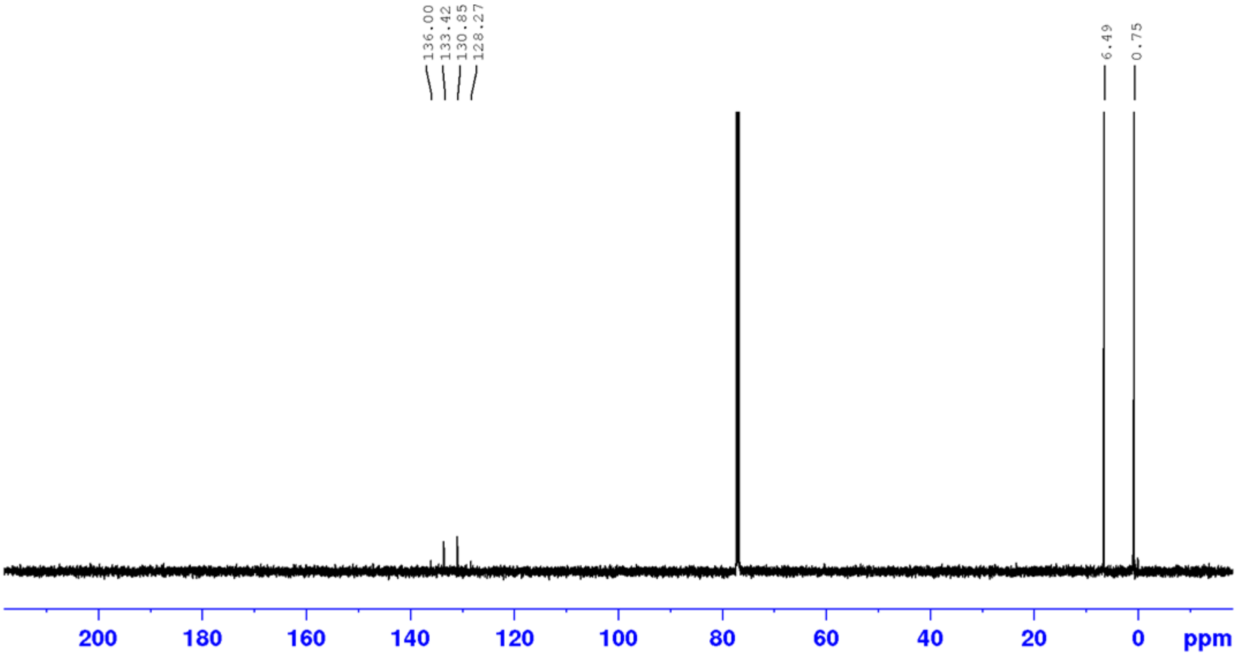

Supplementary Figure 73. ^{13}C NMR of compound 1v. The sample has been recorded in 125 MHz, $CDCl_3$ at 25 °C.

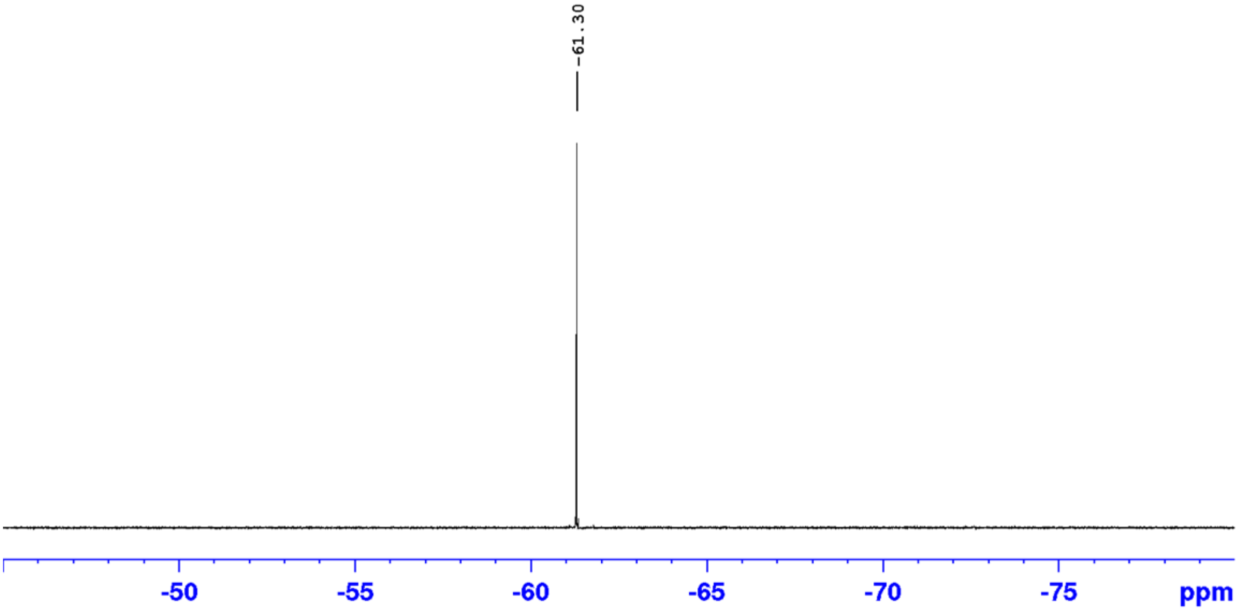

Supplementary Figure 74. ^{19}F NMR of compound 1v. The sample has been recorded in 470 MHz, $CDCl_3$ at 25 °C.

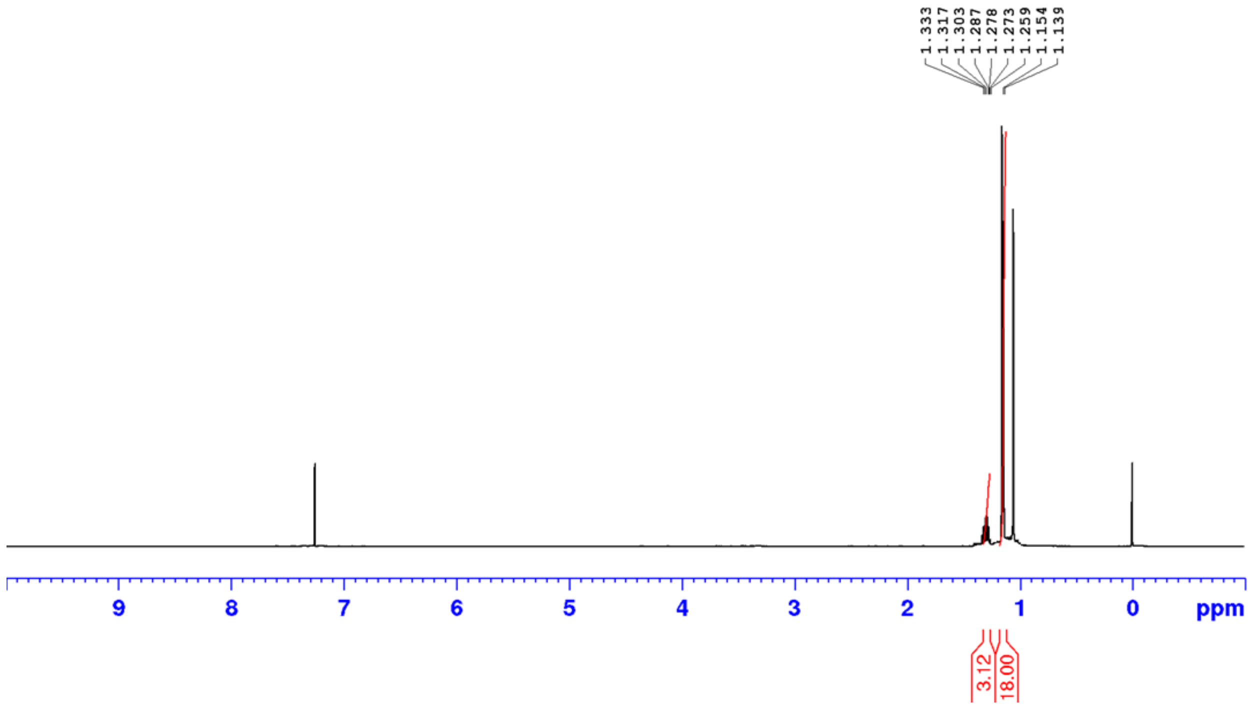

Supplementary Figure 75. ¹H NMR of compound 1w. The sample has been recorded in 500 MHz, $CDCl_3$ at 25 °C.

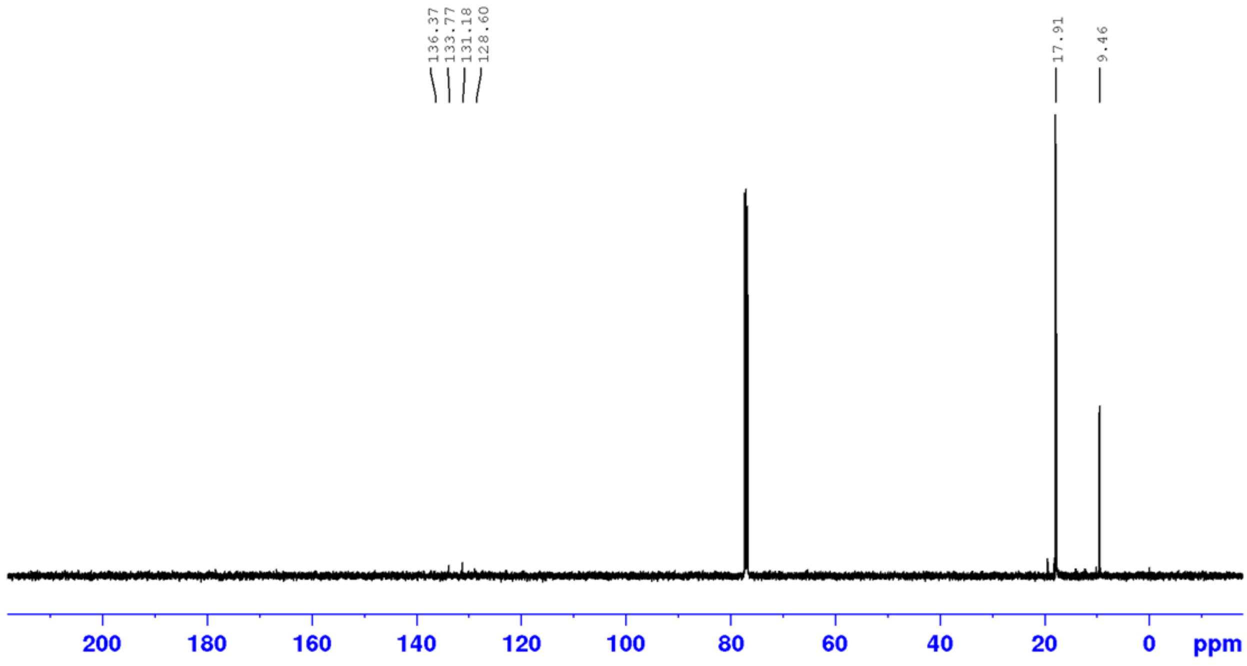

Supplementary Figure 76. ¹³C NMR of compound 1w. The sample has been recorded in 125 MHz, $CDCl_3$ at 25 °C.

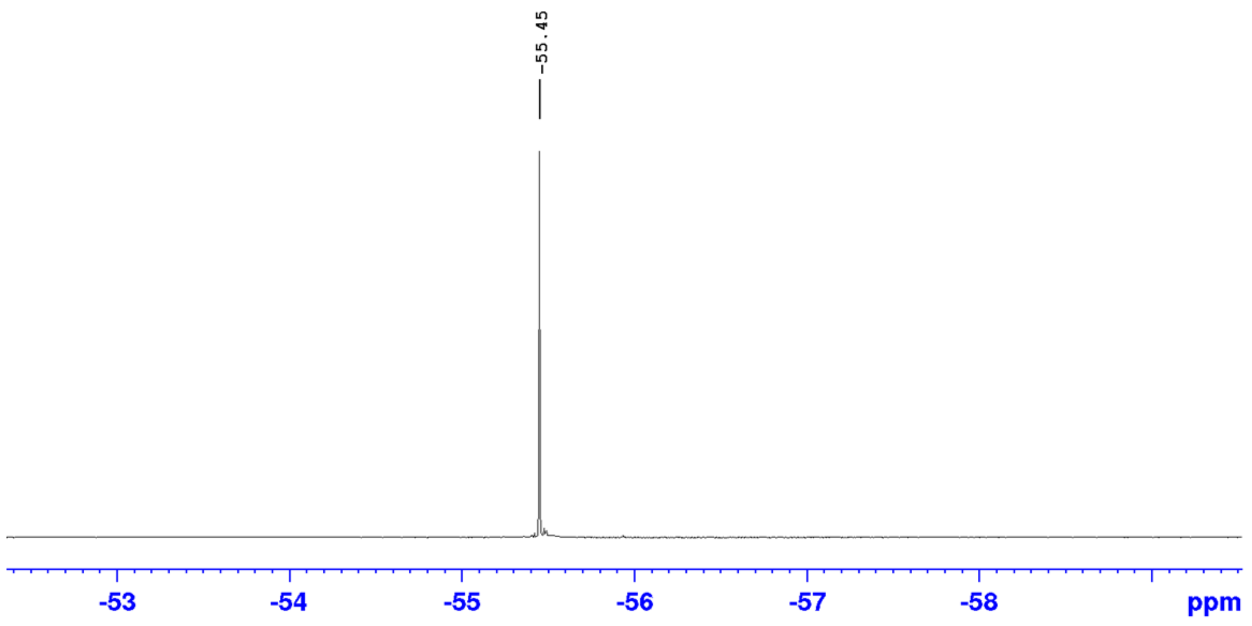

Supplementary Figure 77. ^{19}F NMR of compound 1w. The sample has been recorded in 470 MHz, $CDCl_3$ at 25 °C.

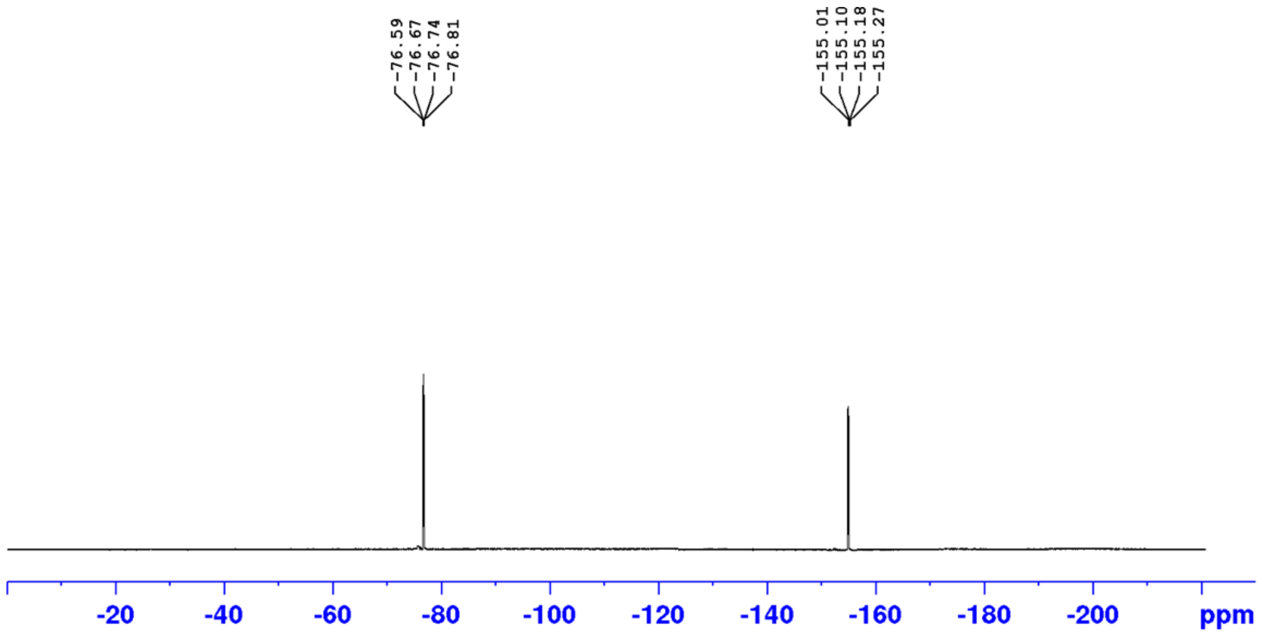

Supplementary Figure 78. ^{19}F NMR of compound 1x. The sample has been recorded in 470 MHz, $CDCl_3$ at 25 °C.

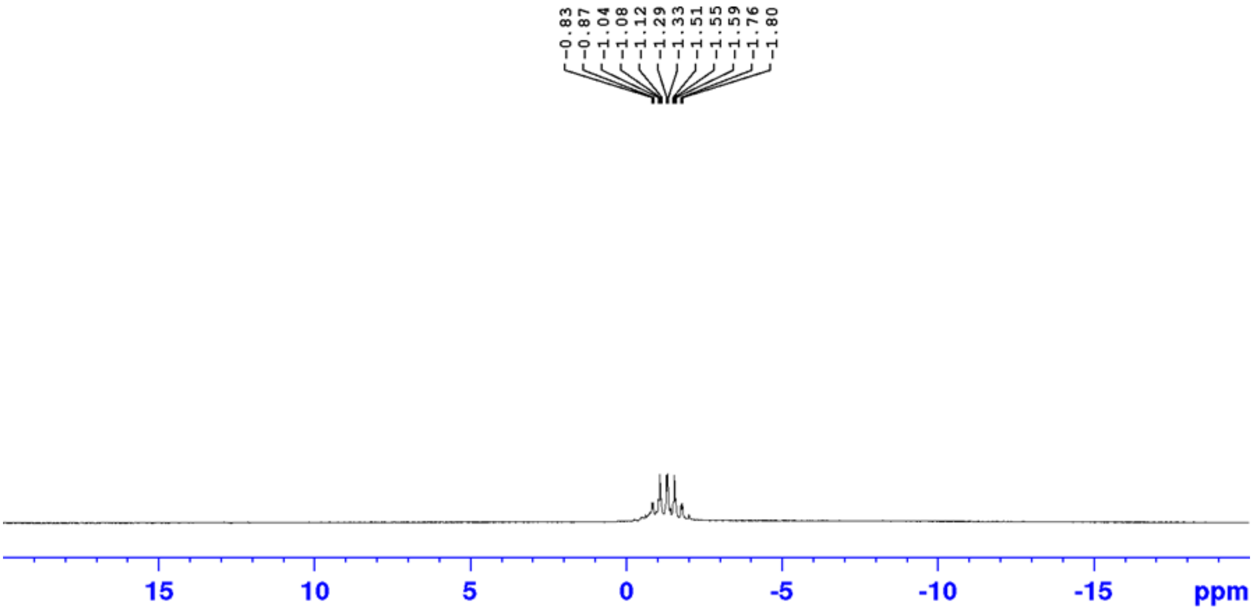

Supplementary Figure 79. ^{11}B NMR of compound 1x. The sample has been recorded in 160 MHz, D_2O at 25 °C.

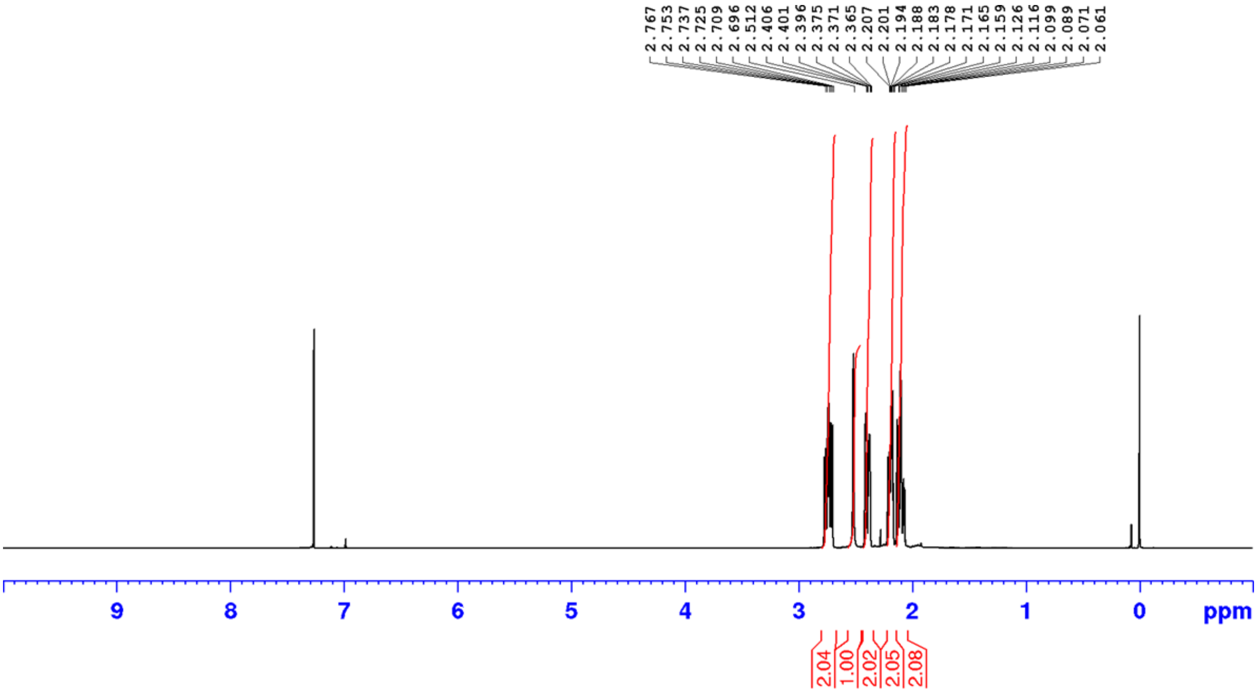

Supplementary Figure 80. ^{1}H NMR of compound 3a. The sample has been recorded in 500 MHz, $CDCl_3$ at 25 °C.

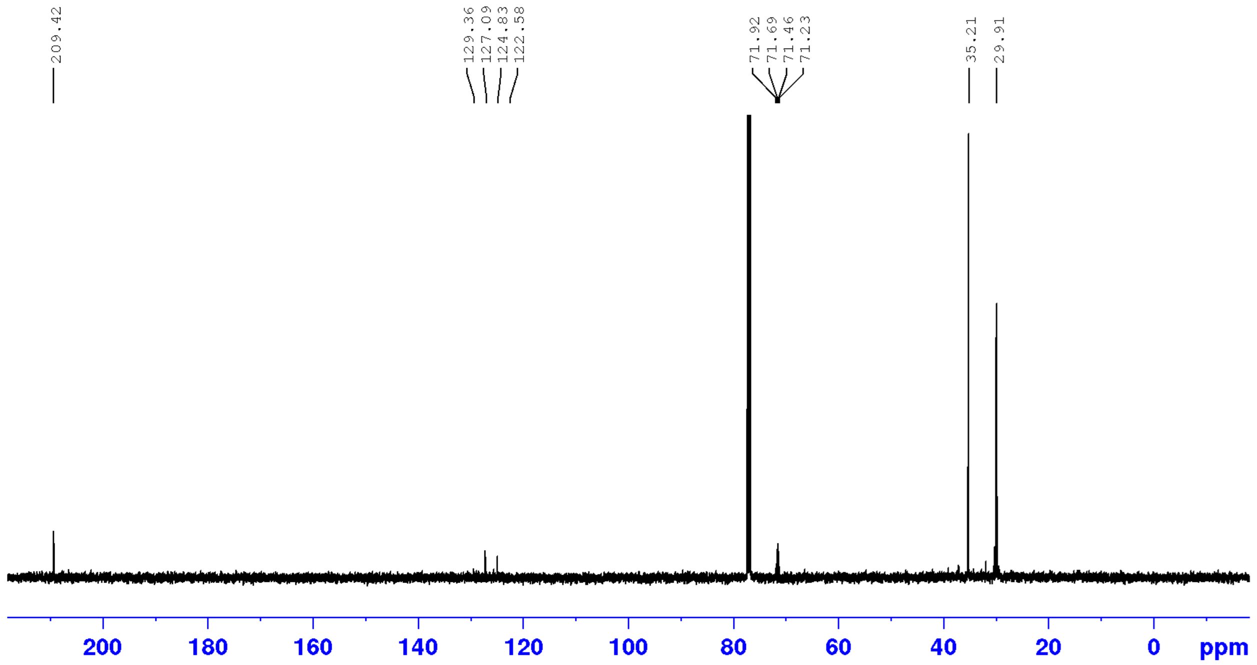

Supplementary Figure 81. ^{13}C NMR of compound 3a. The sample has been recorded in 125 MHz, $CDCl_3$ at 25 °C.

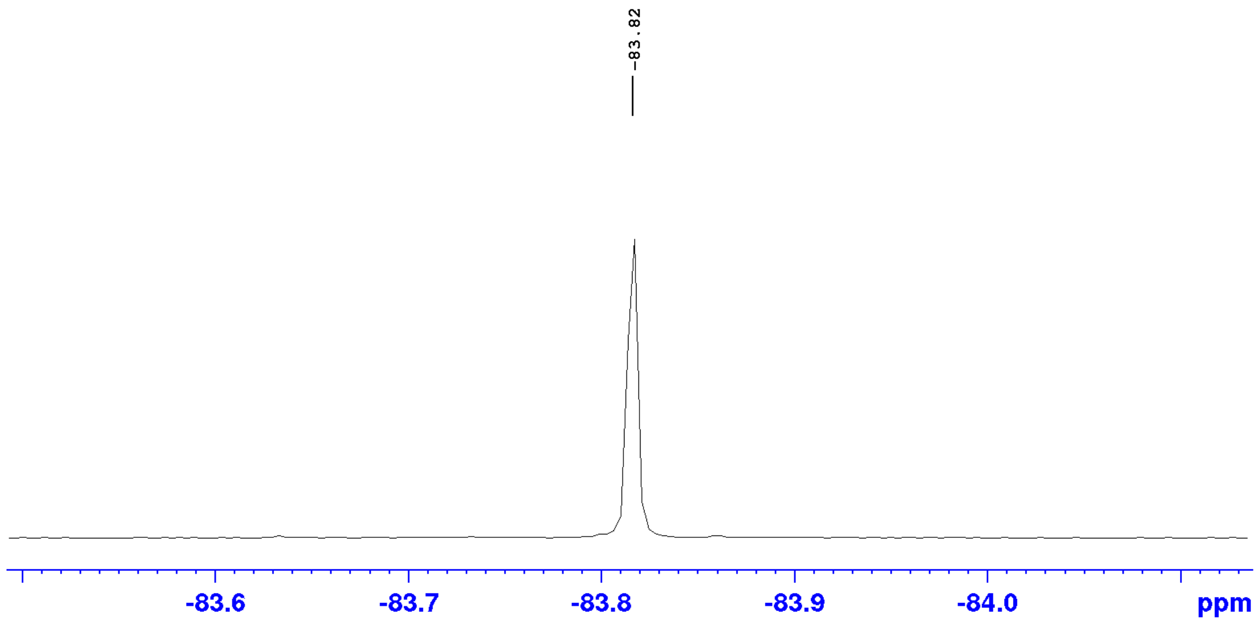

Supplementary Figure 82. ^{19}F NMR of compound 3a. The sample has been recorded in 470 MHz, $CDCl_3$ at 25 °C.

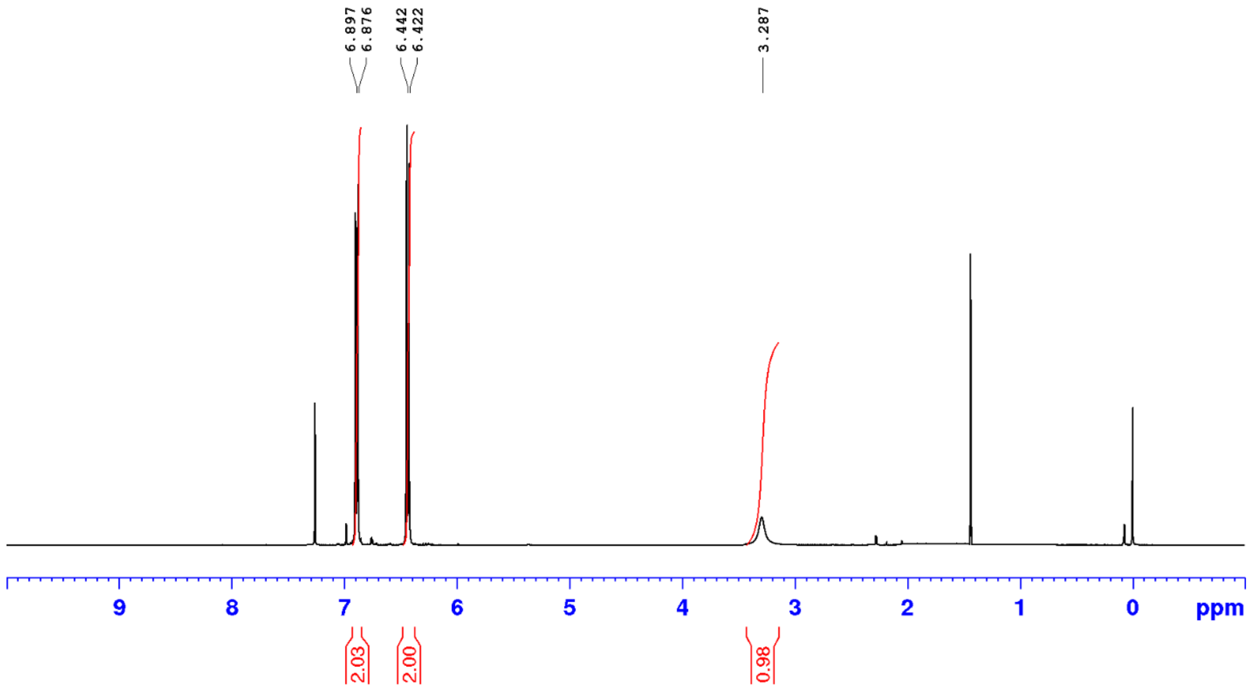

Supplementary Figure 83. ^{1}H NMR of compound 3b. The sample has been recorded in 500 MHz, $CDCl_3$ at 25 °C.

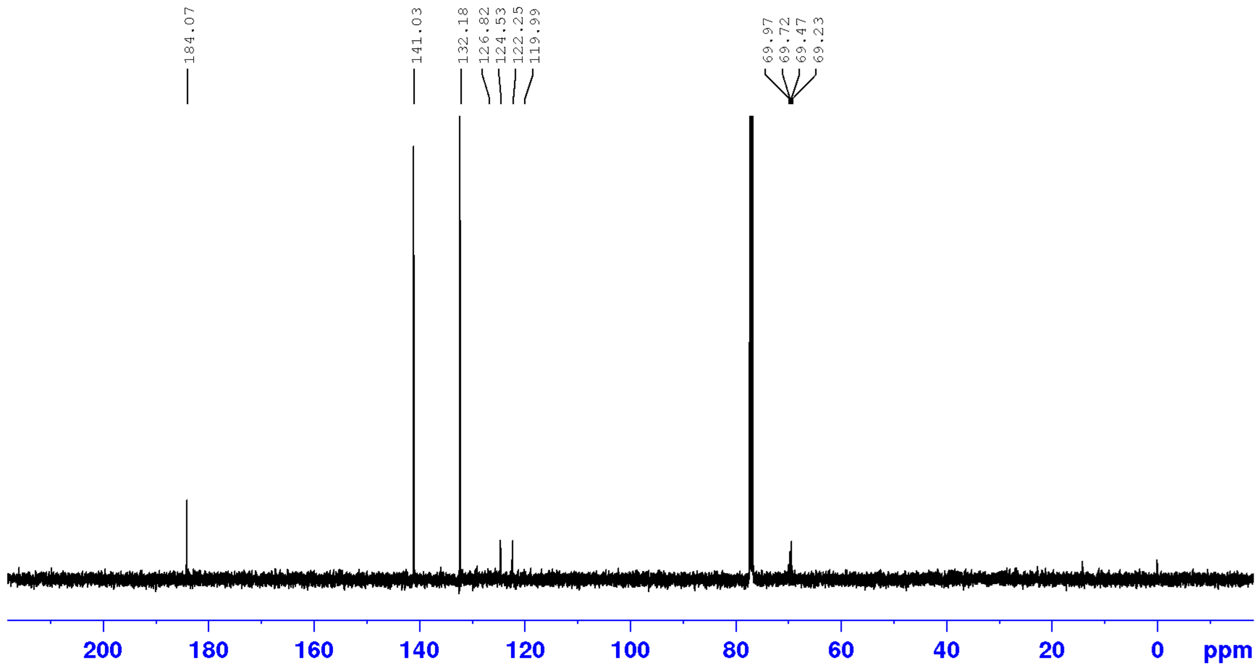

Supplementary Figure 84. ^{13}C NMR of compound 3b. The sample has been recorded in 125 MHz, $CDCl_3$ at 25 °C.

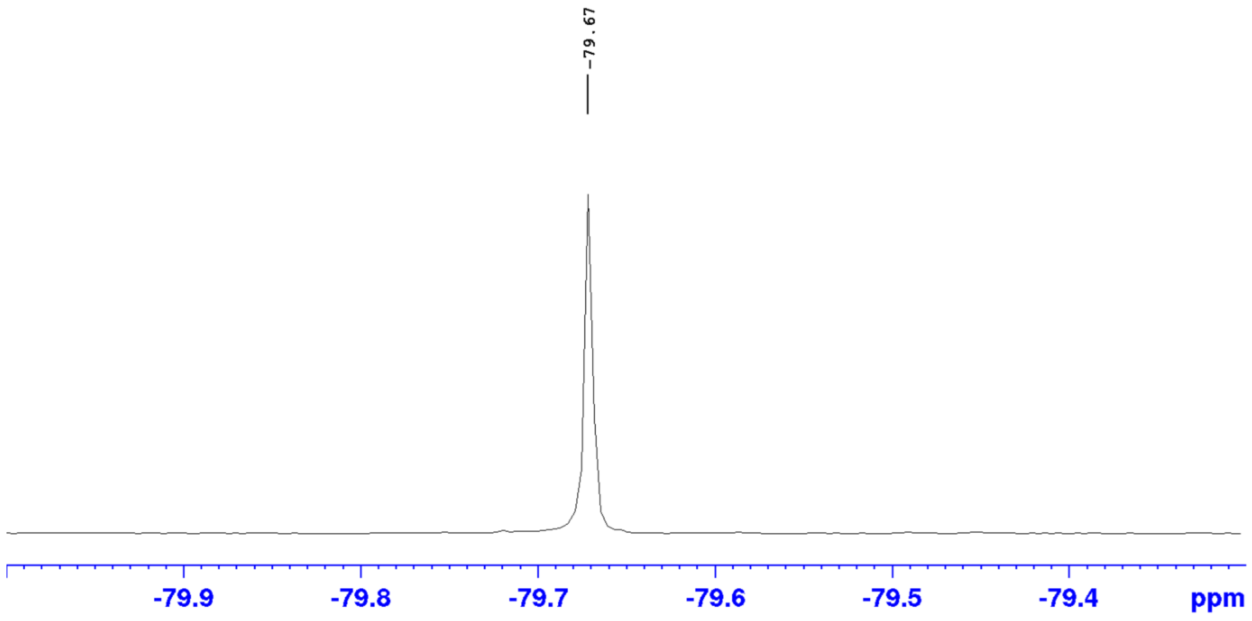

Supplementary Figure 85. ^{19}F NMR of compound 3b. The sample has been recorded in 470 MHz, $CDCl_3$ at 25 °C.

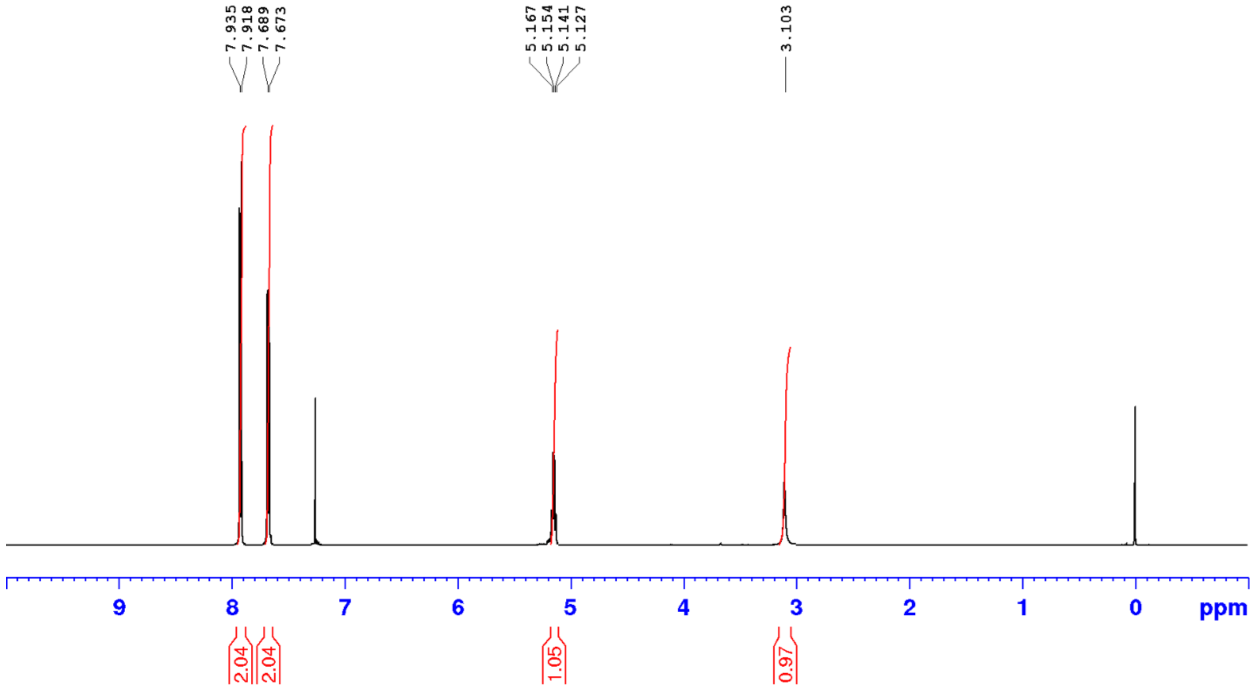

Supplementary Figure 86. ^{1}H NMR of compound 3c. The sample has been recorded in 500 MHz, $CDCl_3$ at 25 °C.

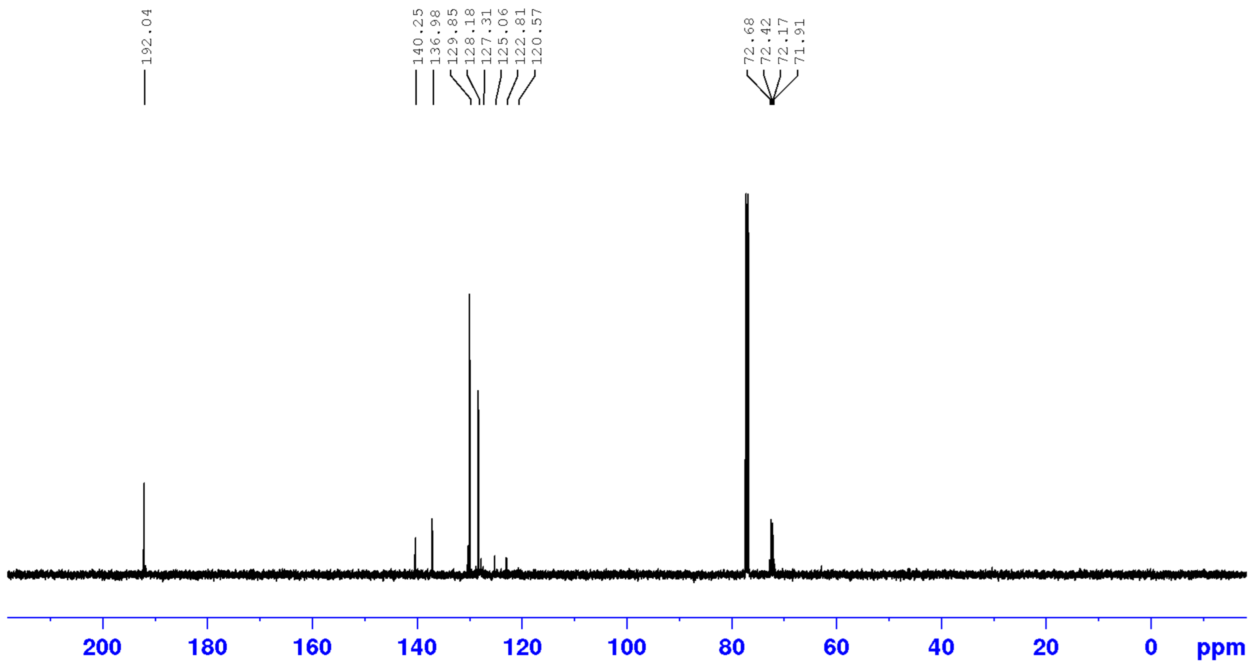

Supplementary Figure 87. ^{13}C **NMR of compound 3c.** The sample has been recorded in 125 MHz, $CDCl_3$ at 25 °C.

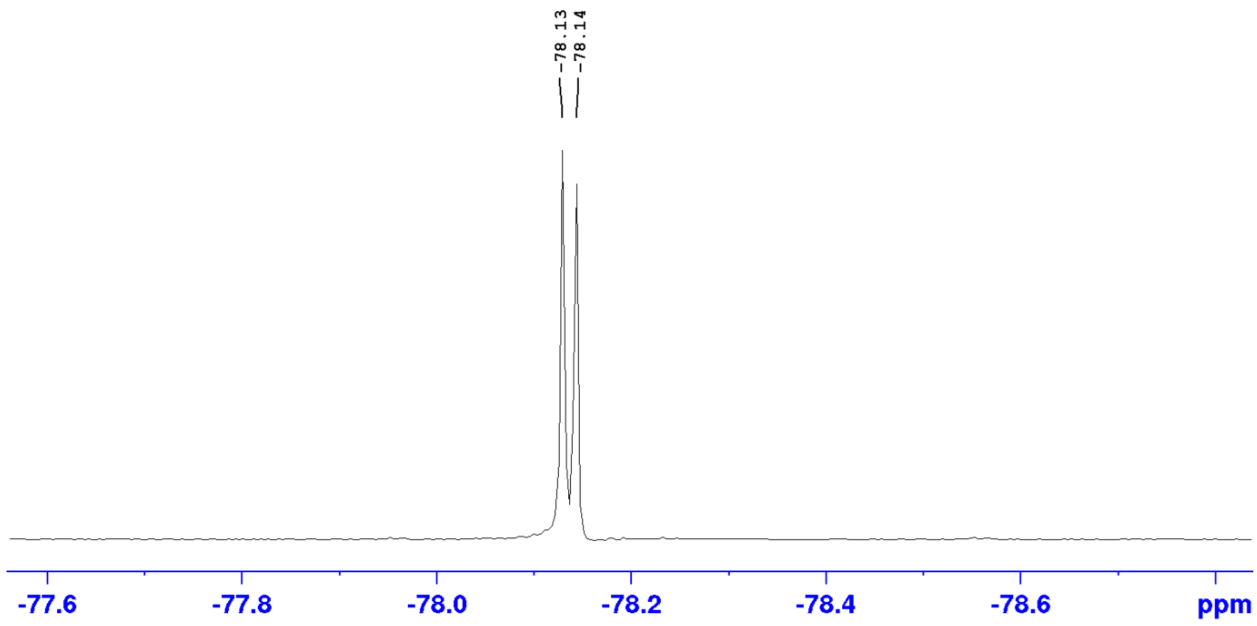

Supplementary Figure 88. ^{19}F **NMR of compound 3c.** The sample has been recorded in 470 MHz, $CDCl_3$ at 25 °C.

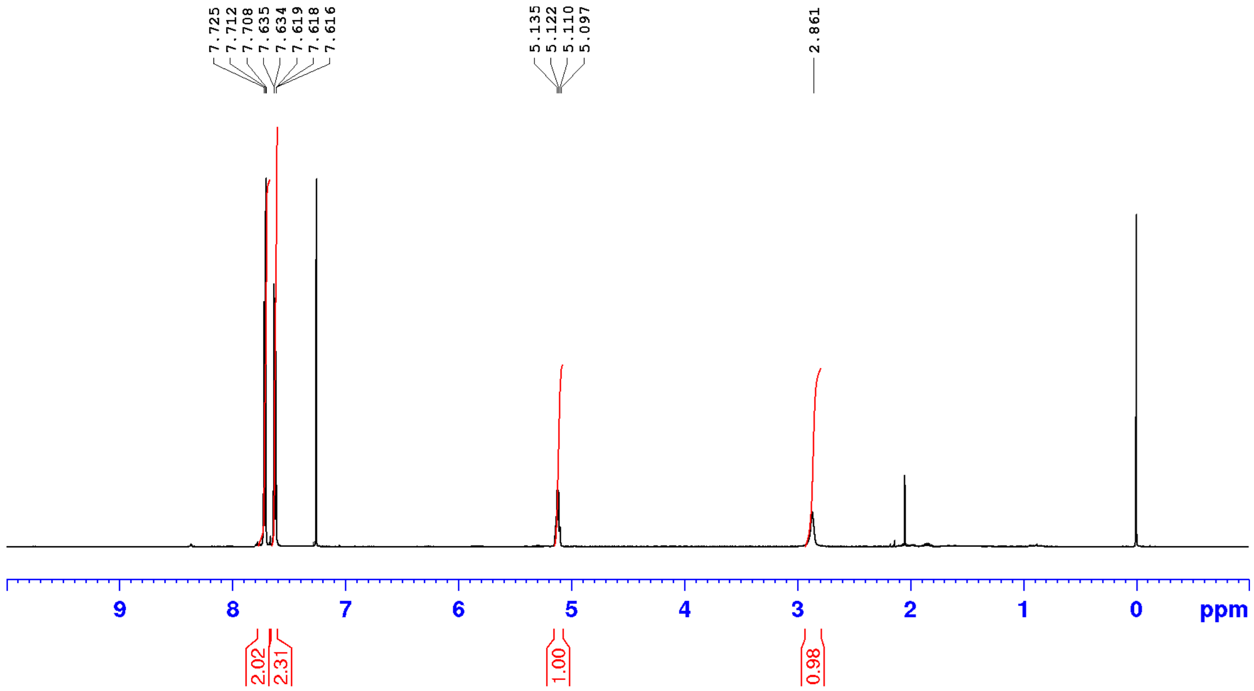

Supplementary Figure 89. ^{1}H NMR of compound 3d. The sample has been recorded in 500 MHz, $CDCl_3$ at 25 °C.

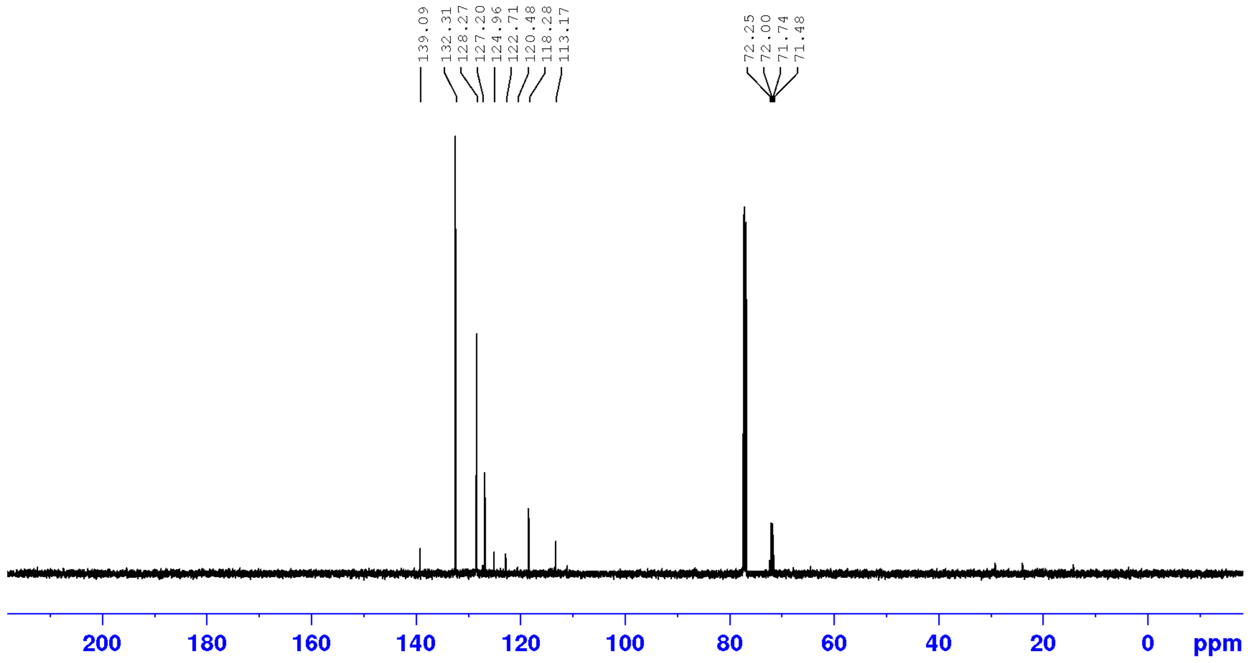

Supplementary Figure 90. ^{13}C NMR of compound 3d. The sample has been recorded in 125 MHz, $CDCl_3$ at 25 °C.

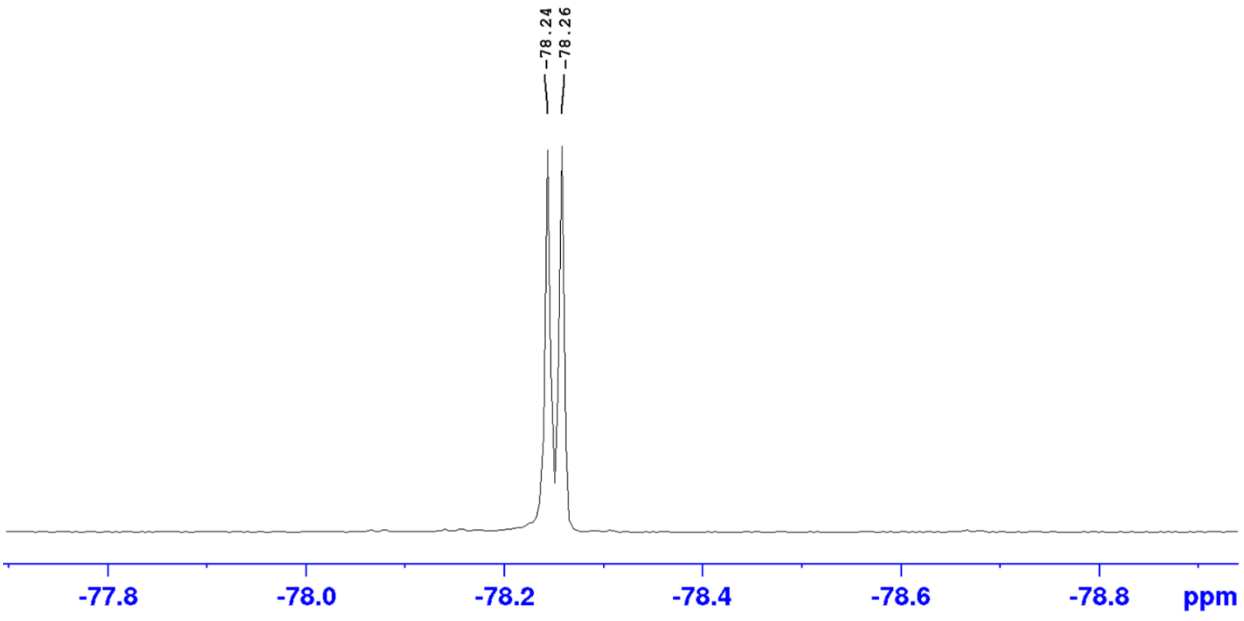

Supplementary Figure 91. ^{19}F NMR of compound 3d. The sample has been recorded in 470 MHz, $CDCl_3$ at 25 °C.

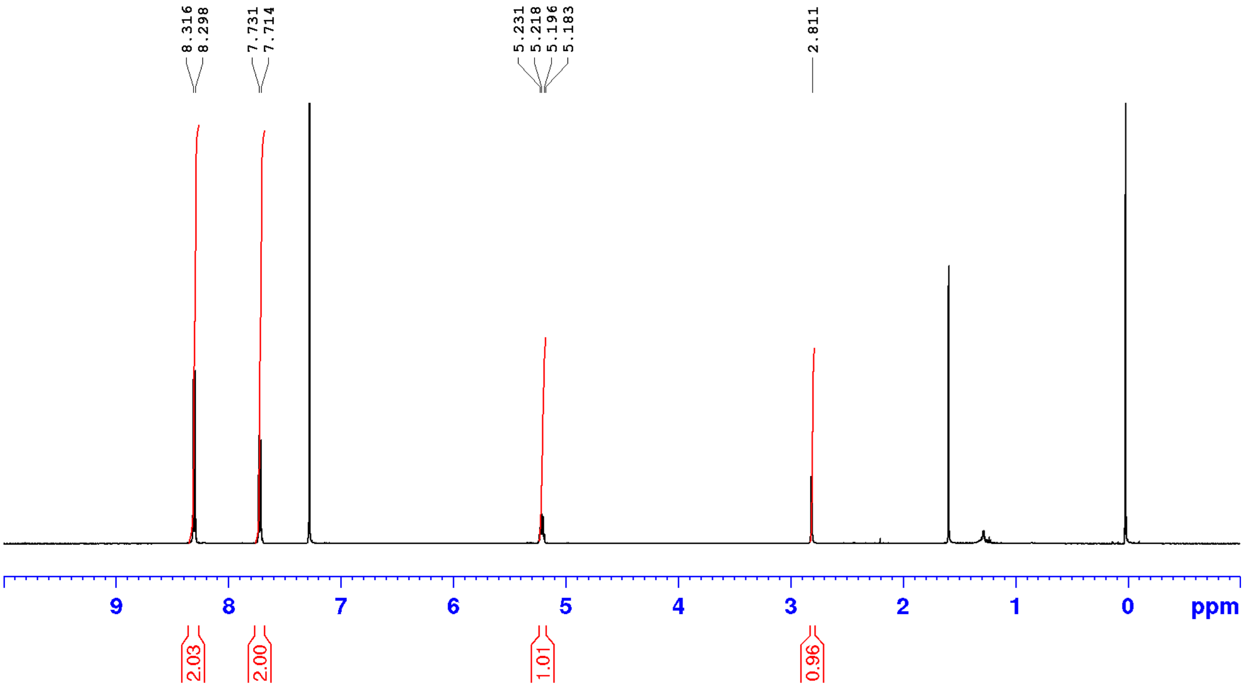

Supplementary Figure 92. ^{1}H NMR of compound 3e. The sample has been recorded in 500 MHz, $CDCl_3$ at 25 °C.

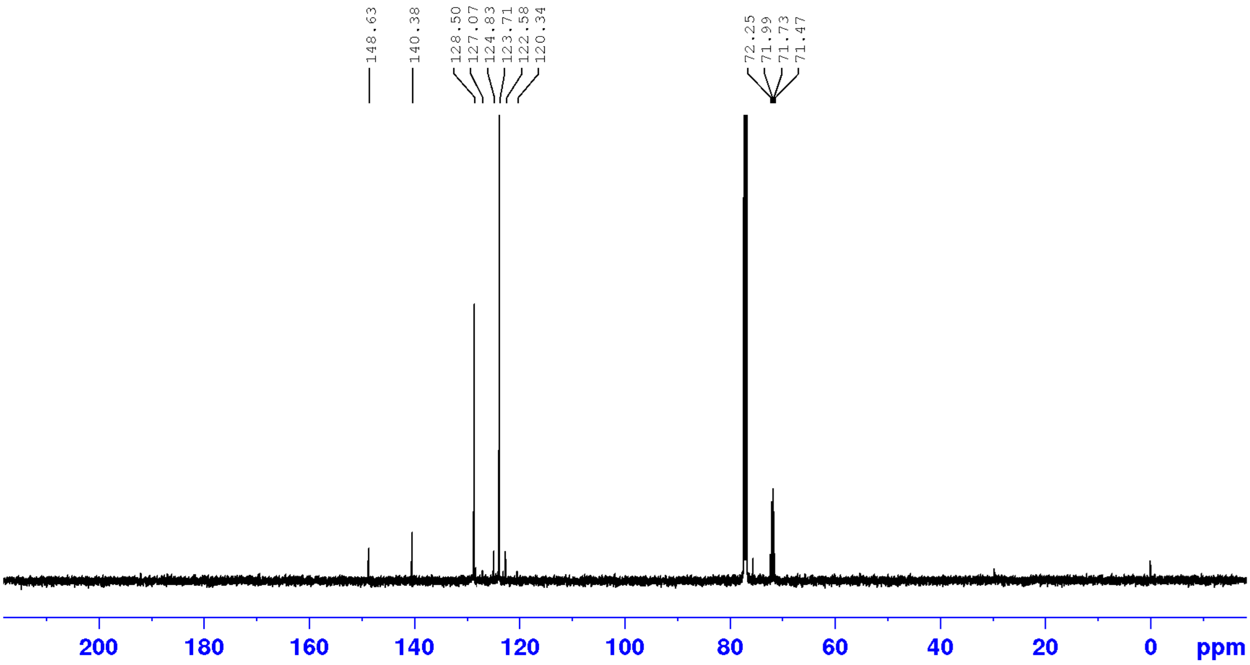

Supplementary Figure 93. ^{13}C NMR of compound 3e. The sample has been recorded in 125 MHz, $CDCl_3$ at 25 °C.

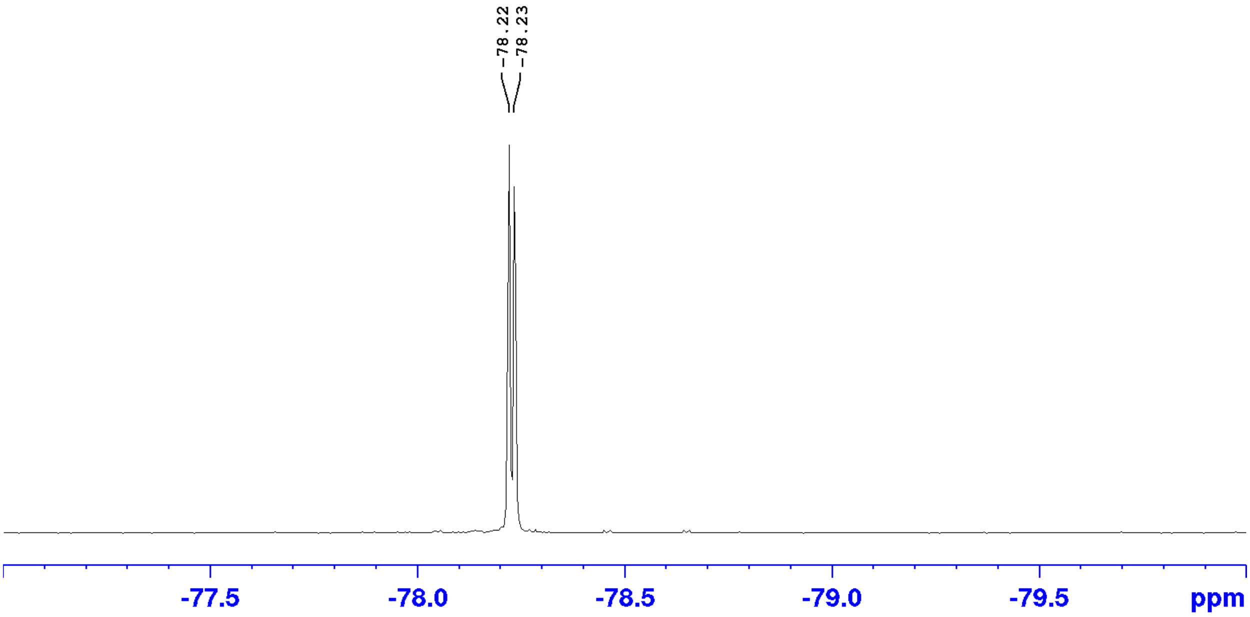

Supplementary Figure 94. ^{19}F NMR of compound 3e. The sample has been recorded in 470 MHz, $CDCl_3$ at 25 °C.

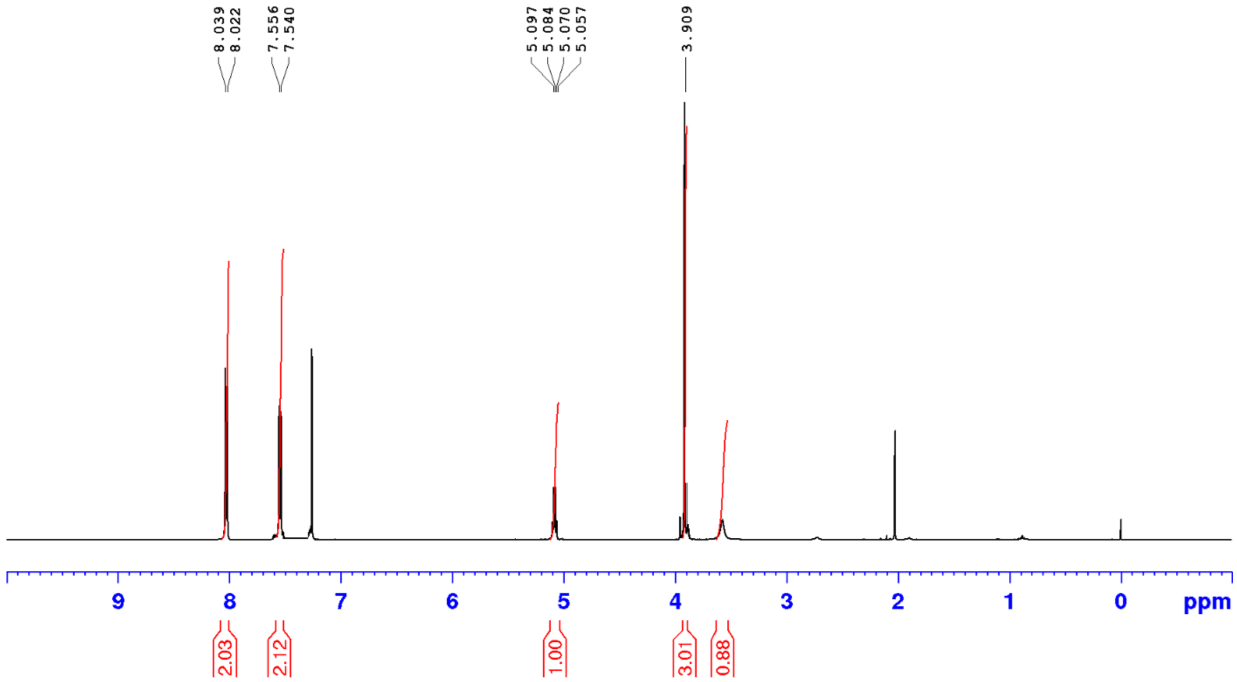

Supplementary Figure 95. ^{1}H NMR of compound 3f. The sample has been recorded in 500 MHz, $CDCl_3$ at 25 °C.

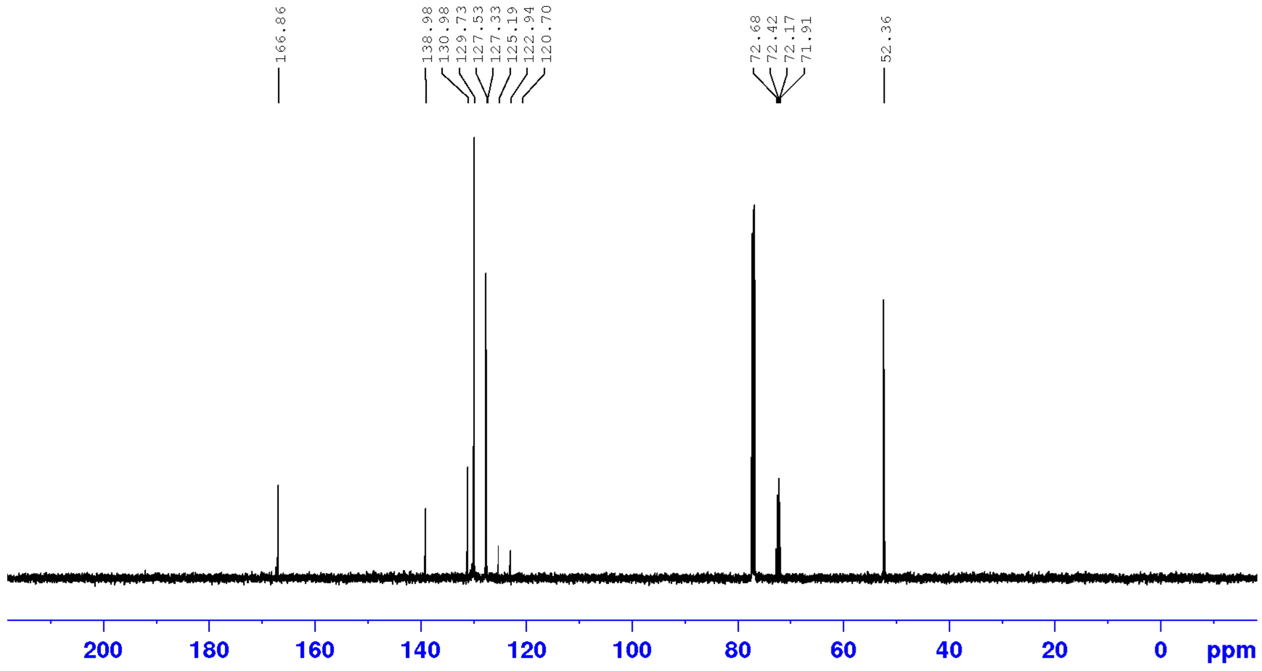

Supplementary Figure 96. ^{13}C NMR of compound 3f. The sample has been recorded in 125 MHz, $CDCl_3$ at 25 °C.

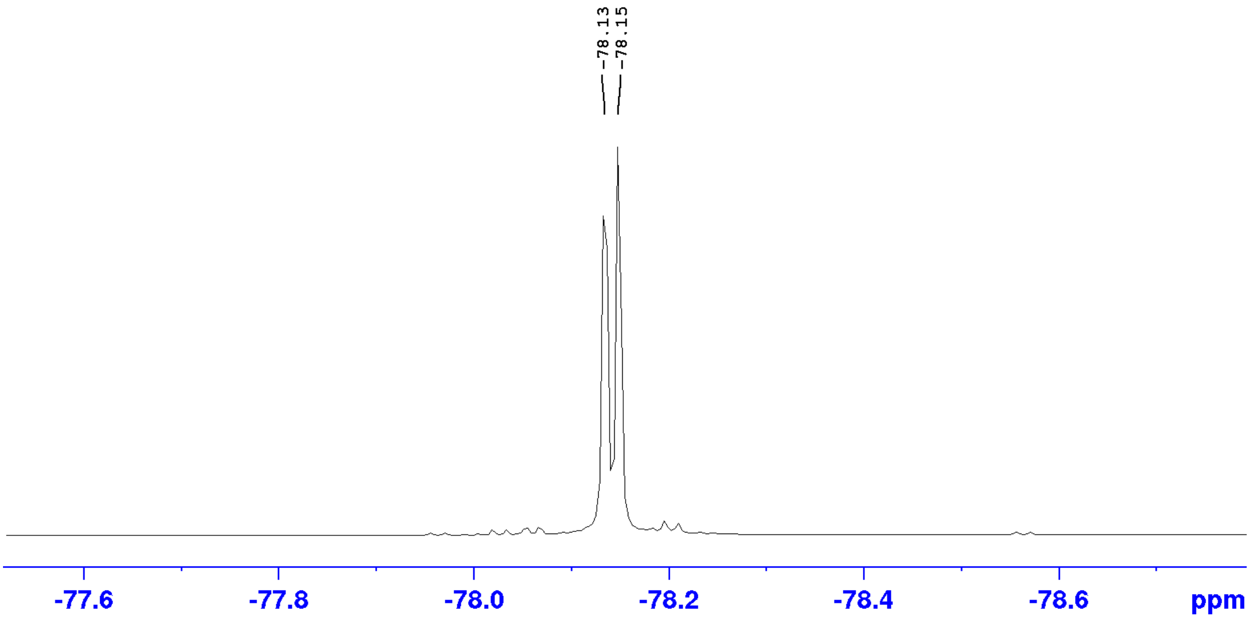

Supplementary Figure 97. ^{19}F NMR of compound 3f. The sample has been recorded in 470 MHz, $CDCl_3$ at 25 °C.

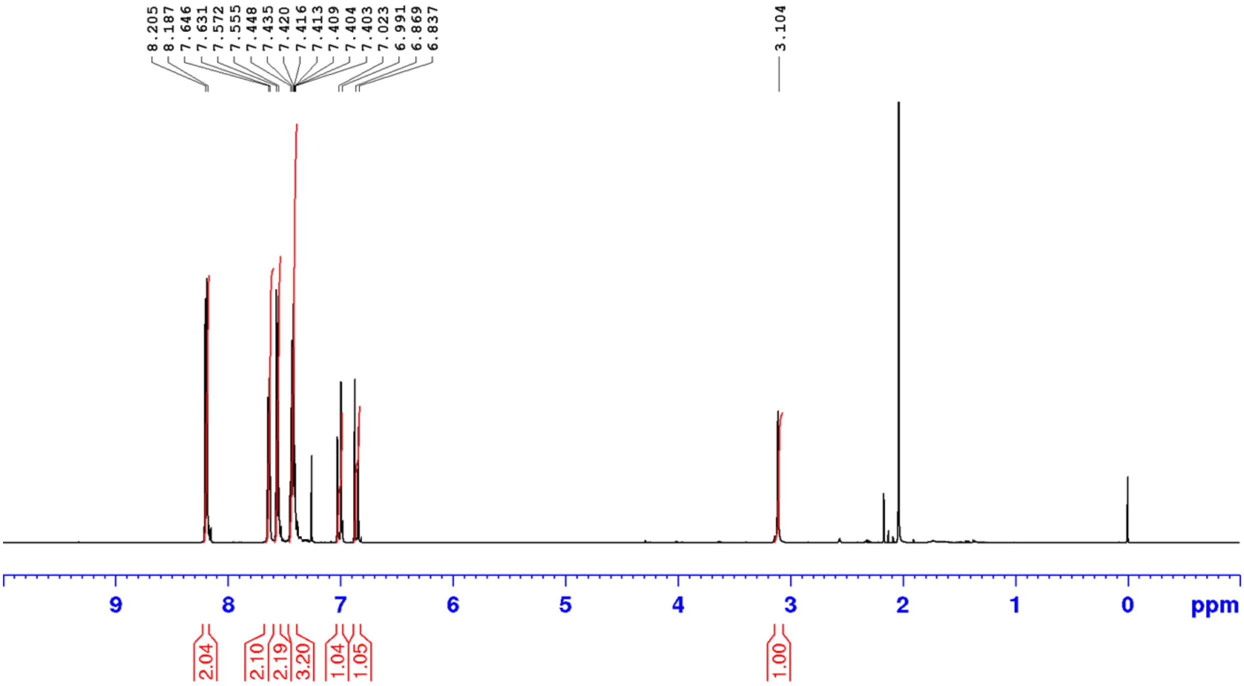

Supplementary Figure 98. ^{1}H NMR of compound 3g. The sample has been recorded in 500 MHz, $CDCl_3$ at 25 °C.

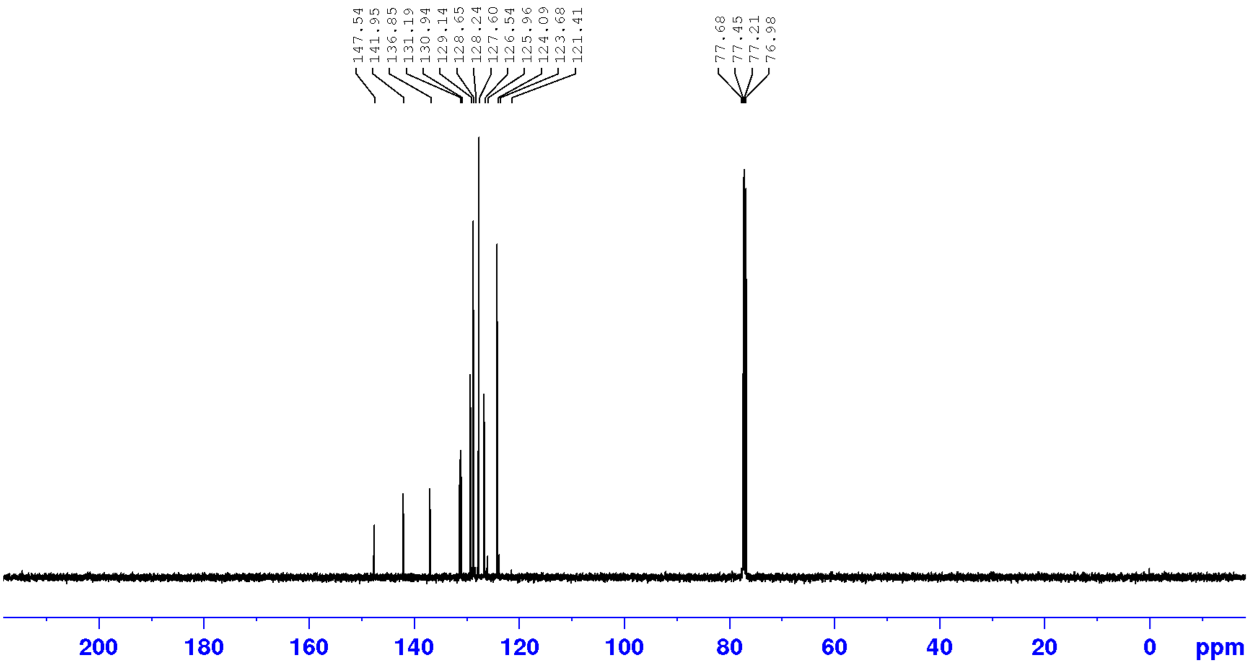

Supplementary Figure 99. ^{13}C NMR of compound 3g. The sample has been recorded in 125 MHz, $CDCl_3$ at 25 °C.

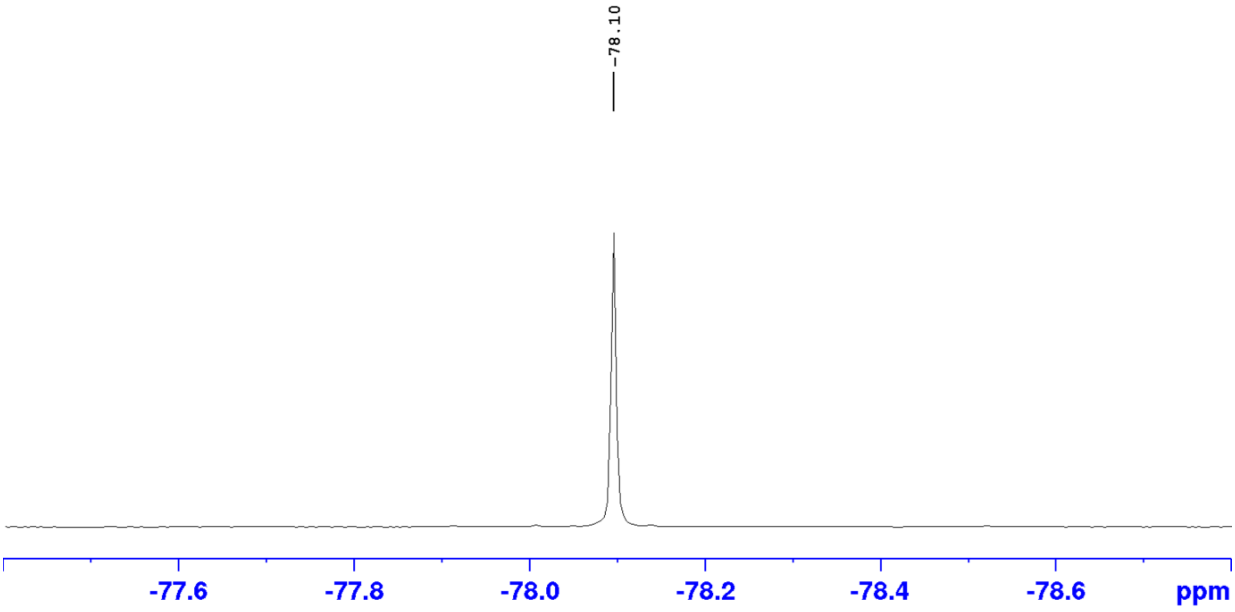

Supplementary Figure 100. ^{19}F NMR of compound 3g. The sample has been recorded in 470 MHz, $CDCl_3$ at 25 °C.

IV. References

1. Li, W.-L. et al. CFD analysis on the intensified mechanism of gas-liquid mass transfer in a microporou s tube-in-tube microchannel reactor. *Int. J. Heat Mass Transf.* **182**, 121914 (2022).
2. Macdonald, I., El-Sayed, M., Mow, K. & Dullien, F. Flow through porous media-the Ergun equation r evisited. *Ind. Eng. Chem. Fundamen.* **18**, 199-208 (1979).
3. Vesvikar, M.S. & Al-Dahhan, M. Flow pattern visualization in a mimic anaerobic digester using CFD. *Biotechnol. Bioeng.* **89**, 719-732 (2005).
4. Sharma, B.M., Yim, S.-J., Nikam, A., Ahn, G.-N. & Kim, D.-P. One-flow upscaling neutralization of an organophosphonate-derived pesticide/nerve agent simulant to value-added chemicals in a novel Tef lon microreactor platform. *React. Chem. Eng.* **6**, 1454-1461 (2021).
5. Chaouk, H., Wilkie, J.S., Meijs, G.F. & Cheng, H.Y. New porous perfluoropolyether membranes. *J. A ppl. Polym. Sci.* **80**, 1756-1763 (2001).
6. Zhang, J. *et al.* Ultrathin microporous membrane with high oil intrusion pressure for effective oil/ water separation. *J. Membr. Sci.* **608**, 118201 (2020).
7. Zhong, G., Liu, D. & Zhang, J. The application of ZIF-67 and its derivatives: adsorption, separation, e lectrochemistry and catalysts. *J. Mater. Chem. A.* **6**, 1887-1899 (2018).
8. Choi, E. et al. CO 2-selective zeolitic imidazolate framework membrane on graphene oxide nanoribbo ns: experimental and theoretical studies. *J. Mater. Chem. A.* **9**, 25595-25602 (2021).
9. Lebl, R. et al. Catalytic Static Mixer-Enabled Hydrogenation of a Key Fenebrutinib Intermediate: Real -Time Analysis for a Stable and Scalable Process. *Org. Process Res. Dev.* **25**, 1988-1995 (2021).
10. Prakash, G.S., Jog, P.V., Batamack, P.T. & Olah, G.A. Taming of fluoroform: direct nucleophilic trifl uoromethylation of Si, B, S, and C centers. *Science* **338**, 1324-1327 (2012).
11. White, J.R., Price, G.J., Plucinski, P.K. & Frost, C.G. The rhodium-catalysed 1, 2-addition of arylboro nic acids to aldehydes and ketones with sulfonated S-Phos. *Tetrahedron Lett.* **50**, 7365-7368 (2009).
12. Saito, T., Wang, J., Tokunaga, E., Tsuzuki, S. & Shibata, N. Direct nucleophilic trifluoromethylation o f carbonyl compounds by potent greenhouse gas, fluoroform: Improving the reactivity of anionoid trifl uoromethyl species in glymes. *Sci. Rep.* **8**, 11501 (2018).
13. Brüning, F., Nagae, H., Käch, D., Mashima, K. & Togni, A. Asymmetric hydrogenation of aryl perflu oroalkyl ketones catalyzed by rhodium (III) monohydride complexes bearing josiphos ligands. *Chem. Eur. J.* **25**, 10818-10822 (2019).
14. Okusu, S., Hirano, K., Yasuda, Y., Tokunaga, E. & Shibata, N. Flow trifluoromethylation of carbonyl compounds by Ruppert–Prakash reagent and its application for pharmaceuticals, efavirenz and HSD-0 16. *RSC Adv.* **6**, 82716-82720 (2016).
15. Yong, K.H. & Chong, J.M. Enantioselective reduction of trifluoromethyl ketones with chiral organom agnesium amides (COMAs). *Org. Lett.* **4**, 4139-4142 (2002).

16. Xu, Q., Zhou, H., Geng, X. & Chen, P. Nonenzymatic kinetic resolution of racemic 2, 2, 2-trifluoro-1-aryl ethanol via enantioselective acylation. *Tetrahedron* **65**, 2232-2238 (2009).
17. Augurusa, A., Mehta, M., Perez, M., Zhu, J. & Stephan, D.W. Catalytic reduction of amides to amines by electrophilic phosphonium cations via FLP hydrosilylation. *Chem. Commun.* **52**, 12195-12198 (2016).
18. Hu, B.-L., Song, Y.-K., Zhang, G., Yao, Z. & Zhang, X.-G. Copper-catalyzed three component CS/CN coupling for the synthesis of trifluorothioacetamides. *J. Fluorine Chem.* **239**, 109640 (2020).
19. Martynov, M.Y., Iakovenko, R.O., Kazakova, A.N., Boyarskaya, I.A. & Vasilyev, A.V. Acid-promoted cyclization of 2, 4-diaryl-1, 1, 1-trifluorobut-3-en-2-oles and their TMS-ethers into CF3-indenes. *Org. Biomol. Chem.* **15**, 2541-2550 (2017).
20. Saito, T., Wang, J., Tokunaga, E., Tsuzuki, S. & Shibata, N. Direct nucleophilic trifluoromethylation of carbonyl compounds by potent greenhouse gas, fluoroform: Improving the reactivity of anionoid trifluoromethyl species in glymes. *Sci. Rep.* **8**, 11501 (2018).
21. Jones, D.H. et al. Synthesis of dibenzylamino-1-methylcyclohexanol and dibenzylamino-1-trifluoromethylcyclohexanol isomers. *Org. Biomol. Chem.* **14**, 172-182 (2016).
22. Large, S., Roques, N. & Langlois, B.R. Nucleophilic trifluoromethylation of carbonyl compounds and disulfides with trifluoromethane and silicon-containing bases. *J. Org. Chem.* **65**, 8848-8856 (2000).
23. Kelly, C.B., Mercadante, M.A., Hamlin, T.A., Fletcher, M.H. & Leadbeater, N.E. Oxidation of α-Trifluoromethyl alcohols using a recyclable oxoammonium salt. *J. Org. Chem.* **77**, 8131-8141 (2012).
24. Sprenger, J.A., Kerpen, C., Ignat'ev, N. & Finze, M. Convenient synthesis of perfluoroalkyltrifluoroborates. *J. Fluorine Chem.* **206**, 54-60 (2018).
